# A New Morphological Phylogeny of the Ophiuroidea (Echinodermata) Accords with Molecular Evidence and Renders Microfossils Accessible for Cladistics

**DOI:** 10.1371/journal.pone.0156140

**Published:** 2016-05-26

**Authors:** Ben Thuy, Sabine Stöhr

**Affiliations:** 1 Department of Palaeontology, Natural History Museum Luxembourg, Luxembourg-city, Luxembourg; 2 Department of Zoology, Swedish Museum of Natural History, Stockholm, Sweden; Muséum national d'Histoire naturelle, FRANCE

## Abstract

Ophiuroid systematics is currently in a state of upheaval, with recent molecular estimates fundamentally clashing with traditional, morphology-based classifications. Here, we attempt a long overdue recast of a morphological phylogeny estimate of the Ophiuroidea taking into account latest insights on microstructural features of the arm skeleton. Our final estimate is based on a total of 45 ingroup taxa, including 41 recent species covering the full range of extant ophiuroid higher taxon diversity and 4 fossil species known from exceptionally preserved material, and the Lower Carboniferous *Aganaster gregarius* as the outgroup. A total of 130 characters were scored directly on specimens. The tree resulting from the Bayesian inference analysis of the full data matrix is reasonably well resolved and well supported, and refutes all previous classifications, with most traditional families discredited as poly- or paraphyletic. In contrast, our tree agrees remarkably well with the latest molecular estimate, thus paving the way towards an integrated new classification of the Ophiuroidea. Among the characters which were qualitatively found to accord best with our tree topology, we selected a list of potential synapomorphies for future formal clade definitions. Furthermore, an analysis with 13 of the ingroup taxa reduced to the lateral arm plate characters produced a tree which was essentially similar to the full dataset tree. This suggests that dissociated lateral arm plates can be analysed in combination with fully known taxa and thus effectively unlocks the extensive record of fossil lateral arm plates for phylogenetic estimates. Finally, the age and position within our tree implies that the ophiuroid crown-group had started to diversify by the Early Triassic.

## Introduction

Brittle stars or ophiuroids are a major component of modern marine benthic communities, occurring in all oceans from the tropics to the poles and from the intertidal to the hadal trenches. With over 2000 living species, they are the largest among the five extant echinoderm classes [1]. Yet, the evolutionary history that shaped present-day ophiuroid distribution and diversity patterns is still surprisingly understudied. Apart from occasional attempts at family-level classification, the monographs of the mid- and late nineteenth century which laid the foundation for ophiuroid systematics provided little more than anatomical observations and a plethora of new genera and species.

First attempts to reconstruct a comprehensive higher-level classification and explore the evolutionary history of the Ophiuroidea were made by Matsumoto [2,3]. On the basis of a few key skeletal characters, in particular vertebral morphology, the presence of dorsal arm plates and the type of articulation between the genital plate and the radial shield, Matsumoto [2,3] subdivided the Ophiuroidea into two subclasses, the Oegophiuroida for the Paleozoic forms with divided vertebrae, and the Myophiuroida with fused vertebrae, comprising all extant forms. The latter were further subdivided into four orders accommodating all the families and subfamilies known at that time: the Phrynophiurida (including the euryalids and Ophiomyxidae), Laemophiurida (Ophiacanthidae and Hemieuryalidae), Gnathophiurida (Amphiuridae, Amphilepididae and Ophiotrichidae) and the Chilophiurida (Ophiodermatidae, Ophiochitonidae, Ophiocomidae, Ophioleucidae and Ophiolepididae).

Matsumoto’s work remained the only serious overall analysis of ophiuroid phylogeny and was challenged only on few occasions. Mortensen [4], for instance, insisted on dividing modern ophiuroids into the orders Euryalae and Ophiurae. Fell [5] initially followed this logic but later endorsed Matsumoto’s classification [6], with a few modifications: he raised the Ophiomyxidae to suborder level within the Phrynophiurida along with the Euryalina, and grouped all other extant ophiuroids in the order Ophiurida with Matsumoto’s suborders Chilophiurina, Laemophiurina and Gnathophiurina, except for the aberrant genus *Ophiocanops* Koehler, 1922 which he considered the only living representative of the Oegophiurida [6,7]. Fell’s classification was adopted by Spencer and Wright [8] and remained largely unchallenged for several decades.

The first and, to date, only attempt at disentangling the higher-level classification and evolutionary history of the Ophiuroidea based on morphological evidence and using modern phylogenetic methods was made by Smith *et al*. [9]. Their results endorse the subdivision of modern ophiuroids into two orders, the Euryalida and the Ophiurida, with the latter comprising the suborders Ophiomyxina and Ophiurina. Matsumoto’s legacy with respect to family concepts and characters evidently shaped the analysis and the resulting subdivision of the Ophiurina, which, apart from the Ophiacanthidae as a paraphyletic complex, comprised the infra-orders Hemieuryalina (Hemieuryalidae), Chilophiurina (Ophiuridae), Gnathophiurina (Amphilepididae, Ophiocomidae, Ophionereididae, Amphiuridae, Ophiactidae and Ophiotrichidae), Ophiodermatina (Ophiochitonidae and Ophiodermatidae) and the Ophiolepidina (Ophiolepididae). With respect to the position of *Ophiocanops*, Smith *et al*. [9] followed Fell [6,7] and considered it a member of the otherwise Paleozoic subclass Oegophiuridea and thus sister to all other living ophiuroids. Recently, the Euryalida have received greater attention, confirming the monophyly of this group and proposing the Asteroschematinae and Astrocharinae as subfamilies of Euryalidae [10], shortly thereafter as families [11]. The World Ophiuroidea Database [12] currently recognizes 19 families within Ophiuroidea, treating Ophioleucinae as subfamily of the Ophiuridae following Smith et al. [9], whereas O'Hara et al. [13] considered them on family level.

Thus, ophiuroid classification has not dramatically evolved since Matsumoto’s [2,3] pioneering work, which is to a considerable extent the result of uncritically adopted traditional family (and subfamily) concepts. This is particularly obvious in the study of Smith *et al*. [9] who scored morphological characters on the basis of family-level averages whenever different members of a family displayed contrasting character states. They also extracted characters mostly from the literature, resulting in several misconceptions [14]. With at most 20 families and a traditionally constrained concept of euryalid versus non-euryalid relationships, the range of possible classification schemes has been intrinsically limited so far. The growing number of new, unconventional taxa (e.g. [15,16]) and novel morphological observations (e.g. [17,18]) fundamentally challenging previous classification schemes, however, highlights the necessity for a revision of existing concepts of ophiuroid phylogeny.

The clash between traditional ophiuroid systematics and state-of-the-art understanding of their phylogeny recently culminated in the transcriptome-based analysis by O’Hara *et al*. [13] which showed just how deadlocked previous classification concepts were. In fact, it suggested three primary ophiuroid clades which refute all previous morphology-based classifications of the Ophiuroidea. In particular, it unambiguously showed that euryalids form a clade with the Ophiuridae and *Ophiomusium* Lyman, 1869, rather than being sister to all non-euryalids, and that many traditional families (e.g. Ophiolepididae, Ophiocomidae, Ophiomyxidae) are polyphyletic. The most important finding, however, was that general structural skeleton characters as previously understood failed to reflect phylogenetic relationships. Instead, the new phylogeny of O’Hara *et al*. [13] is in astonishing congruence with classification schemes based on recently described microstructural characters, in particular those pertaining to the lateral arm plates [17,18]. The landmark study by O’Hara et al. [13] was recently followed by a phylogenetic estimate using rRNA gene sequences [19] which, however, produced largely inconclusive results due to limited taxon and gene sampling. For our purpose, the reference of choice in terms of molecular phylogeny therefore remains the study by O’Hara et al. [13].

Here, we attempt a long overdue recast of a morphology-based phylogeny of the Ophiuroidea. By re-assessing traditional characters and including novel microstructural features [17,18], we explore ophiuroid phylogeny from a cutting-edge morphological perspective, complementing the recent molecular evidence and providing an analytical basis for the definition of synapomorphies. More specifically, our analysis intends to test the content of phylogenetically informative characters in lateral arm plates and their microstructural features as recently anticipated [13]. Ultimately, the aim of the study is to pave the way towards a new synapomorphy-based ophiuroid classification backed by molecular evidence and allowing full use of the ophiuroid fossil record including lateral arm plates preserved as microfossils.

## Materials and Methods

### Taxon sampling

Our analysis is confined to extant ophiuroids and their direct fossil relatives, which are here informally called modern ophiuroids. Extinct ophiuroid groups with unfused vertebrae and/or ambulacral groove spines are omitted. From the 19 currently accepted families of modern ophiuroids, we sampled all 14 non-euryalid families and three of the five euryalid families, namely the Gorgonocephalidae, Euryalidae and Asteronychidae. Our dataset includes the families Hemieuryalidae, Amphilepididae, Ophiohelidae and Euryalidae, which were omitted by O'Hara et al. [13]. The Asteroschematidae and Astrocharidae were omitted. In fact, since the euryalids have recently been shown to be a well-defined, monophyletic group [10,11], in-group sampling was kept at a level allowing to assess the position of the euryalids without compromising the overall taxon/character ratio.

In order to minimize the bias inherent to previous ophiuroid classifications, the analysis is based on individual species rather than families. A total of 46 species were sampled (Table 1), including one outgroup taxon. At least two representatives were selected per family, including the type genus, to adequately cover the diversity of the family concept as traditionally understood. As an exception to this rule, the following families were represented by their type genus only: the three euryalid families for the in-group sampling constraints outlined above, the Ophiotrichidae because they are morphologically rather uniform, and the Amphilepididae because they include only one very rare genus aside the type genus. Also, the type genus of Ophiohelidae is an extremely rare deep-sea taxon and has not been available for examination. Within the limits of specimen availability, we attempted to sample the type species of each genus.

**Table 1:** List of species sampled for the phylogenetic analysis.

Species	Author	Family Assignment
*Aganaster gregarius*†	(Meek & Worthen, 1869)	Ophiolepididae
*Asteronyx loveni	Müller & Troschel, 1842	Asteronychidae
*Gorgonocephalus caputmedusae	(Linnaeus, 1758)	Gorgonocephalidae
*Euryale aspera	Lamarck, 1816	Euryalidae
*Ophiomyxa pentagona	(Lamarck, 1816)	Ophiomyxidae
*Ophioscolex glacialis*	Müller & Troschel, 1842	Ophiomyxidae
*Ophiolycus purpureus*	(Düben & Koren, 1846)	Ophiomyxidae
*Ophiacantha bidentata	(Bruzelius, 1805)	Ophiacanthidae
*Ophiolimna bairdi*	(Lyman, 1883)	Ophiacanthidae
*Ophiocopa spatula*	Lyman, 1883	Ophiacanthidae
*Ophiotreta valenciennesi*	(Lyman, 1879)	Ophiacanthidae
*Ophiochondrus stelliger*	Lyman, 1879	Ophiacanthidae
*Inexpectacantha acrobatica*†	Thuy, 2011	Ophiacanthidae
*Ophienigma spinilimbatum*	Stöhr & Ségonzac, 2005	Ophiacanthidae
*Ophiomyces delata*	Koehler, 1904	Ophiohelidae
*Ophiotholia spathifer*	(Lyman, 1879)	Ophiohelidae
*Aplocoma agassizi*†	(von Münster, 1839)	Ophiolepididae
*Ophiolepis superba	H.L. Clark, 1915	Ophiolepididae
*Ophiozonella longispina*	(H.L. Clark, 1908)	Ophiolepididae
*Ophiomusium lymani*	Wyville-Thomson, 1873	Ophiolepididae
*Ophioderma longicauda	(Bruzelius, 1805)	Ophiodermatidae
*Ophiarachna incrassata*	(Lamarck, 1816)	Ophiodermatidae
*Palaeocoma milleri*†	(Phillips, 1829)	Ophiodermatidae
*Ophiura ophiura	(Linnaeus, 1758)	Ophiuridae (Ophiurinae)
*Ophiocten sericeum*	(Forbes, 1852)	Ophiuridae (Ophiurinae)
*Ophiosparte gigas*	Koehler, 1922	Ophiuridae (Ophiurinae)
*Ophioleuce seminudum	Koehler, 1904	Ophiuridae (Ophioleucinae)
*Ophiopallas paradoxa*	Koehler, 1904	Ophiuridae (Ophioleucinae)
*Eirenura papillata*†	Thuy, 2011	Ophiuridae (Ophioleucinae)
*Ophiochiton fastigatus	Lyman, 1878	Ophiochitonidae
*Ophioplax lamellosa*	Matsumoto, 1915	Ophiochitonidae
*Ophionereis porrecta*	Lyman, 1860	Ophionereididae
*Ophiodoris malignus*	Koehler, 1904	Ophionereididae
*Hemieuryale pustulata	Von Martens, 1867	Hemieuryalidae
*Sigsbeia murrhina*	Lyman, 1878	Hemieuryalidae
*Amphilepis norvegica	(Ljungman, 1865)	Amphilepididae
*Ophiocoma echinata	(Lamarck, 1816)	Ophiocomidae
*Ophiocomina nigra*	(Abildgaard, in O.F. Müller, 1789)	Ophiocomidae
*Ophiopsila guineensis*	Koehler, 1914	Ophiocomidae
*Amphiura chiajei	Forbes, 1843	Amphiuridae
*Amphioplus congensis*	(Studer, 1882)	Amphiuridae
*Amphilimna olivacea*	(Lyman, 1869)	Amphiuridae
*Ophiactis savignyi	(Müller & Troschel, 1842)	Ophiactidae
*Histampica duplicata*	(Lyman, 1875)	Ophiactidae
*Ophiopholis aculeata*	(Linnaeus, 1767)	Ophiactidae
*Ophiothrix fragilis	(Abildgaard, in O.F. Müller, 1789)	Ophiotrichidae

Family-level assignment of the sampled species are given according to Smith *et al*. [9] with modifications by Hotchkiss and Haude [22], Martynov [17], Thuy *et al*. [21] and Parameswaran *et al*. [23]. Family types are preceded by an asterisk, fossil ones are marked by a cross.

In addition, the following recent taxa were selected for being the subject of ongoing controversy over their systematic position: *Amphilimna* Verrill, 1899, *Ophienigma* Stöhr & Segonzac, 2005, *Ophiocomina* (Abildgaard, in O.F. Müller, 1789), and *Ophiosparte* Koehler, 1922. In order to bridge some potentially deep divergences between extant taxa and to provide potential calibration points for an evolutionary tree, four fossil taxa were included, all known from both exceptionally preserved articulated individuals and dissociated skeletal plates: *Aplocoma* d’Orbigny, 1852, from the Middle and Late Triassic of Europe [20] with specimens from the Late Triassic of the Netherlands [21]; *Eirenura* Thuy, 2011, *Inexpectacantha* Thuy, 2011, and *Palaeocoma* d’Orbigny, 1950.

The well-known Paleozoic basal crown group ophiuroid *Aganaster gregarius* (Meek & Worthen, 1869) was chosen as outgroup taxon at the expense of a stem-group member (e.g. [9]) for its assumed proximity to the ingroup [22]. The choice of a Carboniferous modern-type ophiuroid gains support from the evolutionary tree by O’Hara *et al*. [13] which dated the crown-group ophiuroid origin to the mid-Permian. *Aganaster gregarius* is known from numerous articulated and mostly well preserved skeletons (e.g. [22]). In addition, previously undescribed dissociated skeletal plates were extracted from a slab containing several articulated specimens, from the Edwardsville Member of the Muldraugh Formation, Borden Group (upper Osagian, Lower Carboniferous) of Corey’s Bluff on Sugar Creek, Crawfordsville, Indiana, kindly donated by F. Hotchkiss on behalf of Marine and Paleobiological Research Institute in 2012.

### Specimen treatment and scoring

Our analysis is strictly specimen-based, for two reasons: first, direct observations on specimens avoid the risk of ambiguous or erroneous descriptions in literature, and second, the analysis specifically focuses on novel characters, many of which have been discovered only in the course of the present study or at least have not been previously documented for the species sampled. Specimens were observed using both a dissecting microscope and scanning electron microscope (SEM) imaging. For the latter purpose, individual skeletal plates were extracted from specimens or parts of specimens immersed in regular household bleach (NaClO), rinsed in tap water, dried, mounted on aluminium stubs and gold-coated. Dissociated skeletal parts of fossil ophiuroids were extracted from the sieving residues of the sediments yielding the articulated specimens (see [24] or [18] for technical details). The majority of the specimens were thus prepared by ourselves, except *Ophiochondrus stelliger*, *Hemieuryale pustulata* and *Sigsbeia murrhina* for which sets of SEM and digital light images were graciously provided by A. Gondim (see also [25]), and *Ophiolepis superba* for which T. Piñeda kindly donated a complete set of images. Our own observations of *Ophiosparte gigas* were complemented with published images from Martynov [17]. We also utilized the online database of the National Museum of Natural History, Smithsonian Institution, Washington DC, to examine images of *Ophiochondrus stelliger* (USNME44767.368449) and *Ophiosparte gigas* (USNM1079135). All specimens prepared by ourselves were deposited either in the Swedish Museum of Natural History (SMNH) or the Natural History Museum Luxembourg (MnhnL) (see S1 Table for voucher numbers).

For our analysis we attempted to extract as many characters as meaningfully possible from hard-part anatomical features discernible in both fossil and recent specimens. Most traditional or previously used characters (e.g. [5,9]), if retained at all, had to be thoroughly redefined or subdivided in order to fully reflect the morphological spectrum of the sampled taxa or to avoid obvious homoplasies. About one third of the characters used in our study pertain to lateral arm plates, hereafter abbreviated LAPs, based on recent observations and non-analytical assessments of plate microstructures [17,18,26]. All characters were treated as of equal weight and unordered in the absence of evidence for a clear ontogenetic or size-related progression between character states.

Scoring was carried out independently by each of us and then compared and checked for consistency. As a result, a number of initial character definitions were refined to avoid potential ambiguity. When character states could not be assessed due to poor preservation or a lack of data, the character was scored with a “?”. If a character was inapplicable in a taxon, e.g. pertaining to a structure which was absent in that particular taxon, the character was scored with a “-“. We used the software Xper2 [27] to assemble our matrix, due to its easy interface and the capability of exporting Nexus formatted files.

### Phylogenetic analysis

The initial data matrix obtained as a result of concerted scoring comprised 184 characters, 66 of which pertained to the LAPs, and was analysed for a first explorative approach using parsimony and Bayesian inference. The parsimony analysis was performed using TNT (freely available through the support of the Willi Hennig Society [28]) for a bootstrapping analysis with 2000, 5000, and 10000 replicates with strict consensus tree and majority rule tree, all of which delivered the same result with regard to tree topology and bootstrap values (scripts and results can be found in S2–S9 Files). We also attempted to find synapomorphies with TNT but that proved highly unsatisfactory. Instead we evaluated synapomorphies by hand (see below).

Bayesian inference analysis was performed using MrBayes [29] as an MCMC simulation with default parameters for morphological data after testing the effect of various parameter changes. MrBayes uses a modified version of the Juke-Cantor model for morphological data as outlined by Lewis [30] with variable character states from 2 to 10 [31,32]. In contrast to molecular studies, only variable characters were sampled, meaning that characters that have the same state for all examined taxa were omitted, and we compensated for character selection bias by letting MrBayes search for parsimony informative characters (Mkpars model [32]. All character states were assumed to have equal frequency, and prior probabilities were equal for all trees. We assumed that evolutionary rates vary between sites according to a discrete gamma distribution. Branch lengths were unconstrained. Runs with eight chains delivered the same result as runs with four chains and changing the temperature of the heated chains had no noticeable effect on tree topologies. Average standard deviations of split frequencies stabilized at about 0.007–0.01 after 3 million generations (mgen), sampled every 1,000 generations. The first 25% of the trees were discarded as burnin. The consensus trees were examined with the software FigTree v. 1.4.2 by Rambaut (http://tree.bio.ed.ac.uk/software/figtree/).

The trees produced by both parsimony and Bayesian inference analyses showed substantially similar topologies, with few minor exceptions. Differences in topology and in the extent of resolution almost exclusively concerned parts of the tree which gained only poor support in both approaches.

Although the initial analyses produced trees which already revealed a considerable amount of structure, character analysis showed that the tree topologies are supported by only a small portion of the characters (less than 30%). This prompted us to critically reassess the characters and the definitions of their states in order to identify cases in which structures that are superficially similar but anatomically different in detail were identically coded. The explorative analyses furthermore suggested that tree resolution and support benefitted from categorisation of complex structures and shapes rather than excessive itemisation of their components. We therefore redefined several characters, in particular those pertaining to the shape of the radial shields, genital plates and the ornamentation of the LAPs, accordingly.

The revised matrix comprises 130 characters (S1 Appendix), including 42 LAP characters. Proportions of unknown character states are very low in general, ranging from 11.54 to 0.0% (mean 1.61%, completeness of the matrix 98.43%). In the fossil species included, a higher portion of characters (mean 4.64%) was missing than in the recent ones (mean 1.25%). Poorest known characters in terms of portion of unknown states across the species were the position and size of the primary radial plates, the presence of a lumen in the arm spines, and the position of mouth papilla 2.

The final 130 character matrix (S1 File) was analysed using Bayesian inference as described above. Bayesian methods have been shown to be more accurate than parsimony for the analysis of discrete morphological characters [32], and since Bayesian and parsimony approaches in our study produced substantially similar results, we refrained from employing both methods. A node was considered well-supported if it gained a posterior probability of at least 90%.

A subset of the matrix, with 13 of the ingroup taxa reduced to the 42 LAP characters, was analysed with Bayesian inference using the settings as described above. The aim of the analysis was to examine the effect of the character-rich LAP structures on the overall topology of the tree, and to simulate the scenario of a combined analysis of taxa known as complete skeletons (recent species and exceptional fossil finds) and taxa known only from isolated LAPs (the majority of the currently known fossil species).

The fundamental clash with previous formal ophiuroid classifications undoubtedly emanates to a major extent from the choice of the characters used. For our analysis we intended to assess the higher-level systematic diversity of the ophiuroid skeletal morphology as comprehensively as possible. We therefore fully drew from the latest insights on microstructural features, in particular those pertaining to the lateral arm plates [17,18], and at the same time critically revised the traditional characters that shouldered previous classifications.

## Results

**Fig 1:**
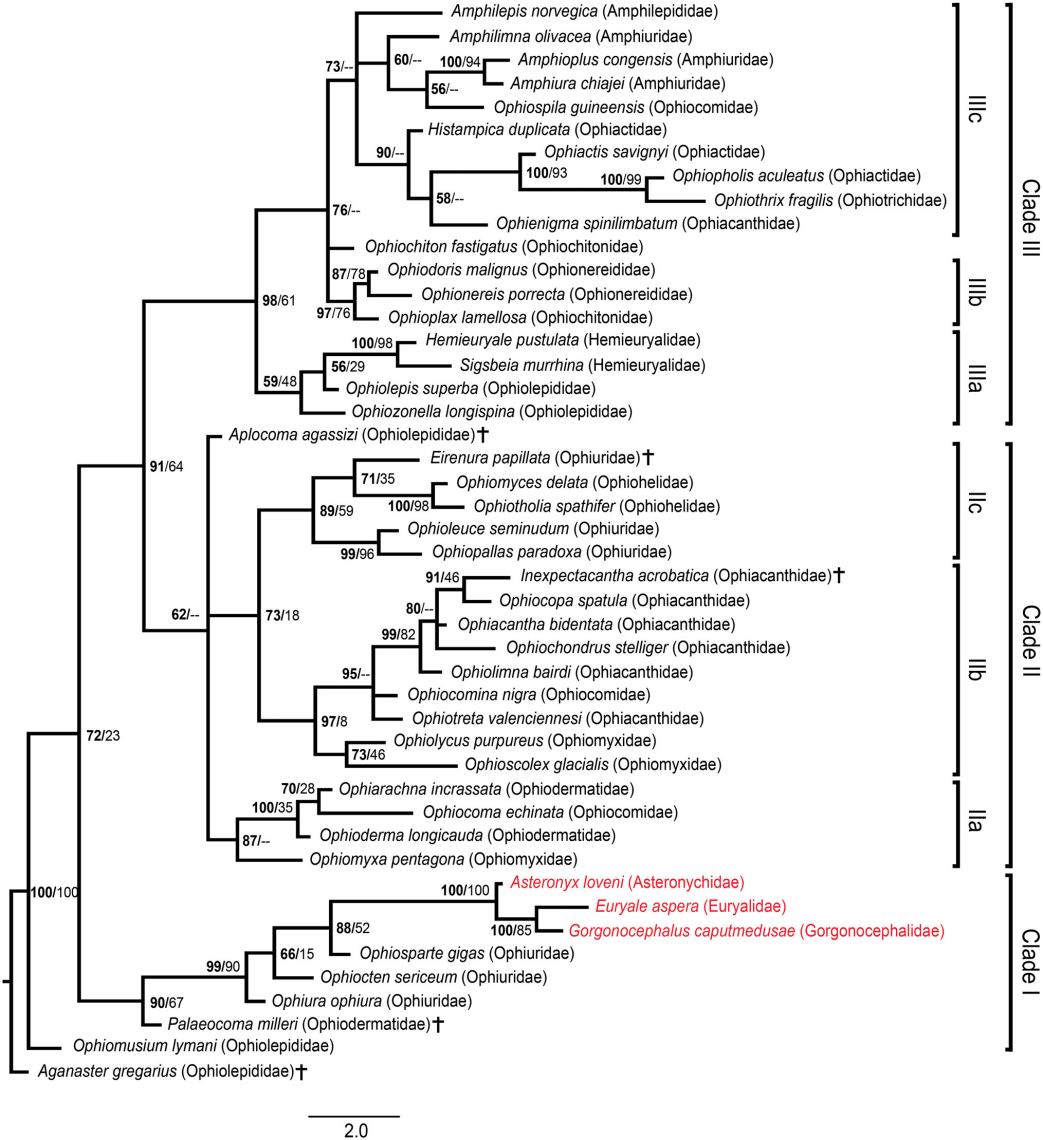
Phylogenetic tree of the full morphological dataset inferred using MrBayes. Numbers at nodes indicate posterior probabilities in bold followed by bootstrap values inferred from parsimony estimates using TNT. For nodes which were not recovered by the parsimony estimate, bootstrap values were omitted (shown as “-“). Euryalids are marked in red, extinct species are marked by a cross.

The tree resulting from Bayesian inference of the full character matrix (Fig 1) shows a high amount of structure with reasonable support, although several relationships remain unresolved. Our analysis favours the subdivision of all ingroup taxa except for *Ophiomusium lymani* into three major clades here termed I, II and III. The first major clade (clade I) gains high support and comprises all members of the former Ophiurinae and Euryalida plus the extinct *Palaeocoma milleri*.

The second major clade II is a rather poorly supported complex of the following groups: 1) clade IIa uniting all sampled ophiodermatids and the ophiomyxid and ophiocomid type taxa, 2) clade IIb gaining high support and comprising the ophiocomid *Ophiocomina nigra* in an unresolved relationship with *Ophiotreta valenciennesi* and all sampled ophiacanthids, and sister to the ophiomyxids *Ophiolycus purpureus* and *Ophioscolex glacialis*, 3) clade IIc a well-supported ophioleucin clade sister to a robust ophiohelid clade including extinct *Eirenura papillata* at its base. Clade IIa and the complex IIb and IIc share an unresolved relationship with the extinct *Aplocoma agassizi*. Finally a well-supported clade III comprising clade IIIa uniting *Ophiolepis superba*, *Ophiozonella longispina* and the hemieuryalids, clade IIIb consisting of the ophionereidids and *Ophioplax lamellosa* in an unresolved relationship with *Ophiochiton fastigatus* and clade IIIc containing all amphiurid, ophiactid, ophiotrichid, and amphilepidid taxa sampled, also including *Ophiopsila guineensis* and *Ophienigma spinilimbatum*. Within clade IIIc, the grouping of the ophiactids and ophiotrichids with *Ophienigma spinilimbatum*, is noteworthy.

**Fig 2:**
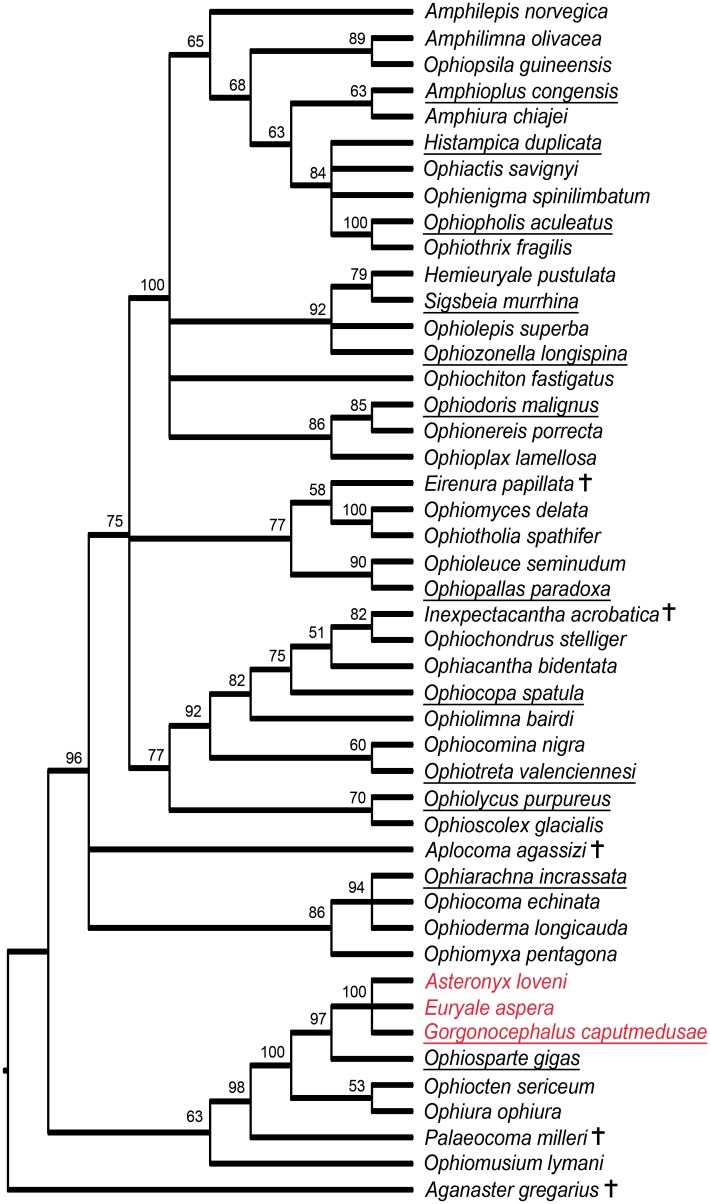
Phylogenetic tree with 13 ingroup taxa reduced to the LAP characters only. Numbers at nodes indicate posterior probabilities. Euryalids are marked in red, extinct taxa are marked by a cross, taxa treated as LAP-only records are underlined.

The tree with 13 ingroup taxa reduced to the LAP characters (Fig 2) shows essentially the same results as the full dataset tree. In particular, the 13 taxa fell within the same clades, albeit with lower support and in some cases a lower resolution. Interestingly, in spite of the drastically reduced amount of information for 13 out of 44 ingroup taxa, the overall support and resolution of the tree was only slightly lower than with the full dataset.

## Discussion

Throughout the process of exploring, testing and optimising our dataset, topology and support values evolved considerably but some key traits of the final tree structure consistently appeared from the beginning. Ophiurids and euryalids consistently formed a clade sister to most other ingroup taxa from the first explorative analyses, irrespective of the analytical approach employed. The position of *Ophiomusium lymani* and *Palaeocoma milleri*, however, proved more variable, albeit always near the base of the ophiurid-euryalid clade.

Clades IIa and IIb were recovered throughout, irrespective of the analytical approaches employed. Also, the ophioleucins and the ophiohelids faithfully formed sister clades, with *Eirenura papillata* mostly at the base of the latter. And finally, clades IIIb and IIIc appeared consistently but with variable internal resolution and topology.

Thus, all groupings gaining high posterior probabilities in the final tree topology seem to stand above methodological differences as well as smaller variations in character definitions and scoring. We therefore consider the clades in question as robust.

The choice of *Aganaster gregarius* as outgroup taxon admittedly bears the risk of excessive proximity to the ingroup taxa, given its superficial similarity with modern ophiuroids [22,33]. However, since the divergence of crown-group ophiuroids was recently dated to the mid-Permian [13], a Lower Carboniferous modern-type ophiuroid is likely to meet the outgroup criterion of having branched from the parent group before the other groups branched from each other. This is corroborated by explorative parsimony analyses performed with no outgroup defined, in which *A*. *gregarius* invariably held the basalmost position in the tree.

### Comparison with previous classifications

Our tree contrasts fundamentally with previous morphology-based formal classifications of the Ophiuroidea (e.g. [2,5,9]). Several traditional family concepts are challenged by our tree, in particular the Ophiomyxidae, Ophiocomidae and the Ophiolepididae which all turn out to be polyphyletic. The Ophiactidae and Ophiochitonidae appear to be paraphyletic by exclusion of the Ophiotrichidae and the Ophionereididae, respectively. It furthermore refutes the previously assumed close relationship between the Ophiurinae and Ophioleucinae and between the Ophiocominae and Ophiopsilinae as formerly expressed by their subfamily status within the Ophiuridae and Ophiocomidae, respectively.

On a higher systematic level, the most striking contrast with previous classifications is that our tree refutes the long-held deep dichotomy between euryalids and all other ophiuroids in favour of an ophiurid-euryalid clade sister to all other ophiuroids except for the former ophiolepidid *Ophiomusium* (e.g. [2,5,9]). With respect to the taxa above family rank in the classification scheme proposed by Smith *et al*. [9], the infraorder Chilophiurina Matsumoto, 1915 is polyphyletic, with the sampled members of the Ophiurinae and the Ophioleucinae falling in completely different clades. The same holds true for the superfamily Ophiocomidea Ljungman, 1867 and the infraorder Ophiodermatina Smith, Paterson & Lafay, 1995. Only the superfamily Gnathophiuridea Matsumoto, 1915 gains support by our analyses, with the Ophiactidae, Ophiotrichidae and Amphiuridae grouped in the same large clade, although the relationship between the Amphiuridae and the other two remains unresolved.

Thus, traditional concepts of ophiuroid classification fail to withstand a cladistic morphological analysis based on species rather than families and thoroughly drawing from the current state of knowledge on ophiuroid morphology. A new classification is overdue but the limited sample size of the present study precludes a formal definition of clades.

### Comparison with molecular evidence

**Fig 3:**
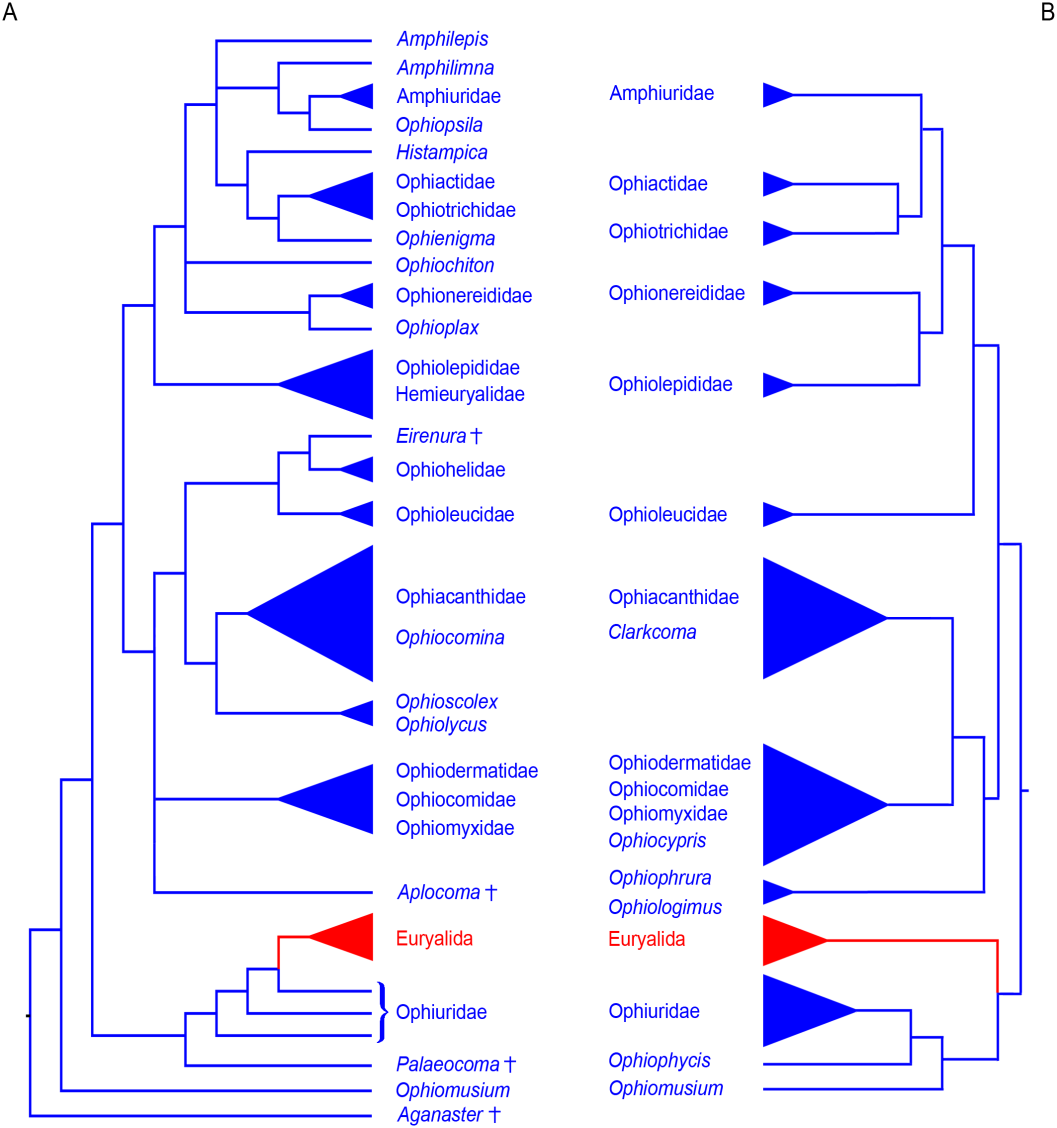
Comparison between the morphology-based phylogenetic tree and the latest phylogenomic tree. (A) Schematic summary of the phylogenetic tree of the full morphological dataset as shown in Fig 1. (B) Schematic summary of the phylogenomic tree redrawn from O’Hara *et al*. [13]. Euryalids are marked in red, extinct taxa are marked by a cross.

Our morphology-based tree compares favourably with the recently published transcriptome-based phylogeny of the Ophiuroidea by O’Hara *et al*. [13] (Fig 3). The analysis in question included 52 ophiuroid species covering 15 of the 18 traditional families. In spite of differences in the set of species sampled (only 4 species represented in both analyses), the recent molecular phylogeny and our novel morphological estimate agree on essential traits of the topology, in particular with respect to previously unrecognized clades.

In fact, the euryalid-ophiurid clade is supported by both estimates, although in our morphological analysis the euryalids lie nested with the ophiurids rather than sharing sister ties with them. Another difference is that our analysis failed to have *Ophiomusium lymani* included within the ophiurid-euryalid clade. Our clades II and III almost exactly correspond to Clades B C of the molecular phylogeny, with the single exception of the ophioleucins falling within clade II instead of III. Even on a lower level, both approaches produce largely convergent topologies, with the ophiodermatid clade including *Ophiomyxa* and *Ophiocoma* supported by both analyses, albeit with differences in internal topology. The ophiacanthid clade appears in both estimates, with morphologically most similar species (*Ophiotreta eximia* (Koehler, 1904) and *Ophiotreta valenciennesi*, *Clarkcoma canaliculata* (Lütken, 1869) and *Ophiocomina nigra*, *Ophiolimna perfida* (Koehler, 1904) and *Ophiolimna bairdi*, *Ophiacantha funebris* (Koehler, 1930) and *Ophiacantha bidentata*, *Ophiomoeris obstricta* (Lyman, 1878) and *Ophiochondrus stelliger*) holding similar positions respectively. Within clade III, ophionereidids have a nested position with respect to the ophiolepidids plus hemieuryalids in our analysis rather than sharing sister ties as suggested by molecular evidence. The topology of our clade IIIc, however, exactly accords with the molecular data.

Thus, in spite of minor differences in some basal branching patterns, there is an astonishing agreement between molecular and our novel morphological evidence. This is all the more remarkable considering that it includes relations such as the euryalid-ophiurid clade and the grouping of *Ophiomyxa* and ophiodermatids, which were never proposed before and might have appeared very unlikely from a traditional morphological point of view. The congruence between our morphological and the latest molecular analyses as independently compiled datasets suggests that efforts in estimating ophiuroid phylogeny are approaching accuracy, i.e., increasingly representing the true phylogeny of the class.

### Lateral arm plate (LAP) evidence

While our new tree contrasts with traditional formal classifications drawing from general skeletal characters, it is in remarkable agreement with recent informal classification schemes proposed by Martynov [17] and Thuy and Stöhr [18] on the basis of novel spine articulation and LAP features. In fact, Martynov [17] already anticipated the close relation between ophiurids and euryalids, and Thuy and Stöhr [18] found that, in terms of LAPs, ophiurids stand apart from other non-euryalids, and *Ophiomusium* shares more similarities with ophiurids than with *Ophiolepis* and relatives. LAP and spine articulation morphologies furthermore challenged the previously assumed sister relationship between ophioleucins and ophiurins, but instead favored close ties between ophiacanthids and *Ophiocomina*, as well as between the ophionereidids, ophiochitonids and *Ophiolepis* [17,18].

Our full dataset analysis thus already corroborates the predicted congruence between LAP morphology patterns and ophiuroid phylogeny [13]. The relevance of LAPs in phylogenetic reconstructions gains further support by the results of our Bayesian inference analysis with 13 of the ingroup taxa reduced to the LAP characters (Fig 2). In fact, our analysis simulated the situation of a combined dataset consisting of taxa known as complete skeletons and taxa known from LAPs only. The congruence with the tree resulting from the full dataset implies that LAP characters are among the most relevant in terms of tree structure. It furthermore suggests that taxa based on dissociated fossil LAPs can be meaningfully included in phylogenetic estimates in combination with fully known taxa. An explorative analysis with all taxa reduced to LAP-characters only proved unsuccessful. Although the resultant tree included some of the clades found in the full dataset analysis, tree support and resolution were too low for to draw meaningful conclusions, possibly as a result of the much lower characters versus taxa ratio (0.93 characters per taxon with the LAP dataset versus 2.89 characters per taxon with the complete dataset) [34].

LAPs occur in almost all types of marine sediments (e.g. [24]), sometimes in great numbers, and are even found in deep-sea sediments which are normally considered devoid of megafaunal remains [21,35]. The results of our combined analysis thus unlock a fossil record of enormous extent, and provide completely new perspectives to assess the evolutionary history of ophiuroids and use them as model organism to explore marine macroevolutionary processes.

### Character analysis and definition of potential synapomorphies

**Fig 4:**
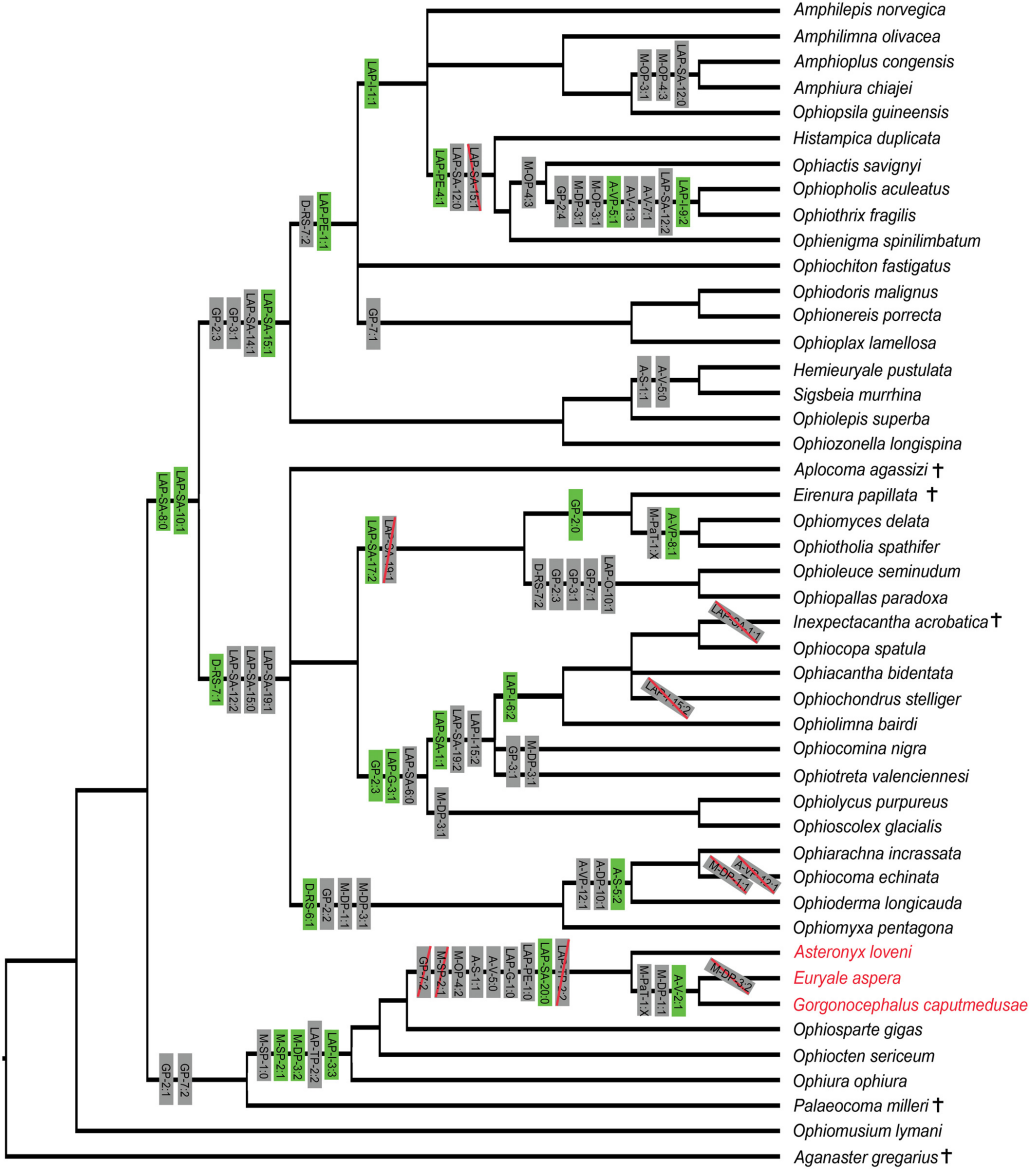
Phylogenetic tree of the full morphological dataset inferred using MrBayes with synapomorphy candidates mapped on the relevant nodes. Character acronyms and state numbers as given in S1 Appendix. Green characters occur only once according to our analysis whereas grey characters occur at least two times independently. Characters applying to single taxa are not shown. Cases of reversals within a particular clade are indicated as character acronym crossed in red. Characters M-DP-5 and M-OP-2 are omitted for the sake of clarity. Euryalids are marked in red, extinct taxa are marked by a cross.

Many characters turned out to show a largely erratic distribution of states across the range of the species sampled herein, with multiple independent acquisitions and reversals. A qualitative character-by-character assessment of our full dataset results allowed the identification of 47 characters showing the most unambiguous correlation between state patterns and tree topology and thus qualifying as the most promising synapomorphy candidates for future formal clade delimitations They are mapped onto the tree in Fig 4, marked by an asterisk in the characters list in S1 Appendix, and explained in further detail as follows:

#### Potential synapomorphies

**Fig 5:**
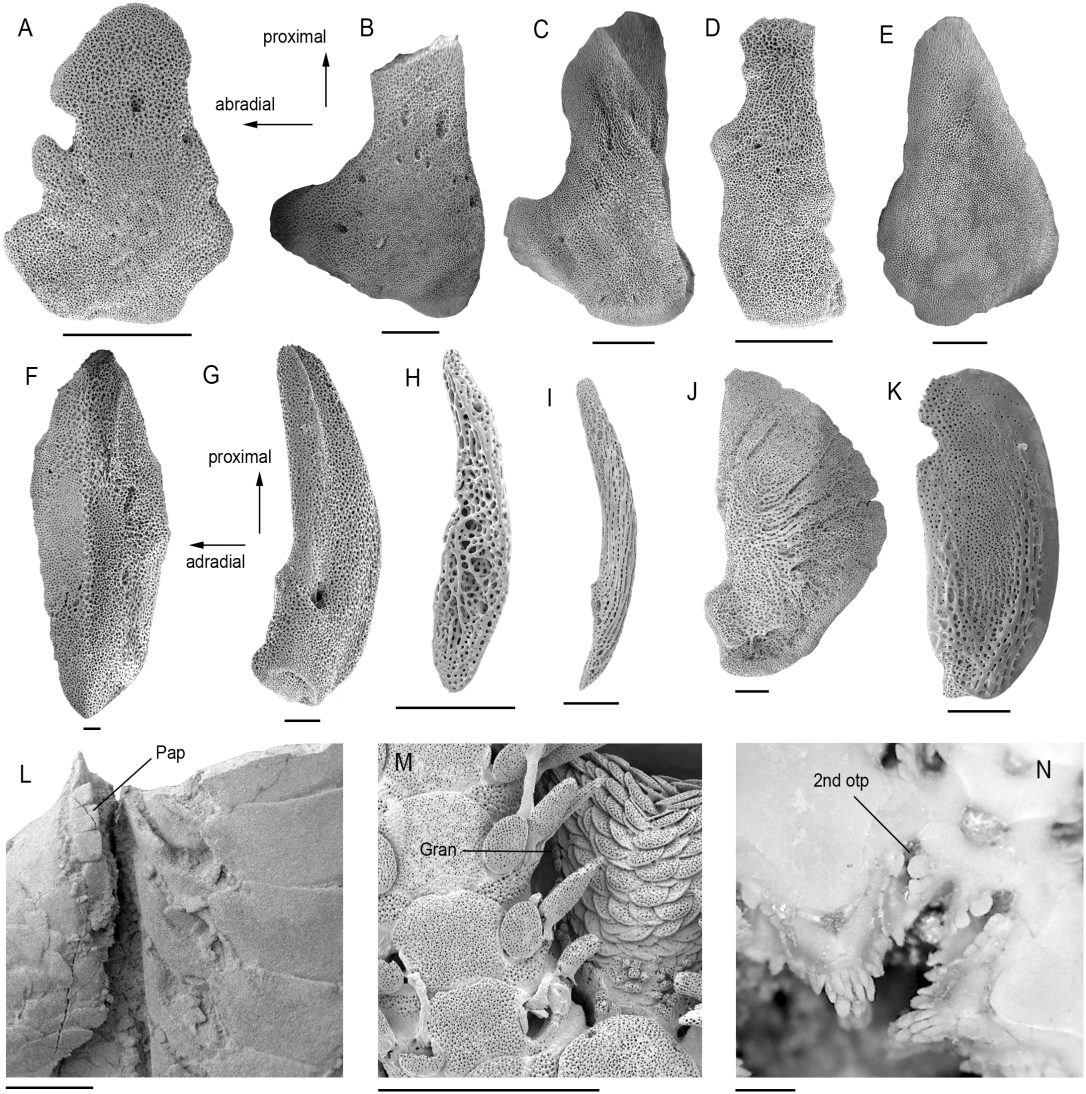
Ophiuroid skeletal characters identified as the potential synapomorphies. (A) Radial shield of *Ophioderma longicauda* (SMNH-133258). (B) Radial shield of *Ophiarachna incrassata* (MnhnL OPH001). (C) Radial shield of *Ophiocoma echinata* (MnhnL OPH002). (D) Radial shield of *Ophiomyxa pentagona* (SMNH-111006). (E) Radial shield of *Ophiura ophiura*, with entire rather than incised abradial edge (MnhnL OPH003). (F) Abradial genital plate of *Ophiomusium lymani* (SMNH-130500). (G) Abradial genital plate of *Ophioderma longicauda* (SMNH-133258). (H) Abradial genital plate of *Ophiolimna bairdi* (SMNH-127044). (I) Abradial genital plate of *Ophiodoris malignus* (MnhnL OPH004). (J) Abradial genital plate of *Ophiothrix fragilis* (SMNH-111046). (K) Abradial genital plate of *Ophiotholia spathifer* (MnhnL OPH005). (L) Detail of genital slit of *Palaeocoma milleri* showing papillae (Pap) on abradial genital plate (MnhnL OPH006). (M) Detail of genital slit of *Ophionereis porrecta* showing granules (Gran) on abradial genital plate (specimen now disarticulated). (N) Detail of ventral disc of *Ophiura ophiura* showing position of second oral tentacle pores (2nd otp) and arrangement of the respective papillae (MnhnL OPH007). Scale bars equal 0.25 mm in (A)-(K) and 1 mm in (L)-(N).

**Fig 6:**
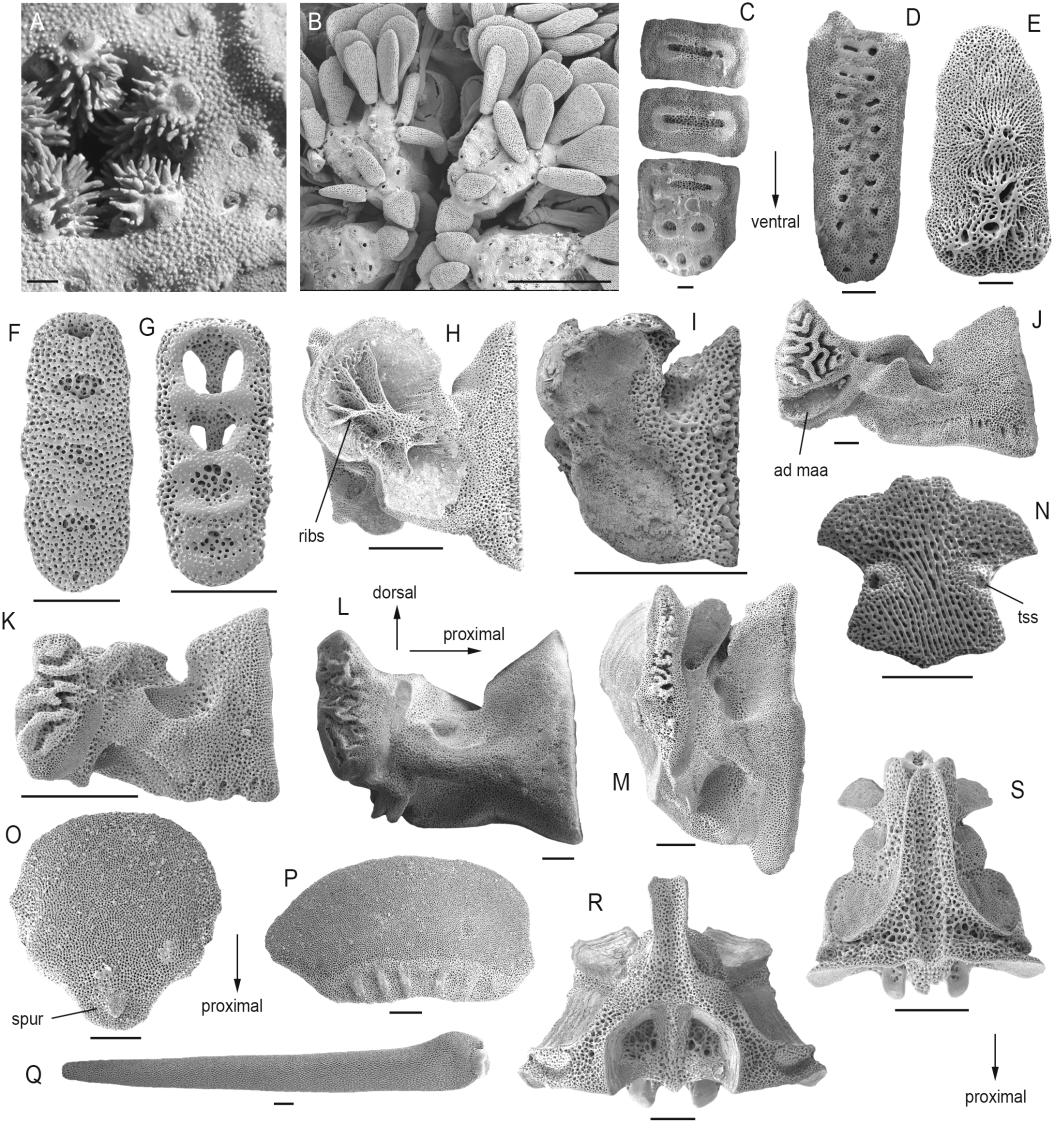
Ophiuroid skeletal characters identified as the potential synapomorphies. (A) Detail of mouth of *Gorgonocepahlus caputmedusae* showing multiple rows of oral papillae (SMNH-131549). (B) Detail of mouth of *Ophiomyces delata* showing multiple rows of oral papillae (SMNH-123450). (C) Dental plate of *Ophiarachna incrassata* (MnhnL OPH008). (D) Dental plate of *Ophiocten sericeum* (MnhnL OPH009). (E) Dental plate of *Ophioscolex glacialis* (SMNH-111001). (F) Dental plate of *Ophiodoris malignus* (MnhnL OPH010). (G) Dental plate of *Ophionereis porrecta* (SMNH-111008). (H) Oral plate of *Amphiura chiajei* in abradial view showing rib-like branching structures (ribs) (SMNH-105100). (I) Oral plate of *Ophiactis savignyi* in abradial view (MnhnL OPH011). (J) Oral plate of *Ophiomusium lymani* in adradial view showing position of adradial muscle attachment area (ad amaa) (SMNH-130500). (K) Oral plate of *Ophiodoris malignus* in adradial view (MnhnL OPH012). (L) Oral plate of *Asteronyx loveni* in adradial view (MnhnL OPH013). (M) Oral plate of *Ophiopholis aculeata* in adradial view (MnhnL OPH014). (N) Ventral arm plate of *Ophiomyces delata* showing sockets for tentacle scales (tss) (MnhnL OPH015). (O) Ventral arm plate of *Ophioderma longicauda* showing spur on proximal edge (spur) (SMNH-133258). (P) Dorsal arm plate of *Ophioderma longicauda* showing spurs on proximal edge (SMNH-133258). (Q) Arm spine of *Ophiarachna incrassata* (MnhnL OPH016). (R) Vertebra of *Ophiothrix fragilis* in dorsal view (SMNH-67805). (S) Vertebra of *Ophiodoris malignus* in dorsal view (MnhnL OPH017). Scale bars equal 1 mm in (A)-(B), 0.25 mm in (C)-(G) and (N)-(S), and 0.5 mm in (H)-(M).

1) Shape of the abradial edge of the radial shields (D-RS-6): All members of clade IIa (ophiodermatid-*Ophiomyxa*-*Ophiocoma* clade) have radial shields with an incised abradial edge (Fig 5A–5D), a previously unnoticed common feature supporting the close ties within the group.

2) Exposure of the radial shields (D-RS-7): All members of clade I (euryalids and ophiurids plus *Palaeocoma*) plus *Ophiomusium* and the outgroup taxon have almost the entire radial shield exposed; in clades IIa (ophiodermatids-*Ophiomyxa*-*Ophiocoma*) and IIb (ophiacanthids-*Ophiocomina*-*Ophioscolex*-*Ophiolycus*), only the distal portion of the radial shields is fully exposed, supporting clade B of O’Hara *et al*. [13]; and the members of clade IIc (ophioleucins-ophiohelids) and clade III have the distal-adradial portion of the radial shields exposed, supporting clade C of O’Hara *et al*. [13], except for clade IIIa (*Ophiolepis*- *Ophiozonella*-hemieuryalids) which have almost the entire radial shield exposed.

3) Shape of the abradial genital plate (GP-2): bar-like with a longitudinal ridge in clade I plus *Ophiomusium* (Fig 5F); bar-like with a longitudinal grove and a large perforation in clade IIa plus *Aplocoma* (Fig 5G); bar-like devoid of conspicuous longitudinal ridges, groves or perforations in clade IIb (Fig 5H), in ophioleucins and in clade III (Fig 5I) except for *Ophiothrix* (Fig 5J), *Ophiopholis* and *Amphilepis* which have a half-ring shaped plate; paddle shaped in ophiohelids plus *Eirenura* (Fig 5K).

4) Shape of the adradio-distal tip of the abradial genital plate (GP-3): all members of clade III have a concave adradio-distal tip of the abradial genital plate (Fig 5I–5K), as well as *Ophiotreta*, *Ophiocomina* and the ophioleucins; in all other sampled species, the tip in question is straight or convex (Fig 5F–5H). This character favours previously unresolved close ties between ophioleucins and members of clade III, supporting Clade C of O’Hara et al. [13]. In *Ophiotreta* and *Ophiocomina*, considering the bulk of evidence against close ties with clade III, the state most probably evolved convergently, and thus sets these two genera apart from other ophiacanthids.

5) Presence of papillae or granules on the abradial genital plate (GP-7): ophiurids plus *Ophiomusium* and *Palaeocoma* all have papillae on the abradial genital plate (genital papillae) (Fig 5L) corroborating their clustering; in the ophioleucins and in clade IIIb (ophionereidids plus *Ophioplax*), the disc granules extend to the edge of the abradial genital plate (Fig 5M), again favouring clade III affinities of the ophioleucins.

6) Position of the second oral tentacle pore (M-SP-1): in the ophiurids and euryalids, and convergently in *Ophioscolex* and *Amphilepis*, the second oral tentacle pore opens outside the mouth slit (Fig 5N); in all others, it opens deep within the mouth slit or at most via a shallow embayment at some distance from the mouth slit. Second oral tentacle pores generally tend to arise outside the mouth slit in juvenile ophiuroids and remain superficial to a variable extent as paedomorphic trait in adults of ophiurids and euryalids as well as a number of non-ophiurid species (e.g. [36]).

7) Presence of extra rows of papillae bordering the second oral tentacle pore and not in line with ordinary lateral oral papillae (M-SP-2): found exclusively in the ophiurids (Fig 5N).

8) Position of the oral papillae on the jaws (M-PaT-1): *Gorgonocephalus* (Fig 6A), *Euryale* and the ophiohelids (Fig 6B) differ from all other ophiuroids in that their jaws are covered in multiple rows of lateral oral papillae, although in the ophiohelids, the rows extend further to the ventral side of the jaws. This difference is difficult to qualify in a cladistics context but it favours an independent origin of the feature in euryalids and ophiohelids respectively.

9) Fragmentation of the dental plate (M-DP-1): unambiguously fragmented dental plates are only found in *Ophiomyxa*, *Ophioderma* and *Ophiarachna* (Fig 6C) on the one hand, corroborating their close ties, and convergently in *Gorgonocephalus* and *Euryale* on the other hand, possibly because of biomechanical constraints related to the considerable height of the jaws in the latter two.

10) Tooth socket patterns on the dental plate (M-DP-3): in clade IIa, in *Ophioscolex* and *Ophiolycus*, in *Ophiocomina* and in *Ophiotreta*, in the pair *Ophiothrix-Ophiopholis*, and in *Palaeocoma*, the ventral portion of the dental plate has multiple rows of tooth sockets (Fig 6C). The ophiurids and the euryalids except for *Euryale* have multiple rows of tooth sockets on the entire dental plate (Fig 6D). Although this character is prone to reversals and convergent acquisition, it proves useful on a lower systematic level, e.g. to set apart *Ophiocomina* and *Ophiotreta* from other ophiacanthids.

11) Shape of the tooth sockets on the dental plate (M-DP-5): *Ophioscolex*, *Ophioleuce*, *Eirenura* and the ophiohelids have simple openings (Fig 6E), either favouring their grouping or representing a symplesiomorphy restricted to basal members of larger clades. In members of the first major clade, in ophiodermatids plus *Ophiomyxa* and *Aplocoma*, in *Ophiolycus* and in *Ophiocomina* and *Ophiotreta*, the tooth sockets are surrounded by a continuous protruding ring (Fig 6C and 6D). All other ophiacanthids, members of clade III and *Ophiocoma* have separate knobs and ridges surrounding the tooth sockets (Fig 6F). A strongly heterogeneous range of species (*Ophiocoma*, *Ophionereis*, *Ophiopholis*, *Ophiothrix*, *Ophiopsila* and the amphiurids) within this group have much more strongly protruding knobs and ridges (Fig 6G), possibly reflecting a yet unknown lifestyle-related independent acquisition.

12) Shape of the abradial muscle fossa of the oral plate (M-OP-2): *Ophiactis*, the *Ophiothrix-Ophiopholis* pair and the amphiurids share a large, well-defined flange (Fig 6H and 6I), which, probably for similar reasons as in the character above, is also found in *Ophiocoma*, *Ophiopsila* and *Ophionereis*.

13) Development of the abradial muscle attachment area of the oral plate (M-OP-3): the *Ophiothrix-Ophiopholis* pair and the amphiurids all show rib-like branching structures (Fig 6H) not found in other ophiuroids.

14) Position of the adradial muscle attachment area of the oral plate (M-OP-4): euryalids stand apart from all other ophiuroids in having the muscle attachment area in middle position, vertical and lining more than two thirds of distal edge of adradial articulation area (Fig 6L). Convergently, this state is shared by *Ophiozonella* and *Amphilimna*. As in previous characters related to the masticatory skeleton, *Ophiactis*, the *Ophiothrix-Ophiopholis* pair and the amphiurids share a particular state (muscle attachment area with a large, dorsal, spoon-shaped depression) (Fig 6M), also found in *Ophiocoma*, *Ophiopsila* and *Ophionereis*.

15) The proximal edge of the ventral arm plates (A-VP-5) is only concave or incised in the *Ophiothrix-Ophiopholis* pair.

16) Sockets for tentacle scales on the lateral edges of the ventral arm plates (A-VP-8) are exclusively found in the ophiohelids (Fig 6N).

17) Spurs on the proximal edge of the ventral arm plates (A-VP-12) are found in the ophiodermatids and, convergently, in *Amphilimna* (Fig 6O).

18) Spurs on the proximal edge of the dorsal arm plate (A-DP-10) are found only in clade IIa and, probably convergently, in *Ophiopallas* and *Ophiozonella* (Fig 6P).

19) Arm spines which are mostly limited to the ventral side of the arm (A-S-1) are a typical feature of the euryalids and, assumably as a result of a convergently epizoic way of life, in the hemieuryalids.

20) The ornamentation of the arm spines (A-S-5) is prone to considerable convergent development but shows one noteworthy pattern: scale-like tubercles are exclusively found in clade IIa (Fig 6Q).

21) Dorso-distal muscular fossae of the vertebra (A-V-1) which are distalwards elongated projecting beyond the zygocondyles of the vertebra or almost so are only found in clades IIIb and IIIc (Fig 6R and 6S), with a true keel (as defined by LeClair [37]) developed in the *Ophiothrix-Ophiopholis* pair and in *Ophionereis* (Fig 6R). In clade IIIa, however, the dorso-distal muscular fossae are not elongated at all, and in the ophiactids and *Amphilepis* only weakly so.

22) The lateral saddle between muscular fossae of the vertebra (A-V-2) shows multiple knobs rather than a single ridge in *Gorgonocephalus* and *Euryale* (Fig 7A).

23) The zygosphene or central peg between two zygocondyles on distal surface of vertebrae (A-V-5) is absent, leading to an hourglass-shaped vertebral articulation, in the euryalids and, probably as a result of a convergently evolved similarly epizoic lifestyle, in hemieuryalids and *Inexpectacantha* (Fig 7B).

24) A large dorsal groove on the proximal side of the vertebrae (A-V-7) corresponding to the dorso-distal keel is only in the *Ophiothrix-Ophiopholis* pair, and in *Ophionereis* (Fig 7C).

25) Lateral arm plates which are essentially lateral rather than wrapped around the arm (LAP-G-1) are found in the euryalids and, convergently, in hemieuryalids, following the same logic as with the position of the arm spines (see above).

26) Constricted proximal LAPs (LAP-G-3) are exclusively found in clade IIb (Fig 7D).

27) LAPs with distalwards pointing scale-like tubercles on their outer surface (LAP-O-10) are only found in the ophioleucins plus *Eirenura* (Fig 7E).

28) Presence of a band of more finely meshed stereom lining the proximal edge of the outer LAP surface (LAP-PE-1): clades IIIb and IIIc are unique in having a band of more finely meshed stereom only in the central part of the outer proximal edge of their LAPs (Fig 7F). Euryalids and some non-euryalids with reduced skeleton and/or thick integument (*Ophiomyxa* and *Ophiolycus*) completely lack the band in question (Fig 7G).

29) Ophiactids plus *Ophienigma* and the *Ophiothrix-Ophiopholis* pair share a protruding central part of the outer proximal edge of their LAPs (LAP-PE-4) (Fig 7H).

30) Spine articulations on an elevated distal portion of the LAPs and bordered by a ridge (LAP-SA-1) are an exclusive feature of the ophiacanthids plus *Ophiocomina* (Fig 7D), except for *Inexpectacantha*.

31) Size pattern of spine articulations (LAP-SA-6): in all members of clade I plus *Ophiomusium* and the outgroup taxon, the spine articulations tend to be of nearly equal size. In members of clade IIb, there is a dorsalward increase in the size of the spine articulations (Fig 7D).

32) The nerve and muscle openings of the spine articulations are separated (LAP-SA-8) by a large, prominent ridge or by a wide stretch of regular stereo in all members of clade I plus *Ophiomusium* (Fig 7I–7K), and separated by a small, non-prominent ridge, if at all, in all other ophiuroids (Fig 7L–7P).

33) The nerve and muscle openings of the spine articulations are encompassed by a dorsal and a ventral ridge (LAP-SA-10) in all ophiuroids (Fig 7L–7P) except for those of clade I plus *Ophiomusium*.

34) Within clade I, non-euryalids stand apart along with *Ophiomusium* in having a vertical, mouth-shaped, sharply defined elevation encompassing the muscle opening (LAP-SA-20) (Fig 7J and 7K).

35) With respect to the orientation of the ridge distally bordering the muscle opening of the spine articulation (LAP-SA-21), *Ophiomusium* is unique in having an oblique ridge (Fig 7K).

36) The dorsal and ventral lobes of the spine articulations are merged proximally (LAP-SA-12) in members of clade II and, convergently, in the *Ophiothrix-Ophiopholis* pair (Fig 7N–7P). In the amphiurids and the ophiactids plus *Ophienigma*, the lobes are simply separated proximally (Fig 7L and 7M), and in all other members of clade III they are separated by at least one knob (Fig 7F).

37) In members of clade III, and convergently in *Ophioscolex*, *Ophiochondrus* and *Ophiotholia*, the dorsal and ventral lobes are parallel (LAP-SA-14) (Fig 7L and 7M).

38) All members of clade III have straight dorsal and ventral lobes (LAP-SA-15) (Fig 7F), except for the ophiactids plus *Ophienigma* and the *Ophiothrix-Ophiopholis* pair which have bent ridges in parallel to members of clade II (Fig 7L–7P).

39) Spine articulations which are nearly vertical (LAP-SA-17) are shared by ophioleucins, *Ophiomyces* and *Eirenura* (Fig 7O). In all members of clade III, the spine articulations are nearly horizontal, except for the *Ophiothrix-Ophiopholis* pair which have oblique spine articulations.

40) The presence of a fully developed sigmoidal fold (LAP-SA-19) as introduced by Martynov (Martynov 2010) unifies all ophiacanthids plus *Ophiocomina* and *Ophiocoma* (Fig 7N). A weakly developed sigmoidal fold can be found in the ophiodermatids plus *Ophiomyxa* and in *Aplocoma* and *Ophiolycus* (Fig 7P).

41) LAPs with tentacle openings which are developed as within-plate perforations from proximal to median arm segments onwards (LAP-TP-1) are found in *Ophiomusium* (Fig 8A).

42) With respect to the orientation of the tentacle notch (LAP-TP-2), the ophiurids stand apart in having a distalwards-pointing tentacle notch positioned close to the midline of the LAP (Fig 8B).

43) The inner side of the LAPs are generally dominated by a continuous vertical ridge (LAP-I-1) except for members of clade IIIc which have two separate round knobs instead (Fig 8C and 8H).

44) The development of the ridge on the inner side of the LAPs (LAP-I-3) unifies the ophiurids and euryalids which have their ridge separated into a ventral and a dorsal half (Fig 8B). In addition, this character sets apart clade IIa and, probably convergently, *Ophiolepis* and *Ophiozonella* which have a main ridge with a separate knob on the ventral tip of the LAP (Fig 8D).

45) With respect to the shape of the ridge on the inner side of the LAPs (LAP-I-6), ophiacanthids minus *Ophiotreta* and *Ophiocomina* stand apart in having a ridge with two kinks and a dorsal tip with a ventro-proximalwards pointing projection (Fig 8E). *Ophiochiton* and clade IIIa and, convergently, *Ophiotreta*, *Ophiocomina* and *Ophiocoma* have a ridge with two kinks and a ventro-proximalwards pointing projection associated with the dorsal kink (Fig 8F and 8G).

46) Among the ophiuroids with two separate knobs on the inner side of the LAPs, the *Ophiothrix-Ophiopholis* pair stands apart in having the knobs associated with a dorsal ridge (LAP-I-9) (Fig 8H).

47) And finally, the ophiacanthids minus *Ophiochondrus* have a vertical row of perforations in a shallow grove on the inner side of the LAPs (LAP-I-15) (Fig 8F), convergently shared with *Ophiomyces*.

**Fig 7:**
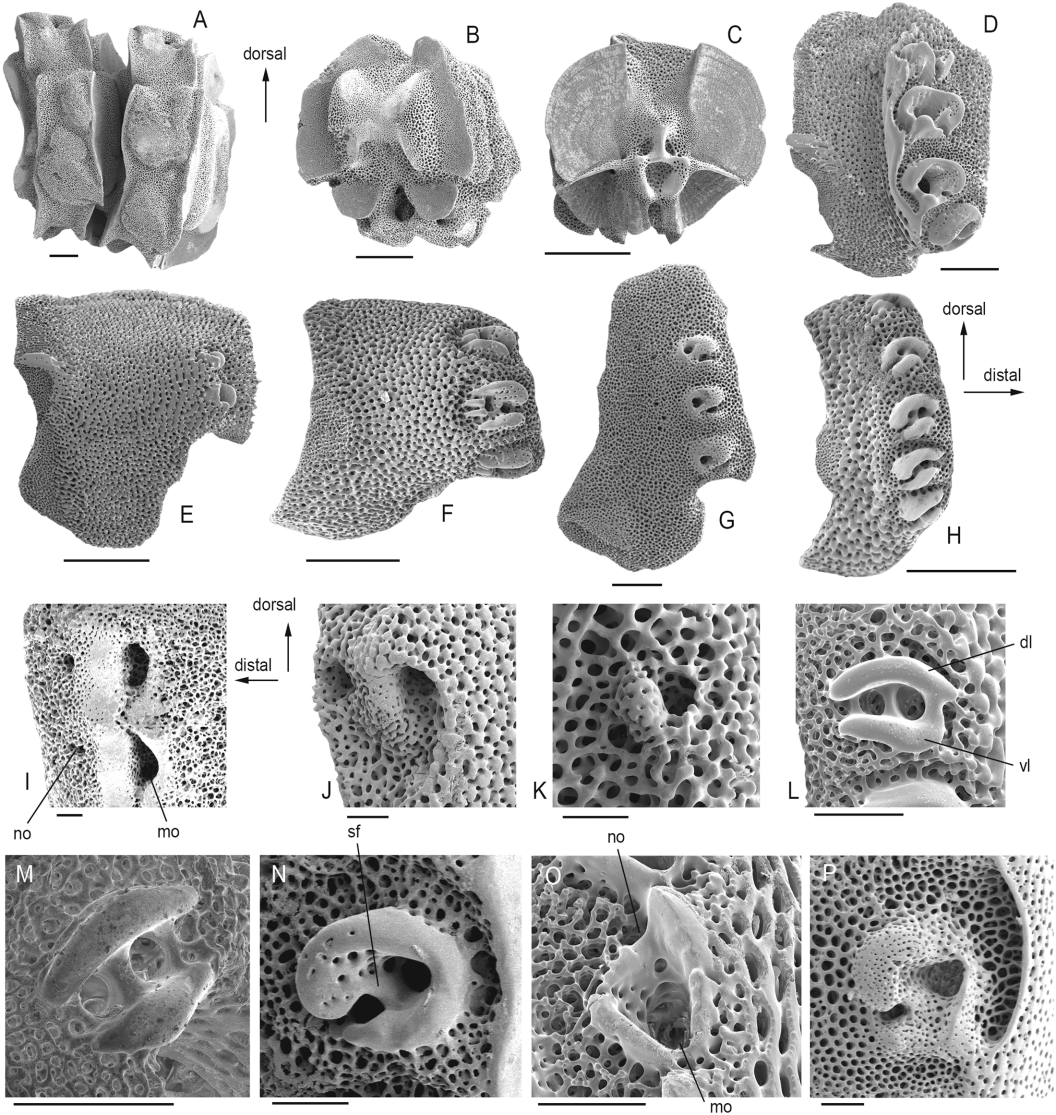
Ophiuroid skeletal characters identified as the potential synapomorphies. (A) Vertebrae of *Gorgonocephalus caputmedusae* in lateral view (SMNH-131549). (B) Vertebra of *Euryale aspera* in distal view (SMNH-131557). (C) Vertebra of *Ophionereis porrecta* in proximal view (SMNH-111047). (D) Lateral arm plate of *Ophiocopa spatula* (SMNH-122912). (E) Lateral arm plate of *Ophioleuce seminudum* (SMNH-118750). (F) Lateral arm plate of *Ophiodoris malignus* (MnhnL OPH018). (G) Lateral arm plate of *Ophiomyxa pentagona* (SMNH-90260). (H) Lateral arm plate of *Ophiactis fragilis* (SMNH-67805). (I) Spine articulations of *Gorgonocephalus caputmedusae* (SMNH-131549). (J) Spine articulation of *Ophiura ophiura* (MnhnL OPH019). (K) Spine articulation of *Ophiomusium lymani* (MnhnL OPH020). (L) Spine articulation of *Ophienigma spinilimbatum* (SMNH-131562). (M) Spine articulation of *Histampica duplicata* (SMNH-131574). (N) Spine articulation of *Ophiacantha bidentata* (SMNH-45856). (O) Spine articulation of *Ophiomyces delata* (SMNH-123451). (P) Spine articulation of *Ophiarachna incrassata* (MnhnL OPH021). Abbreviations: mo: muscle opening; no: nerve opening; dl: dorsal lobe; vl: ventral lobe; sf: sigmoidal fold. All lateral arm plates in external view. Scale bars equal 0.5 in (A)-(C), 0.25 mm in (D)-(H) and 0.1 mm in (I)-(P).

**Fig 8:**
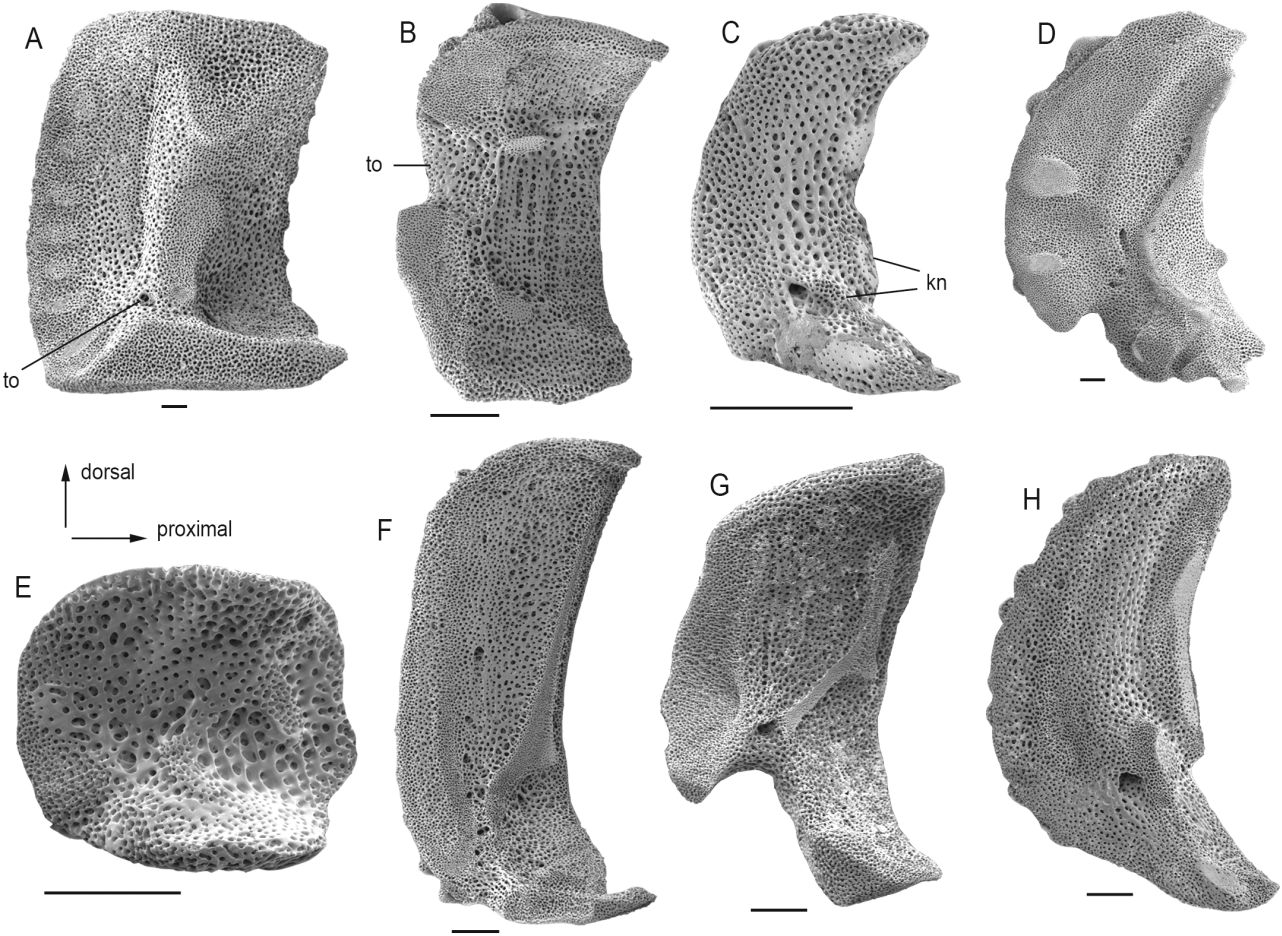
Ophiuroid skeletal characters identified as the potential synapomorphies. (A) Lateral arm plate of *Ophiomusium lymani* showing within-plate tentacle pore (to) (SMNH-130493). (B) Lateral arm plate of *Ophiocten sericeum* showing distalwards-pointing, between-plate tentacle opening (to) (MnhnL OPH022). (C) Lateral arm plate of *Ophiactis savignyi* showing knobs on the inner side (kn) (MnhnL OPH023). (D) Lateral arm plate of *Ophiarachna incrassata* (MnhnL OPH024). (E) Lateral arm plate of *Ophiochondrus stelliger*. (F) Lateral arm plate of *Ophiotreta valenciennesi* (MnhnL OPH026). (G) Lateral arm plate of *Ophiochiton fastigatus* (MnhnL OPH025). (H) Lateral arm plate of *Ophiopholis aculeata* (SMNH-111040). All lateral arm plates in internal view. Scale bars equal 0.25 mm.

The list of potential synapomorphies is mainly meant as an impulse to spark the elaboration of formal clade delimitations. While, in the context of our study, those characters were qualitatively found to perform best in terms of supporting the tree topology, it is important to keep in mind that we only sampled a minute share of present and past ophiuroid diversity. With more species considered, character and state definitions will have to be adapted in order to remain meaningfully applicable.

The list clearly shows that most traditionally used characters fail to support the topology of our new tree. The presence of granules on the disc, for example, formerly used as a key character in ophiuroid classification (e.g. [5]), obviously plays no role at the systematic level investigated herein, although it might still prove useful on species or, in some cases, genus level. Other traditional characters such as the hourglass-shaped vertebral articulation still apply but with restrictions regarding cases of homoplasy which are mostly related to convergently evolved strategies.

Almost half of the characters suggested as potential synapomorphies pertain to the LAPs, which is well in line with O’Hara *et al*. [13] who anticipated the pivotal role of LAPs in ophiuroid phylogeny. While further underpinning the enormous potential of ophiuroid microfossils, this also implies that systematic, SEM-supported studies on microstructures of other skeletal plates carried out with the same rigor as those on the LAPs [17,18] are likely to identify many more phylogenetically relevant characters. The very number of novel LAP and non-LAP characters that were found in the course of this study, most of which rank among the synapomorphy candidates, shows just how far away we are from tapping the full potential of the morphological complexity of the ophiuroid skeleton.

### Implications of the fossil species

Knowledge on the ophiuroid fossil record has dramatically expanded thanks to the inclusion of the data based on dissociated LAPs from micropalaeontological samples (e.g. [26]). Yet, fossil ophiuroids known from material that would allow a morphological assessment at the same level of detail as recent equivalents, i.e. preserved both as pristine skeletons and dissociated skeletal parts, are still very rare (e.g. [38]). As a result, we could only include four fossil species, and our analysis only partly benefitted from the influence of fossils with respect to extant sister groups separated by large morphological gaps (e.g. ophiurids and euryalids), in contrast to echinoids which have a much more extensively studied fossil record [39]. Also, ophiuroid palaeobiodiversity is still too patchily known to conclusively compare our cladogram with the fossil record of first occurrences. Nevertheless, explorative analyses with some or all of the fossil taxa omitted showed that they play a pivotal role with respect to the higher-level branching. Especially *Aplocoma* turned out to be essential in bridging morphological gaps between modern representatives of clades II and III. For the same reason, the fossil taxa had to be treated as fully known in our analysis with a selection of taxa reduced to the LAP characters only.

Our tree confirms the previously suggested ophiacanthid affinities of *Inexpectacantha* [38]. *Eirenura*, previously considered an ophioleucin [38], is here shown to be a probable member of the ophiohelids. Our analysis furthermore refutes the re-assignment of *Aplocoma* to the Ophiolepididae based on similarities with *Ophiozonella* [21] and suggests that the thereupon suggested obsolescence of the family Aplocomidae was premature. Surprisingly, *Palaeocoma* is clearly a basal member of clade I.

The Middle Triassic age and position within our tree of *Aplocoma* implies a crown-group ophiuroid origin by the Early Triassic, which is not in conflict with the mid-Permian crown-group origin recently suggested by O’Hara *et al*. [13]. In order to further explore the early diversification of the crown-group ophiuroids, however, more extensive sampling of Triassic and Jurassic ophiuroids is required. Our conclusions with respect to the phylogenetic relevance of dissociated fossil LAPs might provide highly promising perspectives in this respect.

### Outlook

For future refinement of the morphological analysis, state of the art methods such as geometric morphometrics should be employed for LAPs and other elements. Automatization of character recording should be explored to speed up the process and to limit a possible subjectivity in the assessment of characters by humans. A higher number of characters would allow more species to be included from more genera, maintaing a similar or higher characters versus taxon ratio.

We used exclusively adult characters for this analysis, although studies of juvenile characters have shown promising results that may be highly valuable for phylogenetic inferences [36,40]. However, juvenile stages and skeletal ontogeny are known for only a small number of species so far, which does not include all of our target species. From the fossil record, few juvenile stages are known but show a sgtriking similarity to modern ones (Hess 1960; Hotchkiss 1980). A future study built on species for which juvenile and adult stages are available may provide additional insights into the evolution of Ophiuroidea.

We treated all characters as equal in this analysis regardless of the number of states. It is however possible that characters evolve at different rates. Future models might explore partitioning the data in various ways to explore this. Combining the morphological character matrix with a selection of genetic data is also planned to create a robust phylogeny reconstruction, since it has been shown that molecular datasets can benefit from the addition of morphological data [41,42].

## Supporting Information

S1 Appendix List of characters. (DOCX)

S1 File Score matrix. (HTML)

S2 File Nexus file for TNT. (TNT)

S3 File Nexus file for MrBayes, complete matrix. (NEX)

S4 File Nexus file for MrBayes, 13 recent species reduced to LAP data. (NEX)

S5 File Parsimony execution file for TNT. This is a modified version of the aquickie file included with the TNT package.
(RUN)

S6 File Output of parsimony analysis. (TXT)

S7 File Execution file for bootstrap analysis in TNT. (TXT)

S8 File Output of bootstrap analysis, majority rule. (TXT)

S9 File Output of bootstrap analysis, strict consensus. (TXT)

S1 Table Voucher numbers. Museum catalog numbers of all used specimens.
(DOCX)

## References

[1] Stöhr S, O’Hara TD, Thuy B. Global diversity of brittle stars (Echinodermata: Ophiuroidea). PLoSOne. 2012;7: 1–14. 10.1371/journal.pone.0031940 PMC3292557 22396744

[2] Matsumoto H. A new classification of the Ophiuroidea: with descriptions of new genera and species. Proc Acad Nat Sci Phila. 1915;67: 43–92.

[3] Matsumoto H. A monograph of Japanese Ophiuroidea, arranged according to a new classification. J Coll Sci Imp Univ Tokyo. 1917;38: 1–408.

[4] Mortensen T. Handbook of the Echinoderms of the British Isles. Rotterdam: Backhuys; 1927.

[5] Fell HB. Synoptic keys to the genera of Ophiuroidea. Zool Publ Vic Univ Wellingt. 1960;26: 1–44.

[6] Fell HB. Evidence for the validity of Matsumoto’s classification of the Ophiuroidea. Publ Seto Mar Biol Lab. 1962;10: 145–152.

[7] Fell HB. The phylogeny of sea-stars. Philos Trans R Soc Lond Ser B. 1963;246: 381–435.

[8] Spencer WK, Wright CW. Asterozoans In: Moore RC, editor. Echinodermata 3. 2nd ed Lawrence, Kansas: The Geological Society of North America and Kansas University Press; 1966 pp. U4–U107.

[9] Smith AB, Paterson GLJ, Lafay B. Ophiuroid phylogeny and higher taxonomy: morphological, molecular and palaeontological perspectives. Zool J Linn Soc. 1995;114: 213–243. 10.1111/j.1096-3642.1995.tb00117c.x

[10] Okanishi M, O’Hara TD, Fujita T. Molecular phylogeny of the order Euryalida (Echinodermata: Ophiuroidea), based on mitochondrial and nuclear ribosomal genes. Mol Phylogenet Evol. 2011;61: 392–399. 10.1016/j.ympev.2011.07.003 21798356

[11] Okanishi M, Fujita T. Molecular phylogeny based on increased number of species and genes revealed more robust family-level systematics of the order Euryalida (Echinodermata: Ophiuroidea). Mol Phylogenet Evol. 2013;69: 566–580. 10.1016/j.ympev.2013.07.021 23906601

[12] Stöhr S, O’Hara TD, Thuy B. World Ophiuroidea Database. In: World Ophiuroidea Database [Internet]. 2015 [cited 20 Mar 2015]. Available: http://www.marinespecies.org/ophiuroidea

[13] O’Hara TD, Hugall AF, Thuy B, Moussalli A. Phylogenomic Resolution of the Class Ophiuroidea Unlocks a Global Microfossil Record. Curr Biol. 2014;24: 1874–1879. 10.1016/j.cub.2014.06.060 25065752

[14] Stöhr S. Ophiuroid (Echinodermata) systematics—where do we come from, where do we stand and where should we go? In: Kroh A, Reich M, editors. Echinoderm Research 2010: Proceedings of the Seventh European Conference on Echinoderms, Göttingen, Germany, 2–9 October 2010. 2012. pp. 147–161.

[15] Stöhr S, Segonzac M. Deep-sea ophiuroids (Echinodermata) from reducing and non-reducing environments in the North Atlantic Ocean. J Mar Biol Assoc UK. 2005;85: 383–402. 10.1017/S0025315405011318h

[16] Stöhr S, Segonzac M. Two new genera and species of ophiuroid (Echinodermata) from hydrothermal vents in the East Pacific. Species Divers. 2006;11: 7–32.

[17] Martynov AV. Reassessment of the classification of the Ophiuroidea (Echinodermata), based on morphological characters. I. General character evaluation and delineation of the families Ophiomyxidae and Ophiacanthidae. Zootaxa. 2010;2697: 1–154.

[18] Thuy B, Stöhr S. Lateral arm plate morphology in extant brittle stars (Echinodermata) and its application in micropalaeontology. Zootaxa. 2011;3013: 1–47.

[19] Hunter RL, Brown LM, Alexander Hill C, Kroeger ZA, Rose SE. Additional insights into phylogenetic relationships of the Class Ophiuroidea (Echinodermata) from rRNA gene sequences. J Zool Syst Evol Res. 2016; 10.1111/jzs.12135

[20] Hess H. Trias-Ophiuren aus Deutschland, England, Italien und Spanien. Mitteilungen Bayer Staatssamml Für Paläontol Hist Geol. 1965;5: 151–177.

[21] Thuy B, Gale AS, Kroh A, Kucera M, Numberger-Thuy LD, Reich M, et al Ancient Origin of the Modern Deep-Sea Fauna. PLoS ONE. 2012;7: e46913 10.1371/journal.pone.0046913 23071660 PMC3468611

[22] Hotchkiss FHC, Haude R. Observations on *Aganaster gregarius* and *Stephanoura belgica* (Ophiuroidea: Ophiolepididae) (Early Carboniferous and Late Devonian age). In: Heinzeller T, Nebelsick JH, editors. Echinoderms München Proceedingsa of the 11th International Echinoderm Conference, Munich, Germany, 6–10 October 2003. London: Balkema; 2004. pp. 425–431.

[23] Parameswaran UV, U AJK, Gopal A, N SV, Vijayan AK. On an unusual shallow occurrence of the deep-sea brittle star *Ophiomyces delata* in the Duncan Passage, Andaman Islands (Northern Indian Ocean). Mar Biodivers. 2015; 1–6. 10.1007/s12526-015-0344-6

[24] Hess H. Mikropaläontologische Untersuchungen an Ophiuren: I. Einleitung. Eclogae Geol Helvetiae. 1962;55: 595–608.

[25] Gondim AI, Dias TLP, Christoffersen ML, Stöhr S. Redescription of *Hemieuryale pustulata* von Martens, 1867 (Echinodermata, Ophiuroidea) based on Brazilian specimens, with notes on systematics and habitat association. Zootaxa. 2015;3925: 341–360. 10.11646/zootaxa.3925.3.2 25781748

[26] Thuy B. Temporary expansion to shelf depths rather than an onshore-offshore trend: the shallow-water rise and demise of the modern deep-sea brittle star family Ophiacanthidae (Echinodermata: Ophiuroidea). Eur J Taxon. 2013;0 10.5852/ejt.2013.48

[27] Ung V, Dubus G, Zaragueta-Bagils R, Vignes-Lebbe R. Xper2: introducing e-taxonomy. Bioinformatics. 2010;26: 703–704. 10.1093/bioinformatics/btp715 20053842

[28] Goloboff PA, Farris JS, Nixon KC. TNT, a free program for phylogenetic analysis. Cladistics. 2008;24: 774–786. 10.1111/j.1096-0031.2008.00217.x

[29] Huelsenbeck JP, Ronquist F. MRBAYES: Bayesian inference of phylogenetic trees. Bioinforma Oxf Engl. 2001;17: 754–755. 10.1093/bioinformatics/17.8.754 11524383

[30] Lewis PO. A likelihood approach to estimating phylogeny from discrete morphological character data. Syst Biol. 2001;50: 913–925. 12116640 10.1080/106351501753462876

[31] Evolutionary Models Implemented in MrBayes 3—MbWiki [Internet]. [cited 14 Mar 2016]. Available: http://mrbayes.sourceforge.net/wiki/index.php/Evolutionary_Models_Implemented_in_MrBayes_3#Standard_Discrete_.28Morphology.29_Model

[32] Wright AM, Hillis DM. Bayesian Analysis Using a Simple Likelihood Model Outperforms Parsimony for Estimation of Phylogeny from Discrete Morphological Data. Poon AFY, editor. PLoS ONE. 2014;9: e109210 10.1371/journal.pone.0109210 25279853 PMC4184849

[33] Thuy B, Kutscher M, Płachno B. A new brittle star from the Early Carboniferous of Poland and its implications on Paleozoic modern-type ophiuroid systematics. Acta Palaeontol Pol. 2014; 10.4202/app.00093.2014

[34] Bremer B, Jansen RK, Oxelman B, Backlund M, Lantz H, Kim KJ. More characters or more taxa for a robust phylogeny—case study from the coffee family (Rubiaceae). Syst Biol. 1999;48: 413–435. http://sysbio.oxfordjournals.org/cgi/doi/10.1080/106351599260085 12066290 10.1080/106351599260085

[35] Thuy B, Kiel S, Dulai A, Gale AS, Kroh A, Lord AR, et al First glimpse into Lower Jurassic deep-sea biodiversity: in situ diversification and resilience against extinction. Proc R Soc B Biol Sci. 2014;281: 20132624 10.1098/rspb.2013.2624 PMC4046392 24850917

[36] Stöhr S. Who’s who among baby brittle stars (Echinodermata: Ophiuroidea): postmetamorphic development of some North Atlantic forms. Zool J Linn Soc. 2005;143: 543–576. 10.1111/j.1096-3642.2005.00155.x

[37] LeClair EE. Arm joint articulations in the ophiuran brittlestars (Echinodermata: Ophiuroidea): a morphometric analysis of ontogenetic, serial, and interspecific variation. J Zool Lond. 1996;240: 245–275.

[38] Thuy B. Exceptionally well-preserved brittle stars from the Pliensbachian (Early Jurassic) of the French Ardennes. Palaeontology. 2011;54: 215–233.

[39] Kroh A, Smith AB. The phylogeny and classification of post-Palaeozoic echinoids. J Syst Palaeontol. 2010;8: 147–212. 10.1080/14772011003603556

[40] Sumida PYG, Tyler PA, Gage JD, Nørrevang A. Postlarval development in shallow and deep-sea ophiuroids (Echinodermata: Ophiuroidea) of the NE Atlantic Ocean. Zool J Linn Soc. 1998;124: 267–300. 10.1111/j.1096-3642.1998.tb00577.x

[41] Nylander JAA, Ronquist F, Huelsenbeck JP, Nieves-Aldrey JL. Bayesian phylogenetic analysis of combined data. Syst Biol. 2004;53: 47–67. 14965900 10.1080/10635150490264699

[42] Wortley A, Scotland R. The Effect of Combining Molecular and Morphological Data in Published Phylogenetic Analyses. Syst Biol. 2006;55: 677–685. 10.1080/10635150600899798 16969943

