# Supplementary material for: A New Morphological Phylogeny of the Ophiuroidea (Echinodermata) Accords with Molecular Evidence and Renders Microfossils Accessible for Cladistics
Source: PLoS One. 2016 May 26;11(5):e0156140. doi: 10.1371/journal.pone.0156140 (PMC4882042; doi:10.1371/journal.pone.0156140)
Supplement: S1 Appendix — (DOCX) [file pone.0156140.s001.docx]

**S1 Appendix: List of characters.** The characters marked by an asterisk were selected as potential synapomorphies and used to run an additional analysis.

**D: Dorsal disc**

**D-P: Dorsal disc covering**

D-P-1: Dorsal disc covering: few thin scales (0), many thin scales (1), thick scales (2), very few thin/small scales or none (3).

D-P-3: Dorsal disc scale size (excluding central primary plate and primary radial plates): variable (0), uniform (1).

D-P-4: Dorsal disc scales with smooth tubercles: no (0), yes (1).

D-P-5: Central primary plate relative size: larger than disc scales (0), same size/undistinguishable (1)

D-P-6: Primary radial plates: larger than scales (0), same size/undistinguishable (1).

D-P-7: Primary radial plates position: at a distance from CCP (0), in contact with CCP (1).

D-P-8: Outer integument: thin skin, not obscuring scales/plates (0), thick skin, obscuring scales/plates (1), thick skin with few or no scales (2).

**D-GS: Dorsal disc granules/spines**

D-GS-1: Dorsal disc covering: without granules/spines (0), with granules only (1), with both granules and spines (2), with spines only (3).

D-GS-2: Dorsal disc granules/spines extension: sparse all over with underlying plates/scales visible or restricted to disc margin (0), forming dense cover completely hiding underlying plates/scales (possible exception radial shields) (1).

D-GS-5: Radial shield granule/spine covering: naked (0), at least partly covered (1).

D-GS-6: Dorsal disc granules/spines differentiation: uniform (0), modified (e.g. enlarged) at disc edge (1).

**D-RS: Radial shields**

D-RS-1: present (0), absent (1).

D-RS-3: Radial shields (in articulated disc plating) accounting for: less than one third of the disc radius (0), between one third and half of the disc radius (1), more than half of the disc radius (2).

D-RS-4: Radial shield pairs (in articulated disc plating): completely separated (0), separated distally (1), separated proximally (2), in contact over entire length (3).

D-RS-5: Radial shield shape: scalene (“oblique”) triangular (0), isoscele (“mirror-symmetric”) triangular to pear-shaped (1), "half-circle" (straight inner, convex outer edge, not always as wide as a true half-circle) (2).

D-RS-6: Radial shield abradial edge entire/continuous (0), incised/irregular (1).

D-RS-7: Exposure of RS: central part of RS to almost entire RS exposed (0), distal portion of RS exposed (1), distal-adradial portion of RS exposed (2).

**VI: Ventral interradii and genital slits**

VI-2: Ventral interradii with granules (0), with spines (1), naked (2).

VI-3: Genital slit length: shorter than half interradius (0), longer than half the length of an interradius or divided into two openings (1).

**G: Genital plates**

GP-1: Abradial plate relative length: shorter than half the adradial length (0), longer than half the adradial length (1), as long as adradial one (2).

GP-2: Abradial genital plate shape: paddle-shaped (0), bar-like with longitudinal ridge (1), bar-like with longitudinal grove and large perforation (2), bar-like without ridge or grove (3), half-ring-shaped (4).

GP-3: Shape of adradio-distal tip of abradial genital plate: straight or convex (0), concave (1).

GP-6: Abradial genital plate covering: fully covered (0), exposed (not covered by disc plates/scales) (1).

GP-7: Abradial genital plate bearing papillae/granules: no (0), disc granules (1), papillae (2).

GP-8: Genital papillae/granules extending on latero-distal edge of abradial genital plate?: no (0), yes (1).

GP-9: Shape of ventral genital papillae: granule-like (0), spine-like (1), block-like (2)

**M: Mouth plating**

**M- OAS: Oral shield and adoral shields**

M- OAS-1: Oral shield length: covering less than one third of interradius (0), longer than one third of the length of an interradius (distance between proximal tip of oral shield and disc margin) (1).

M- OAS-2: Oral shield shape: longer than wide (0), as long as wide (1), wider than long (2).

M- OAS-3: Madreporite size: similar to other oral shields (0), larger than remaining oral shields (1).

M- OAS-6: Adoral shields meeting in front of oral shield: no, separated (0), yes (1).

M-OAS-8: Oral shield proximal portion shape: evenly convex (0), obtuse angle with straight to convex sides (1), obtuse angle with concave sides (2), acute to right angle with straight to convex sides (3), acute to right angle with concave sides (4).

M-OAS-9: Oral shield distal portion shape: evenly convex (0), square shaped (1), with narrower distalward projection (2).

**M-SP: Second oral tentacle pore and first ventral arm plate**

M-SP-1: Second oral tentacle pore position: opening completely outside the mouth slit (0), entering mouth slit via shallow embayment or opening deep within the mouth slit (1).

M-SP-2: Second oral tentacle pore covered by extra rows of papillae not in line with ordinary lateral papillae: no (0), yes (1)

**M-PaT: Oral papillae**

M-PaT-1: Lateral papillae position: single row along jaw edge (0), multiple rows covering jaws (1), none (2).

M-PaT-2: Apical papilla (homol. ventralmost tooth): single (0), several (1), tooth papillae (2).

M-PaT-3: Lateral papillae homologous to buccal scale or in place of it: (fragmented into) several papillae (0), typical buccal scale, pointed wide, higher on oral plate (1), single wide papilla (2).

M-PaT-4: MP 2 position (arises laterally on dental plate): lateral (0), infradental (1), absent (2).

M-PaT-5: ASS shape: spiniform (0), scale-like or like other papillae (1), scale-like, much larger/wider than other papillae (2).

M-PaT-6: Additional papillae at 2nd tentacle pore: only at AS (0), also at 1st VAP (1), none/only ASS (2).

M-PaT-7: Lateral papillae shape (excluding higher buccal scale): block-shaped (0), rounded (1), paddle-shaped (2) spiniform (3).

M-PaT-8: Teeth: spine-shaped (0), with round or slightly pointed tip (but never spine-like) (1), with square tip (2).

**M-DP: Dental plate**

M-DP-1: Dental plate: entire (0), fragmented (1).

M-DP-2: Dental plate geometry: equal width all over (0), ventral half widest (1), dorsal half widest (2).

M-DP-3: Pattern of tooth sockets on dental plate: single column throughout (0), multiple columns or cluster on max half of plate length (1), multiple columns throughout (2).

M-DP-5: Predominant shape of tooth sockets on dental plate: surrounded by a more or less continuous, protruding ring (0); simple opening (1), surrounded by separate, weakly protruding knobs and/or ridges (2), surrounded by strongly protruding knobs and/or ridges (3).

M-DP-7: Tooth socket depth: depression or perforating DP without septum (0), at least some perforating DP with septum (1).

**M-OP: Oral plates**

M-OP-1: Oral plate shape: as high as long or higher (0), longer than high (1)

M-OP-2: Abradial muscle fossa: large, well defined flange (0), central depression (1).

M-OP-3: Abradial muscle attachment area with: normal stereom (0), rib-like branching structures (1).

M-OP-4: Adradial muscle attachment area: ventral, lining ventral or ventro-distal edge of articulation area (0), in middle position, vertical and lining less than two thirds of distal edge of adradial articulation area (1), in middle position, vertical and lining more than two thirds of distal edge of adradial articulation area (2), with large, dorsal, spoon-shaped depression (3)

**A: Arms**

**A-G: General aspects**

A-G-2: Integument of arms: naked (0), bearing granules (1).

A-G-3: Arms: simple (0), branched (1).

**A-VP: Ventral arm plates (VAPs)**

A-VP-3: Proximal VAPs (in articulated plating): separated by lateral arm plates (0), potentially in contact (1).

A-VP-4: Distal edge of proximal VAPs (in macerated plate): convex to straight (0), concave or incised (1).

A-VP-5: Proximal edge of proximal VAPs (macerated plate): convex to straight (0), concave or incised (1).

A-VP-6: Distal portion of proximal VAPs (in macerated plate): as wide as proximal portion or narrower (0), wider than proximal portion (1).

A-VP-7: Lateral edge of proximalmost VAPs (in macerated plate) with clear incisions/notches for tentacle openings: no (0), yes (1).

A-VP-8: Edge of tentacle notches with sockets/articulations for tentacle scales (e.g. *Ophiomyces*): no (0), yes (1).

A-VP-10: Proximal VAPs: without conspicuous ornamentation (0), with tubercles or striation (1)

A-VP-12: proximal edge of ventral arm plates (macerated) with spurs: no (0), yes (e.g. *Ophioderma*) (1).

**A-DP: Dorsal arm plates (DAPs)**

A-DP-2: Number of DAPs per segment: single (0), multiple (1), none (2).

A-DP-3: Proximal DAPs: separated (0), in contact (1).

A-DP-4: Shape of proximal DAPs: fan-shaped (0), trapezoid (proximally capped fan) with smooth proximal edge (1), oval semi-circular (2)

A-DP-8: Proximal DAPs: without conspicuous ornamentation (0), tuberculous (1), with striation (2).

A-DP-10: Proximal edge of DAP (macerated) with spurs: no (0), yes (1).

**A-S: Arm spines**

A-S-1: Arm spine position: mainly lateral (0), at proximal segments only on ventral side of arms (e.g. *Asteronyx*): (1).

A-S-2: Arm spines: predominantly parallel to arm axis (adpressed) (0), predominantly erect, standing perpendicular to arm axis (1).

A-S-3: Length of the longest arm spines: shorter than half a segment (0), between half a segment and one segment (1), between one and two segments (2), longer than two segments (3).

A-S-4: Arm spine interior: massive (0), with lumen (1).

A-S-5: Arm spine surface: smooth (0), with lateral thorns (1), with scale-like tubercles (2).

A-S-7: Arm spines cross section: round (0), laterally flattened (1).

A-S-8: Tip of arm spines: blunt (0), pointed (1).

A-S-10: Hook-shaped arm spines: absent (0), only at distal segments (1), at proximal to distal segments (2).

A-S-11: Hook-shaped arm spines: regular arm spines with bent tip and/or saw-toothed edge (0), true, hyaline hook (1), both (2).

A-S-13: Size pattern of spines: ventralmost spine(s) longest (0), median spine(s) longest (1), dorsalmost spine(s) longest (2), all equal (3).

A-S-14: Number of arm spines at proximal to median arm segments: decreasing distalwards (0), constant (e.g. *Ophiura*) (1).

**A-TS: Tentacle scales (n.b. development of tentacle pores as between-plate or within-plate openings is listed under lateral arm plates section)**

A-TS-1: Tentacle scale: present (0), absent (1).

A-TS-3: Tentacle scale shape: operculiform (nearly as long as wide) (0), leaf-like (slightly longer than wide and blunt) (1), spine-like (more than two times longer than wide and pointed) (2), ventral spine closing tentacle opening (3).

A-TS-4: Tentacle scale size: not accurately closing tentacle pore (0), accurately closing tentacle pore (1).

A-TS-5: Tentacle scale ornamentation: without longitudinal striation (0), with longitudinal striation (1).

A-TS-6: Tentacle scale placement: only at LAP (0), at both LAP and VAP (1)

**A-V: Vertebrae**

A-V-1: Dorso-distal muscular fossae transformed distalwards projecting: no (0), yes, but far from distal edge of zygocondyles (e.g. *Ophiacantha*) (1), yes, almost projecting beyond zygocondyles (e.g. *Ophiodoris*) (2), yes, clearly projecting beyond zygocondyles (e.g. *Ophiothrix*) (3).

A-V-2: Lateral saddle between muscular fossae: with single ridge (0), with multiple knobs (e.g. *Gorgonocephalus*) (1).

A-V-4: Zygocondyles (two major articular knobs of distal surfaces of vertebrae) in proximal vertebrae: nearly parallel (0), dorsalwards converging (1).

A-V-5: Zygosphene (central peg between two zygocondyles on distal surface of vertebrae): absent (0), present and fused with pair of zygocondyles (1).

A-V-6: Zygosphene fused with pair of zygocondyles: not projecting beyond ventral edge of zygocondyles or projecting beyond ventral edge of zygocondyles with projecting part shorter than zygocondyles (0), projecting beyond ventral edge of zygocondyles with projecting part as long as zygocondyles (1), projecting beyond ventral edge of zygocondyles with projecting part longer than zygocondyles (2).

A-V-7: Proximal side of vertebrae dorsally with large groove corresponding to distalwards projecting dorso-distal muscular fossae of distal side: no (0), yes (e.g. *Ophiothrix*) (1).

**LAP: Lateral arm plates (LAPs)**

**LAP-G: General outline**

LAP-G-1: LAP position: only lateral (0), arched (wrapped around the arm)(1).

LAP-G-3: LAPs with constriction (dorsal and/or ventral edge(s) concave): no (0), yes (1).

LAP-G-4: Ventral portion of LAP projecting ventro-proximalwards: no (0), yes (1).

LAP-G-5: Ventro-distal tip of LAP projecting ventralwards (e.g. *Ophiopallas*): no (0), yes (1).

**LAP-O: Outer surface ornamentation**

LAP-O-2: Outer surface trabecular intersections: not protruding (0), protruding to form only knobs approximately the same size than stereom pores (1), protruding to form knobs larger than stereom pores on most of outer surface of LAP (2), protruding to form knobs larger than stereom pores on small part of outer surface of LAP (3).

LAP-O-8: Outer surface vertical striation: absent (0), formed by merged knobs (1), formed by regular ridges (2).

LAP-O-10: Outer surface stereom transformed into distalwards pointing scale-like structures: no (0), yes (1).

**LAP-PE: Outer proximal edge of LAPs**

LAP-PE-1: Proximal edge of outer LAP surface lined by discernible band of different (e.g. more finely meshed) stereom structure: no (0), yes, but only in central part (1), yes, over most of the proximal edge (2).

LAP-PE-2: Spurs on proximal edge of outer LAP surface other than ventro-proximal one (if present): absent (0), more than two small (shorter than one fourth of LAP width) spurs (1), one or two large (wider than one fourth of the LAP width) (2), one or two small (shorter than one fourth of the LAP width) (3) .

LAP-PE-3: Oblique, elongated spur on ventro-proximal tip of outer surface of LAP: absent (0), present (1).

LAP-PE-4: Central part of proximal outer surface edge of LAP: not protruding (0), protruding (1).

LAP-PE-8: Proximal edge of outer LAP surface with horizontal striation: no (0), yes but restricted to small area (e.g. between spurs) (1), yes, along most of the edge (2).

**LAP-SA: Spine articulations**

LAP-SA-1: Spine articulations: on same level as remaining outer surface (0), on elevated portion of LAP bordered proximally by ridge (1), on elevated portion of LAP not bordered proximally by ridge (2), in notches of distal LAP edge (3).

LAP-SA-4: Spine articulations separated from distal edge by the usual outer surface stereom (0), separated from the distal edge by a thin projection of the distal LAP portion (e.g. *Ophiomyces*) (1), directly adjacent to the distal edge of the LAP (2).

LAP-SA-5: Spine articulations restricted to (the ventral or central) portion of the distal edge (0), arranged over entire distal LAP edge (1).

LAP-SA-6: Spine articulation size: dorsalwards increasing in size (0), ventralwards increasing in size (1), middle spine articulation(s) larger (2), all similar (3).

LAP-SA-7: Distance between spine articulations: dorsalwards increasing (0), ventralwards increasing (1), equidistant (2), only 2 (3).

LAP-SA-8: Nerve and muscle openings separated: by small ridge if at all (0), by large, prominent ridge or regular stereom (1).

LAP-SA-9: Nerve opening: smaller than muscle opening (0), approximately as large as muscle opening (1).

LAP-SA-10: dorsal and ventral lobes: absent (e.g. *Ophiura*) (0), present (1).

LAP-SA-11: when dorsal and ventral lobes absent, proximal edge of muscle opening denticulate: no (0), yes (1).

LAP-SA-12: Dorsal and ventral lobes: simply separated (0), separated by one or several knobs or by denticulate stereom (1), merged at their proximal tips by smooth connection (2).

LAP-SA-13: Dorsal and ventral lobe size: one lobe clearly larger than the other (0), equal-sized (1).

LAP-SA-14: Dorsal and ventral lobes parallel: no, shifted (e.g. *Ophiacantha*) (0), yes (e.g. *Amphiura*) (1).

LAP-SA-15: Dorsal and ventral lobes straight: no, at least one lobe bent (0), yes (1).

LAP-SA-16: Dorsal and ventral lobes stereom: with perforations (0), massive (1).

LAP-SA-17: Lobes orientation: nearly horizontal (0), tilted (1), nearly vertical (2).

LAP-SA-19: Sigmoidal fold: absent (0), weakly developed (1), fully developed (2).

LAP-SA-20: When dorsal and ventral lobes absent, muscle opening encompassed by: simple stereom (e.g. *Euryale*), poorly defined circular elevation (e.g. *Asteronyx*) and/or vertical ridge distally and wavy ridge proximally (e.g. *Gorgonocephalus*) (0), vertical mouth-shaped, sharply defined elevation (1).

LAP-SA-21: when dorsal and ventral ridges absent, orientation of ridge distally bordering muscle opening: vertical (0), oblique (1).

LAP-SA-22: when dorsal and ventral ridges absent, shape of ridge distally bordering muscle opening: slender (0), thick, lip-shaped and strongly protruding (1).

**LAP-TP: Tentacle opening**

LAP-TP-1: Tentacle opening developed as: notch beyond the first segments under the disc (0), within-plate perforation beyond the first segments under the disc (1).

LAP-TP-2: Tentacle notch pointing: ventralwards (0), ventro-distalwards (1), distalwards, being positioned close to the horizontal midline of the LAP (2).

LAP-TP-3: Tentacle notch externally lined by narrow groove: no (0), yes (e.g. *Aganaster*, basalmost segments in *Ophiomusium*) (1).

LAP-TP-4: Inner side of tentacle notch with horizontally stretched stereom: no (0), yes (e.g. *Ophioscolex*) (1).

**LAP-I: Inner side of LAP, ridges and knobs**

LAP-I-1: Inner side of LAP dominated by: more or less continuous ridge (0), two separate (rarely merged) central knobs (e.g. *Amphiura*) (1).

LAP-I-3: Ridge on inner side of LAP: entire (0), with ventral tip of ventro-proximalwards pointing part of ridge separated from remaining ridge (1), with separate knob on the ventral tip of the LAP (e.g. *Ophioderma*) (2), ridge separated into two halves (3).

LAP-I-5: Ridge on inner side of LAP composed of: the same stereom as remaining inner surface of LAP (0), more compact or more densely meshed stereom (1).

LAP-I-6: Ridge shape: without major kink and with tongue-shaped dorsal tip (0), with kink between dorso-proximalwards pointing dorsal portion and ventro-proximalwards pointing ventral portion, and with tongue-shaped dorsal tip (1), with two kinks and dorsal tip with ventro-proximalwards pointing projection (2), with two kinks and dorsal kink with ventro-proximalwards pointing projection (3).

LAP-I-9: Two central knobs on inner side of LAP: simple (0), with a knob (1), with a ridge (2).

LAP-I-10: Additional dorsal structure on inner side of LAP merged with proximal one of the two central knobs: no (0), yes (1).

LAP-I-15: Perforations on inner side of LAP discernible: single small or inconspicuous (0), vertical row without furrow (1), vertical row with furrow (2), single large and conspicuous (3).
