# Supplementary material for: A New Morphological Phylogeny of the Ophiuroidea (Echinodermata) Accords with Molecular Evidence and Renders Microfossils Accessible for Cladistics
Source: PLoS One. 2016 May 26;11(5):e0156140. doi: 10.1371/journal.pone.0156140 (PMC4882042; doi:10.1371/journal.pone.0156140)
Supplement: S1 File — (HTML) [file pone.0156140.s002.html]

## Base : New Ophimatrix

|  | [D-P: Dorsal disc covering] 1. D-P-1: Dorsal disc covering | [D-P: Dorsal disc covering] 2. D-P-3: Dorsal disc scale size | [D-P: Dorsal disc covering] 3. D-P-4: Dorsal disc scales with smooth tubercles | [D-P: Dorsal disc covering] 4. D-P-5: Central primary plate relative size | [D-P: Dorsal disc covering] [A-VP: Ventral arm plates] 5. D-P-6: Primary radial plates | [D-P: Dorsal disc covering] 6. D-P-7: Primary radial plates position | [D-P: Dorsal disc covering] 7. D-P-8: Outer integument | [D-GS: Dorsal disc granules/spines] 8. D-GS-1: Dorsal disc covering | [D-GS: Dorsal disc granules/spines] [A-VP: Ventral arm plates] 9. D-GS-2: Dorsal disc granules/spines extension | [D-GS: Dorsal disc granules/spines] 10. D-GS-6: Dorsal disc granules/spines differentiation | [D-RS: Radial shields] 11. D-RS-1: Radial shields | [D-GS: Dorsal disc granules/spines] 12. D-GS-5: Radial shield granule/spine covering | [D-RS: Radial shields] 13. D-RS-3: Radial shields (in articulated disc plating) accounting for | [D-RS: Radial shields] 14. D-RS-4: Radial shield pairs (in articulated disc plating) | [D-RS: Radial shields] 15. D-RS-5: Radial shield shape | [D-RS: Radial shields] 16. D-RS-6: Radial shield abradial edge | [D-RS: Radial shields] 17. D-RS-7: Exposure of RS | [VI: Ventral interradii and genital slits] 18. VI-2: Ventral interradii | [VI: Ventral interradii and genital slits] 19. VI-3: Genital slit length | [GP: Genital plates] 20. GP-1: Abradial plate relative length | [GP: Genital plates] 21. GP-2: Abradial genital plate shape | [GP: Genital plates] 22. GP-3: Shape of adradio-distal tip of abradial genital plate | [GP: Genital plates] 23. GP-6: Abradial genital plate covering | [GP: Genital plates] 24. GP-7: Abradial genital plate bearing papillae/granules | [GP: Genital plates] 25. GP-8: Genital papillae/granules extending on latero-distal edge of abradial genital plate? | [GP: Genital plates] 26. GP-9: Shape of ventral genital papillae | [M-OAS: Oral shield and adoral shields] 27. M-OAS-1: Oral shield length | [M-OAS: Oral shield and adoral shields] 28. M-OAS-2: Oral shield shape | [M-OAS: Oral shield and adoral shields] 29. M-OAS-3: Madreporite size | [M-OAS: Oral shield and adoral shields] 30. M-OAS-6: Adoral shields meeting proximal to oral shield | [M-OAS: Oral shield and adoral shields] 31. M-OAS-8: Oral shield proximal portion shape | [M-OAS: Oral shield and adoral shields] 32. M-OAS-9: Oral shield distal portion shape | [M-P: Second oral tentacle pore and first ventral arm plate] 33. M-SP-1: Second tentacle pore position | [M-P: Second oral tentacle pore and first ventral arm plate] 34. M-SP-2: Second oral tentacle pore covered by extra rows of papillae not in line with ordinary lateral papillae | [M-PaT: Oral papillae] 35. M-Pa-T-1: Lateral papillae number | [M-PaT: Oral papillae] 36. M-PaT-3: Lateral papillae homologous to buccal scale or in place of it | [M-PaT: Oral papillae] 37. M-PaT-4: MP2 position | [M-PaT: Oral papillae] 38. M-PaT-5: ASS shape | [M-PaT: Oral papillae] 39. M-PaT-7: Lateral papillae shape | [M-PaT: Oral papillae] 40. M-Pa-T-2: Apical papilla (homol. ventralmost tooth): | [M-PaT: Oral papillae] 41. M-PaT-6: Additional papillae at 2nd tentacle pore | [M-PaT: Oral papillae] 42. M-PaT-8: Teeth | [M-DP: Dental plate] 43. M-DP-1: Dental plate | [M-DP: Dental plate] 44. M-DP-2: Dental plate geometry | [M-DP: Dental plate] 45. M-DP-3: Pattern of tooth sockets | [M-DP: Dental plate] 46. M-DP-5. Predominant shape of tooth sockets | [M-DP: Dental plate] 47. M-DP-7: Tooth socket depth | [M-OP: Oral plates] 48. M-OP-1: Oral plate shape | [M-OP: Oral plates] 49. M-OP-2: Abradial muscle fossa | [M-OP: Oral plates] 50. M-OP-3: Abradial muscle attachment area with | [M-OP: Oral plates] 51. M-OP-4: Adradial muscle attachment area | [A-G: General aspect of arms] 52. A-G-2: Integument of arms | [A-G: General aspect of arms] 53. A-G-3: arms | [A-VP: Ventral arm plates] 54. A-VP-3: Proximal VAPs (in articulated plating) | [A-VP: Ventral arm plates] 55. A-VP-4: Distal edge of proximal VAPs (in macerated plate) | [A-VP: Ventral arm plates] 56. A-VP-5: Proximal edge of proximal VAPs (macerated) | [A-VP: Ventral arm plates] 57. A-VP-6: Distal portion of proximal VAPs (macerated) | [A-VP: Ventral arm plates] 58. A-VP-7: Lateral edge of proximalmost VAPs (macerated) with clear incisisons/notches for tentacle scale | [A-VP: Ventral arm plates] 59. A-VP-8: Edge of tentacle notches with sockets/articulations for tentacle scales (e.g. Ophiomyces) | [A-VP: Ventral arm plates] 60. A-VP-10: Proximal VAPs | [A-VP: Ventral arm plates] 61. A-VP-12: Proximal edge of VAPs (macerated) with spurs | [A-DP: Dorsal arm plates] 62. A-DP-2: Number of DAPs per segment | [A-DP: Dorsal arm plates] 63. A-DP-3: Proximal DAP series | [A-DP: Dorsal arm plates] 64. A-DP-4: Shape of proximal DAPs | [A-DP: Dorsal arm plates] 65. A-DP-8: Proximal DAP structure | [A-DP: Dorsal arm plates] 66. A-DP-10: Proximal edge of DAP (macerated) with spurs | [A-S Arm spines] 67. A-S-1: Arm spine position | [A-S Arm spines] 68. A-S-2: Arm spines | [A-S Arm spines] 69. A-S-3: Length of longest spine | [A-S Arm spines] 70. A-S-4: Arm spine interior | [A-S Arm spines] 71. A-S-5: Arm spine surface | [A-S Arm spines] 72. A-S-7: Arm spine cross section | [A-S Arm spines] 73. A-S-8: Tip of arm spines | [A-S Arm spines] 74. A-S-10: Hook-shaped arm spines | [A-S Arm spines] 75. A-S-11: Hook-shaped spines shape | [A-S Arm spines] 76. A-S-13: Size pattern of spines | [A-S Arm spines] 77. A-S-14: Number of arm spines at proximal to median arm segments | [A-TS: Tentacle scales] 78. A-TS-1: Tentacle scale | [A-TS: Tentacle scales] 79. A-TS-3: Tentacle scale shape | [A-TS: Tentacle scales] 80. A-TS-4: Tentacle scale size | [A-TS: Tentacle scales] 81. A-TS-5: Tentacle scales ornamentation | [A-TS: Tentacle scales] 82. A-TS-6: Tentacle scale placement | [A-V: Vertebrae] 83. A-V-1: Dorso-distal muscular fossae transformed | [A-V: Vertebrae] 84. A-V-2: Lateral saddle between muscular fossae | [A-V: Vertebrae] 85. A-V-4: Zygocondyles in proximal vertebrae | [A-V: Vertebrae] 86. A-V-5: Zygosphene | [A-V: Vertebrae] 87. A-V-6: Zygosphene fused with pair of zygocondyles | [A-V: Vertebrae] 88. A-V-7: Proximal side of vertebrae dorsally with large groove corresponding to distalwards projecting dorso-distal muscular fossae of distal side | [LAP: Lateral arm plates] 89. LAP-G-1: LAP position | [LAP: Lateral arm plates] 90. LAP-G-3: LAPs with constriction | [LAP: Lateral arm plates] 91. LAP-G-4: Ventral portion of LAP projecting ventro-proximalwards | [LAP: Lateral arm plates] 92. LAP-G-5: Ventro-distal tip of LAP projecting ventralwards (e.g. Ophiopallas) | [LAP: Lateral arm plates] 93. LAP-O-2: Outer trabecular intersections | [LAP: Lateral arm plates] 94. LAP-O-8: Outer surface vertical striation | [LAP: Lateral arm plates] 95. LAP-O-10: Outer surface stereom transformed into distalwards pointing scale-like structures | [LAP: Lateral arm plates] 96. LAP-PE-1: Proximal edge of outer LAP surface lined by discernible band of different (e.g. more finely meshed) stereom structure | [LAP: Lateral arm plates] 97. LAP-PE-2: Spurs on prox. edge of outer LAP surface other than ventro-proximal one (if present): | [LAP: Lateral arm plates] 98. LAP-PE-3: oblique, elongated spur on ventro-proximal tip of outer surface of LAP: | [LAP: Lateral arm plates] 99. LAP-PE-4: Central part of proximal outer surface edge of LAP: | [LAP: Lateral arm plates] 100. LAP-PE-8: Proximal edge of outer LAP surface with horizontal striation | [LAP: Lateral arm plates] 101. LAP-SA-1: Spine articulations placement | [LAP: Lateral arm plates] 102. LAP-SA-4: Spine articulation distance from edge | [LAP: Lateral arm plates] 103. LAP-SA-5: Spine art. series extension | [LAP: Lateral arm plates] 104. LAP-SA-6: Spine art. size | [LAP: Lateral arm plates] 105. LAP-SA-7: Distance between spine art. | [LAP: Lateral arm plates] 106. LAP-SA-8: Nerve and muscle openings separated | [LAP: Lateral arm plates] 107. LAP-SA-9: Nerve opening | [LAP: Lateral arm plates] 108. LAP-SA-10: Dorsal and ventral lobes | [LAP: Lateral arm plates] 109. LAP-SA-11: When dorsal and ventral lobes absent, prox. edge of musc. op. denticulate | [LAP: Lateral arm plates] 110. LAP-SA-20: When dorsal and ventral lobes absent, muscle opening encompassed by | [LAP: Lateral arm plates] 111. LAP-SA-21: When dorsal and ventral ridges absent, orientation of ridge bordering muscle opening | [LAP: Lateral arm plates] 112. LAP-SA-22: when dorsal and ventral ridges absent, shape of ridge distally bordering muscle opening | [LAP: Lateral arm plates] 113. LAP-SA-12: Dorsal and ventral lobes connection | [LAP: Lateral arm plates] 114. LAP-SA-13: Dorsal and ventral lobe size | [LAP: Lateral arm plates] 115. LAP-SA-14: Dorsal and ventral lobes parallel | [LAP: Lateral arm plates] 116. LAP-SA-15: Dorsal and ventral lobes straight | [LAP: Lateral arm plates] 117. LAP-SA-16: Dorsal and ventral lobes stereom | [LAP: Lateral arm plates] 118. LAP-SA-17: Lobes orientation | [LAP: Lateral arm plates] 119. LAP-SA-19: Sigmoidal fold | [LAP: Lateral arm plates] 120. LAP-TP-1: Tentacle opening developed as | [LAP: Lateral arm plates] 121. LAP-TP-2: Tentacle notch pointing | [LAP: Lateral arm plates] 122. LAP-TP-3: Tentacle notch externally lined by narrow groove | [LAP: Lateral arm plates] 123. LAP-TP-4: Inner side of notch with horizontally stretched stereom | [LAP: Lateral arm plates] 124. LAP-I-1: Inner side of LAP dominated by | [LAP: Lateral arm plates] 125. LAP-I-3: Ridge on inner side of LAP | [LAP: Lateral arm plates] 126. LAP-I-5: Ridge composed of | [LAP: Lateral arm plates] 127. LAP-I-6: Ridge shape | [LAP: Lateral arm plates] 128. LAP-I-9: Two central knobs shape | [LAP: Lateral arm plates] 129. LAP-I-10: Additional dorsal structure on inner side of LAP merged with prox. one of two central knobs | [LAP: Lateral arm plates] 130. LAP-I-15: Perforations on inner side of LAP discernible |
| --- | --- | --- | --- | --- | --- | --- | --- | --- | --- | --- | --- | --- | --- | --- | --- | --- | --- | --- | --- | --- | --- | --- | --- | --- | --- | --- | --- | --- | --- | --- | --- | --- | --- | --- | --- | --- | --- | --- | --- | --- | --- | --- | --- | --- | --- | --- | --- | --- | --- | --- | --- | --- | --- | --- | --- | --- | --- | --- | --- | --- | --- | --- | --- | --- | --- | --- | --- | --- | --- | --- | --- | --- | --- | --- | --- | --- | --- | --- | --- | --- | --- | --- | --- | --- | --- | --- | --- | --- | --- | --- | --- | --- | --- | --- | --- | --- | --- | --- | --- | --- | --- | --- | --- | --- | --- | --- | --- | --- | --- | --- | --- | --- | --- | --- | --- | --- | --- | --- | --- | --- | --- | --- | --- | --- | --- | --- | --- | --- | --- | --- |
| 1. Aganaster gregarius | few thin scales | uniform | no | larger than disc scales | larger than scales | at a distance from CPP | ? | without granules/spines | not applicable | not applicable | present | naked | less than one third of the disc radius | completely separated | isoscele triangular to pear-shaped | entire/continuous | central part of RS to almost entire RS exposed | naked | longer than half interradius or divided into two openings | longer than half the adradial plate length | ? | ? | fully covered | no | not applicable | not applicable | covering less than one third of interradius | as long as wide | similar to other oral shields | yes | acute angle with straight to convex sides | with narrower distalward projection | entering mouth slit via shallow embayment or opening deep wihtin mouth slit | no | single row along jaw edge | ? | ? | scale-like or like other papillae | ? | ? | none/only ASS | ? | entire | equal width all over | single column throughout | ? | ? | longer than high | central depression | normal stereom | ventral, lining ventral or ventro-distal edge of articulation area | naked | simple | separated by lateral arm plates | convex to straight | convex to straight | wider than proximal portion | yes | no | without conspicuous ornamentation | no | single | separated | fan-shaped | without conspicuous ornamentation | no | lateral | predominantly parallel to arm axis (adpressed) | shorter than half a segment | ? | smooth | round | blunt | absent | not applicable | median spine(s) longest | decreasing distalwards | present | operculiform | not accurately closing tentacle pore | without longitudinal striation | ? | no | with single ridge | dorsalwards converging | present and fused with pair of zygocondyles | projecting beyond ventral edge of zygoc. with projecting part as long as zygoc. | no | arched | no | yes | yes | protruding to form only knobs approx. the same size as stereom pores | absent | no | yes, over most of the proximal edge | absent | present | not protruding | no | on same level as remaining outer surface | separated from distal edge by the usual outer surface stereom | restricted to ventral or central portion of distal LAP edge | all similar | equidistant | by large, prominent ridge or regular stereom | approx. as large as muscle opening | absent (e.g. Ophiura) | yes | vertical mouth-shaped, sharply defined elevation | oblique | slender | not applicable | not applicable | not applicable | not applicable | not applicable | not applicable | not applicable | within-pore perforation beyond first segments under the disc | ventralwards | yes | no | more or less continuous ridge | entire | more compact or more densely meshed stereom | without major kink and with tongue-shaped dorsal tip | not applicable | not applicable | small or inconspicuous |
| 2. Amphilepis norvegica | many thin scales | uniform | no | larger than disc scales | larger than scales | at a distance from CPP | thin skin, not obscuring plates | without granules/spines | not applicable | not applicable | present | naked | more than half of the disc radius | completely separated | scalene triangular | entire/continuous | distal-adradial portion of RS exposed | naked | longer than half interradius or divided into two openings | longer than half the adradial plate length | half-ring-shaped | concave | fully covered | no | not applicable | not applicable | covering less than one third of interradius | wider than long | larger than remaining oral shields | yes | acute angle with straight to convex sides | with narrower distalward projection | opening completely outside mouth slit | no | single row along jaw edge | single, wide papilla (Amphilepis) | infradental | scale-like, much larger/wider than other papillae | block-shaped | several | none/only ASS | with round or slightly pointed tip (but never spine-like) | entire | equal width all over | single column throughout | surrounded by separate, weakly protruding knobs and/or ridges | depression or perforating DP without septum | longer than high | central depression | normal stereom | ventral, lining ventral or ventro-distal edge of articulation area | naked | simple | separated by lateral arm plates | concave or incised | convex to straight | wider than proximal portion | yes | no | without conspicuous ornamentation | no | single | separated | oval semi-circular | without conspicuous ornamentation | no | lateral | predominantly erect, standing perpendicular to arm | 1-2 segments | massive | smooth | round | pointed | absent | not applicable | median spine(s) longest | decreasing distalwards | absent | not applicable | not applicable | not applicable | not applicable | distalwards projecting far from distal edge of zygocondyles (e.g. Ophiacantha) | with single ridge | dorsalwards converging | present and fused with pair of zygocondyles | projecting beyond ventral edge of zygoc. with projecting part as long as zygoc. | no | arched | no | yes | no | protruding to form only knobs approx. the same size as stereom pores | absent | no | yes, only in central part | absent | absent | not protruding | no | on same level as remaining outer surface | separated from distal edge by a thin projection of the distal LAP portion (e.g. Ophiomyces) | arranged over entire distal LAP edge | ventralwards increasing | dorsalwards increasing | by small ridge if at all | smaller than muscle opening | present | not applicable | not applicable | not applicable | not applicable | separated by one or several knobs or by denticulate stereom | equal-sized | yes (e.g. Amphiura) | yes | massive | nearly horizontal | absent | notch beyond the first segments under the disc | ventro-distalwards | no | no | two separate (rarely merged) central knobs | not applicable | not applicable | not applicable | simple | not applicable | small or inconspicuous |
| 3. Amphilimna olivacea | many thin scales | uniform | no | larger than disc scales | larger than scales | ? | thin skin, not obscuring plates | with spines only | sparse all over with underlying plates/scales visible or restricted to margin | uniform | present | naked | between one third and half of the disc radius | separated distally | half-circle | entire/continuous | distal-adradial portion of RS exposed | with spines | longer than half interradius or divided into two openings | shorter than half the adradial plate length | bar-like without ridge or groove | concave | fully covered | no | not applicable | not applicable | covering less than one third of interradius | as long as wide | larger than remaining oral shields | yes | acute to right angle with convex sides | evenly convex | entering mouth slit via shallow embayment or opening deep wihtin mouth slit | no | single row along jaw edge | (fragmented into) several papillae (Ophiura) | infradental | spiniform | rounded | single | only at AS | with round or slightly pointed tip (but never spine-like) | entire | dorsal half widest | single column throughout | surrounded by separate, weakly protruding knobs and/or ridges | depression or perforating DP without septum | longer than high | central depression | normal stereom | in middle position, vertical and lining more than two thirds of adradial articulation area | naked | simple | potentially in contact | convex to straight | convex to straight | wider than proximal portion | yes | no | without conspicuous ornamentation | yes | single | in contact | oval semi-circular | without conspicuous ornamentation | no | lateral | predominantly erect, standing perpendicular to arm | 1-2 segments | massive | smooth | laterally flattened | pointed | absent | not applicable | ventralmost spine(s) longest | decreasing distalwards | present | spine-like | not accurately closing tentacle pore | with longitudinal striation | at both LAP and VAP | distalwards projecting almost beyond zygocondyles (e.g.. Ophiodoris) | with single ridge | dorsalwards converging | present and fused with pair of zygocondyles | projecting beyond ventral edge of zygoc. with projecting part longer than zygoc. | no | arched | no | yes | yes | protruding to form knobs larger than stereom pores on most of outer surface of LAP | absent | no | yes, only in central part | absent | absent | not protruding | no | on same level as remaining outer surface | directly adjacent to the distal edge of the LAP | arranged over entire distal LAP edge | middle spine art. larger | dorsalwards increasing | by small ridge if at all | approx. as large as muscle opening | present | not applicable | not applicable | not applicable | not applicable | separated by one or several knobs or by denticulate stereom | equal-sized | yes (e.g. Amphiura) | yes | massive | nearly horizontal | absent | notch beyond the first segments under the disc | ventralwards | no | no | two separate (rarely merged) central knobs | not applicable | not applicable | not applicable | simple | not applicable | vertical row without furrow |
| 4. Amphioplus congensis | many thin scales | uniform | no | same size/indistinguishable/absent | same size/indistinguishable/absent | not applicable | thin skin, not obscuring plates | without granules/spines | not applicable | not applicable | present | naked | less than one third of the disc radius | in contact over entire length | half-circle | entire/continuous | distal-adradial portion of RS exposed | naked | longer than half interradius or divided into two openings | ? | ? | ? | ? | no | not applicable | not applicable | covering less than one third of interradius | longer than wide | larger than remaining oral shields | no, separated | acute to right angle with convex sides | with narrower distalward projection | entering mouth slit via shallow embayment or opening deep wihtin mouth slit | no | single row along jaw edge | (fragmented into) several papillae (Ophiura) | infradental | scale-like or like other papillae | block-shaped | single | none/only ASS | with square tip | entire | dorsal half widest | single column throughout | surrounded by strongly protruding knobs and/or ridges | at least some perforating DP with septum | as high as long or higher | large, well defined flange | rib-like branching structures | with large, dorsal, spoon-shaped depression | naked | simple | potentially in contact | concave or incised | convex to straight | wider than proximal portion | no | not applicable | without conspicuous ornamentation | no | single | in contact | fan-shaped | without conspicuous ornamentation | no | lateral | predominantly erect, standing perpendicular to arm | 1-2 segments | with lumen | smooth | round | pointed | absent | not applicable | median spine(s) longest | decreasing distalwards | present | leaf-like | accurately closing tentacle pore | without longitudinal striation | at both LAP and VAP | distalwards projecting almost beyond zygocondyles (e.g.. Ophiodoris) | with single ridge | ? | present and fused with pair of zygocondyles | projecting beyond ventral edge of zygoc. with projecting part longer than zygoc. | no | arched | no | yes | no | protruding to form only knobs approx. the same size as stereom pores | absent | no | yes, only in central part | absent | absent | not protruding | no | on same level as remaining outer surface | directly adjacent to the distal edge of the LAP | arranged over entire distal LAP edge | middle spine art. larger | dorsalwards increasing | by small ridge if at all | approx. as large as muscle opening | present | not applicable | not applicable | not applicable | not applicable | simply separated | equal-sized | yes (e.g. Amphiura) | yes | massive | nearly horizontal | absent | notch beyond the first segments under the disc | ventro-distalwards | no | no | two separate (rarely merged) central knobs | not applicable | not applicable | not applicable | with a knob | yes | single large and conspicuous |
| 5. Amphiura chiajei | many thin scales | uniform | no | larger than disc scales | larger than scales | at a distance from CPP | thin skin, not obscuring plates | without granules/spines | not applicable | not applicable | present | naked | between one third and half of the disc radius | completely separated | half-circle | entire/continuous | distal-adradial portion of RS exposed | naked | longer than half interradius or divided into two openings | as long as adradial plate | bar-like without ridge or groove | concave | fully covered | no | not applicable | not applicable | covering less than one third of interradius | as long as wide | larger than remaining oral shields | yes | obtuse angle with straight to convex sides | with narrower distalward projection | entering mouth slit via shallow embayment or opening deep wihtin mouth slit | no | single row along jaw edge | typical buccal scale, pointed wide, higher on oral plate (Amphiura) | infradental | scale-like or like other papillae | block-shaped | single | none/only ASS | with square tip | entire | dorsal half widest | single column throughout | surrounded by strongly protruding knobs and/or ridges | at least some perforating DP with septum | as high as long or higher | large, well defined flange | rib-like branching structures | with large, dorsal, spoon-shaped depression | naked | simple | potentially in contact | concave or incised | convex to straight | as wide as proximal portion or narrower | no | not applicable | without conspicuous ornamentation | no | single | in contact | fan-shaped | without conspicuous ornamentation | no | lateral | predominantly erect, standing perpendicular to arm | 1-2 segments | with lumen | smooth | round | pointed | absent | not applicable | median spine(s) longest | decreasing distalwards | present | operculiform | not accurately closing tentacle pore | without longitudinal striation | at both LAP and VAP | distalwards projecting almost beyond zygocondyles (e.g.. Ophiodoris) | with single ridge | dorsalwards converging | present and fused with pair of zygocondyles | projecting beyond ventral edge of zygoc. with projecting part longer than zygoc. | no | arched | no | yes | no | protruding to form only knobs approx. the same size as stereom pores | absent | no | yes, only in central part | absent | absent | not protruding | no | on same level as remaining outer surface | directly adjacent to the distal edge of the LAP | arranged over entire distal LAP edge | ventralwards increasing | dorsalwards increasing | by small ridge if at all | approx. as large as muscle opening | present | not applicable | not applicable | not applicable | not applicable | simply separated | equal-sized | yes (e.g. Amphiura) | yes | massive | nearly horizontal | absent | notch beyond the first segments under the disc | ventro-distalwards | no | no | two separate (rarely merged) central knobs | not applicable | not applicable | not applicable | with a knob | yes | single large and conspicuous |
| 6. Aplocoma agassizi | many thin scales | uniform | no | larger than disc scales | same size/indistinguishable/absent | not applicable | ? | with granules only | sparse all over with underlying plates/scales visible or restricted to margin | uniform | present | naked | between one third and half of the disc radius | completely separated | isoscele triangular to pear-shaped | entire/continuous | distal portion of RS exposed | with granules | longer than half interradius or divided into two openings | longer than half the adradial plate length | bar-like with longitudinal groove ad large perforation | straight or convex | fully covered | no | not applicable | not applicable | covering less than one third of interradius | longer than wide | similar to other oral shields | yes | acute angle with straight to convex sides | with narrower distalward projection | entering mouth slit via shallow embayment or opening deep wihtin mouth slit | no | single row along jaw edge | ? | ? | scale-like or like other papillae | ? | single | none/only ASS | spine-shaped | entire | equal width all over | single column throughout | surrounded by a more or less continuous proturding ring | depression or perforating DP without septum | longer than high | central depression | normal stereom | ventral, lining ventral or ventro-distal edge of articulation area | naked | simple | separated by lateral arm plates | convex to straight | convex to straight | wider than proximal portion | yes | no | without conspicuous ornamentation | no | single | in contact | fan-shaped | without conspicuous ornamentation | no | lateral | predominantly parallel to arm axis (adpressed) | between half a segment and one segment | massive | smooth | round | blunt | absent | not applicable | ventralmost spine(s) longest | decreasing distalwards | present | operculiform | accurately closing tentacle pore | without longitudinal striation | only at LAP | no | with single ridge | nearly parallel | present and fused with pair of zygocondyles | not projecting beyond ventral edge of zygoc. or projecting beyond ventral edge of zygoc. with projecting part shorter than zygoc. | no | arched | no | yes | yes | protruding to form only knobs approx. the same size as stereom pores | absent | no | yes, over most of the proximal edge | 1 or 2 small spurs | absent | not protruding | no | on same level as remaining outer surface | separated from distal edge by the usual outer surface stereom | arranged over entire distal LAP edge | ventralwards increasing | equidistant | by small ridge if at all | smaller than muscle opening | present | not applicable | not applicable | not applicable | not applicable | merged at their proximal tips by smooth connection | one lobe clearly larger than the other | no, shifted (e.g. Ophiacantha) | no, at least one lobe bent | with perforations | tilted | weakly developed | notch beyond the first segments under the disc | ventro-distalwards | no | no | more or less continuous ridge | entire | more compact or more densely meshed stereom | without major kink and with tongue-shaped dorsal tip | not applicable | not applicable | vertical row without furrow |
| 7. Asteronyx loveni | very few thin small scales or none | not applicable | not applicable | same size/indistinguishable/absent | same size/indistinguishable/absent | not applicable | thick skin with few or no scales | without granules/spines | not applicable | not applicable | present | naked | more than half of the disc radius | completely separated | isoscele triangular to pear-shaped | entire/continuous | central part of RS to almost entire RS exposed | naked | shorter than half interradius | shorter than half the adradial plate length | bar-like with longitudinal ridge | straight or convex | exposed | no | not applicable | not applicable | covering less than one third of interradius | as long as wide | larger than remaining oral shields | yes | acute angle with straight to convex sides | evenly convex | opening completely outside mouth slit | no | single row along jaw edge | (fragmented into) several papillae (Ophiura) | ? | spiniform | spiniform | several | none/only ASS | spine-shaped | entire | equal width all over | multiple columns throughout | surrounded by a more or less continuous proturding ring | depression or perforating DP without septum | as high as long or higher | central depression | normal stereom | in middle position, vertical and lining more than two thirds of adradial articulation area | naked | simple | potentially in contact | ? | ? | ? | ? | ? | without conspicuous ornamentation | no | none | not applicable | not applicable | not applicable | not applicable | at prox. segments only on ventral side of arm | predominantly erect, standing perpendicular to arm | shorter than half a segment | massive | with lateral thorns | laterally flattened | blunt | at proximal and distal segments | both | dorsalmost spine(s) longest | decreasing distalwards | present | ventral spine closing tentacle opening | not accurately closing tentacle pore | without longitudinal striation | only at LAP | no | with single ridge | nearly parallel | absent | not applicable | no | only lateral | no | no | no | not protruding | absent | no | no | absent | absent | not protruding | no | on same level as remaining outer surface | directly adjacent to the distal edge of the LAP | arranged over entire distal LAP edge | all similar | ventralwards increasing | by large, prominent ridge or regular stereom | smaller than muscle opening | absent (e.g. Ophiura) | no | simple stereom (e.g. Euryale), poorly defined circular elevation (e.g. Asteronyx) and/or vertical ridge distally and wavy ridge prox. (e.g. Gorgonocephalus) | ? | ? | not applicable | not applicable | not applicable | not applicable | not applicable | not applicable | not applicable | notch beyond the first segments under the disc | ventro-distalwards | ? | ? | more or less continuous ridge | ridge separated into two halves | same stereom as remaining inner surface of LAP | without major kink and with tongue-shaped dorsal tip | not applicable | not applicable | vertical row without furrow |
| 8. Eirenura papillata | many thin scales | uniform | no | same size/indistinguishable/absent | ? | ? | thick skin with few or no scales | with both granules and spines | forming dense cover completely hiding underlying plates/scales (possible exception radial shields) | modified (e.g. enlarged) at disc edge | present | at least partly covered | between one third and half of the disc radius | separated proximally | isoscele triangular to pear-shaped | entire/continuous | distal-adradial portion of RS exposed | with granules | longer than half interradius or divided into two openings | ? | paddle-shaped | straight or convex | fully covered | no | not applicable | not applicable | longer than one third of length of interradius | longer than wide | similar to other oral shields | yes | acute to right angle with convex sides | square | entering mouth slit via shallow embayment or opening deep wihtin mouth slit | no | single row along jaw edge | (fragmented into) several papillae (Ophiura) | ? | scale-like or like other papillae | rounded | single | also at 1st VAP | with round or slightly pointed tip (but never spine-like) | entire | equal width all over | single column throughout | simple opening | depression or perforating DP without septum | longer than high | central depression | normal stereom | ventral, lining ventral or ventro-distal edge of articulation area | naked | simple | potentially in contact | concave or incised | convex to straight | wider than proximal portion | yes | no | with tubercles or striation | no | single | separated | trapezoid with smooth proximal edge | without conspicuous ornamentation | no | lateral | predominantly parallel to arm axis (adpressed) | shorter than half a segment | massive | with lateral thorns | laterally flattened | blunt | absent | not applicable | ventralmost spine(s) longest | decreasing distalwards | present | leaf-like | not accurately closing tentacle pore | with longitudinal striation | at both LAP and VAP | distalwards projecting far from distal edge of zygocondyles (e.g. Ophiacantha) | with single ridge | dorsalwards converging | present and fused with pair of zygocondyles | not projecting beyond ventral edge of zygoc. or projecting beyond ventral edge of zygoc. with projecting part shorter than zygoc. | no | arched | no | yes | yes | protruding to form knobs larger than stereom pores on most of outer surface of LAP | formed by merged knobs | yes | yes, over most of the proximal edge | absent | present | not protruding | yes, along most of the edge | on same level as remaining outer surface | separated from distal edge by a thin projection of the distal LAP portion (e.g. Ophiomyces) | arranged over entire distal LAP edge | ventralwards increasing | equidistant | by small ridge if at all | smaller than muscle opening | present | not applicable | not applicable | not applicable | not applicable | merged at their proximal tips by smooth connection | one lobe clearly larger than the other | no, shifted (e.g. Ophiacantha) | no, at least one lobe bent | massive | nearly vertical | absent | notch beyond the first segments under the disc | ventro-distalwards | no | no | more or less continuous ridge | entire | more compact or more densely meshed stereom | with kink between dorso-proximalwards pointing dorsal portion and ventro-proximalwards pointing ventral portion | not applicable | not applicable | vertical row without furrow |
| 9. Euryale aspera | very few thin small scales or none | not applicable | not applicable | same size/indistinguishable/absent | same size/indistinguishable/absent | not applicable | thick skin with few or no scales | with granules only | sparse all over with underlying plates/scales visible or restricted to margin | uniform | present | at least partly covered | more than half of the disc radius | completely separated | isoscele triangular to pear-shaped | entire/continuous | central part of RS to almost entire RS exposed | naked | shorter than half interradius | longer than half the adradial plate length | bar-like with longitudinal ridge | straight or convex | ? | no | not applicable | not applicable | covering less than one third of interradius | as long as wide | larger than remaining oral shields | yes | acute angle with straight to convex sides | evenly convex | opening completely outside mouth slit | no | multiple rows covering jaws | (fragmented into) several papillae (Ophiura) | lateral | ? | rounded | single | none/only ASS | with round or slightly pointed tip (but never spine-like) | fragmented | equal width all over | single column throughout | surrounded by a more or less continuous proturding ring | depression or perforating DP without septum | as high as long or higher | central depression | normal stereom | in middle position, vertical and lining more than two thirds of adradial articulation area | bearing granules | branching | potentially in contact | convex to straight | concave or incised | as wide as proximal portion or narrower | no | not applicable | without conspicuous ornamentation | no | none | not applicable | not applicable | not applicable | not applicable | at prox. segments only on ventral side of arm | predominantly erect, standing perpendicular to arm | shorter than half a segment | ? | with lateral thorns | round | blunt | at proximal and distal segments | true, hyaline hook | ventralmost spine(s) longest | constant | present | ventral spine closing tentacle opening | not accurately closing tentacle pore | without longitudinal striation | only at LAP | no | with multiple knobs (e.g. Gorgonocephalus) | nearly parallel | absent | not applicable | no | only lateral | no | no | no | not protruding | absent | no | no | absent | absent | not protruding | no | on same level as remaining outer surface | directly adjacent to the distal edge of the LAP | restricted to ventral or central portion of distal LAP edge | all similar | only 2 | by large, prominent ridge or regular stereom | approx. as large as muscle opening | absent (e.g. Ophiura) | no | simple stereom (e.g. Euryale), poorly defined circular elevation (e.g. Asteronyx) and/or vertical ridge distally and wavy ridge prox. (e.g. Gorgonocephalus) | ? | ? | not applicable | not applicable | not applicable | not applicable | not applicable | not applicable | not applicable | within-pore perforation beyond first segments under the disc | ventro-distalwards | no | no | more or less continuous ridge | ridge separated into two halves | same stereom as remaining inner surface of LAP | without major kink and with tongue-shaped dorsal tip | not applicable | not applicable | vertical row without furrow |
| 10. Gorgonocephalus caputmedusae | very few thin small scales or none | not applicable | not applicable | same size/indistinguishable/absent | same size/indistinguishable/absent | not applicable | thick skin with few or no scales | with spines only | sparse all over with underlying plates/scales visible or restricted to margin | uniform | present | at least partly covered | more than half of the disc radius | completely separated | isoscele triangular to pear-shaped | entire/continuous | central part of RS to almost entire RS exposed | with spines | shorter than half interradius | ? | bar-like with longitudinal ridge | straight or convex | ? | no | not applicable | not applicable | covering less than one third of interradius | wider than long | larger than remaining oral shields | yes | obtuse angle with straight to convex sides | square | opening completely outside mouth slit | no | multiple rows covering jaws | (fragmented into) several papillae (Ophiura) | ? | spiniform | spiniform | several | only at AS | spine-shaped | fragmented | equal width all over | multiple columns throughout | surrounded by a more or less continuous proturding ring | depression or perforating DP without septum | as high as long or higher | central depression | normal stereom | in middle position, vertical and lining more than two thirds of adradial articulation area | bearing granules | branching | potentially in contact | ? | ? | ? | ? | ? | without conspicuous ornamentation | no | multiple | in contact | oval semi-circular | without conspicuous ornamentation | no | at prox. segments only on ventral side of arm | predominantly erect, standing perpendicular to arm | shorter than half a segment | massive | with lateral thorns | laterally flattened | blunt | at proximal and distal segments | both | ventralmost spine(s) longest | decreasing distalwards | present | ventral spine closing tentacle opening | not accurately closing tentacle pore | without longitudinal striation | only at LAP | no | with multiple knobs (e.g. Gorgonocephalus) | nearly parallel | absent | not applicable | no | only lateral | no | yes | no | not protruding | absent | no | no | absent | absent | not protruding | no | on same level as remaining outer surface | directly adjacent to the distal edge of the LAP | restricted to ventral or central portion of distal LAP edge | all similar | ventralwards increasing | by large, prominent ridge or regular stereom | smaller than muscle opening | absent (e.g. Ophiura) | no | simple stereom (e.g. Euryale), poorly defined circular elevation (e.g. Asteronyx) and/or vertical ridge distally and wavy ridge prox. (e.g. Gorgonocephalus) | vertical | slender | not applicable | not applicable | not applicable | not applicable | not applicable | not applicable | not applicable | notch beyond the first segments under the disc | ventro-distalwards | no | no | more or less continuous ridge | ridge separated into two halves | same stereom as remaining inner surface of LAP | without major kink and with tongue-shaped dorsal tip | not applicable | not applicable | vertical row without furrow |
| 11. Hemieuryale pustulata | thick scales | variable | yes | larger than disc scales | larger than scales | at a distance from CPP | thin skin, not obscuring plates | without granules/spines | not applicable | not applicable | present | naked | more than half of the disc radius | completely separated | isoscele triangular to pear-shaped | entire/continuous | central part of RS to almost entire RS exposed | naked | shorter than half interradius | ? | ? | ? | ? | no | not applicable | not applicable | longer than one third of length of interradius | wider than long | larger than remaining oral shields | yes | obtuse angle with straight to convex sides | evenly convex | entering mouth slit via shallow embayment or opening deep wihtin mouth slit | no | single row along jaw edge | (fragmented into) several papillae (Ophiura) | infradental | scale-like, much larger/wider than other papillae | rounded | single | none/only ASS | with round or slightly pointed tip (but never spine-like) | entire | dorsal half widest | single column throughout | surrounded by separate, weakly protruding knobs and/or ridges | depression or perforating DP without septum | longer than high | central depression | normal stereom | in middle position, vertical and lining less than two thirds of distal edge of adradial articulation area | naked | simple | potentially in contact | convex to straight | convex to straight | wider than proximal portion | yes | no | with tubercles or striation | no | multiple | in contact | ? | without conspicuous ornamentation | no | at prox. segments only on ventral side of arm | predominantly parallel to arm axis (adpressed) | between half a segment and one segment | ? | smooth | round | blunt | absent | not applicable | ventralmost spine(s) longest | decreasing distalwards | present | operculiform | accurately closing tentacle pore | without longitudinal striation | only at LAP | no | with single ridge | nearly parallel | absent | not applicable | no | only lateral | no | yes | no | protruding to form knobs larger than stereom pores on most of outer surface of LAP | absent | no | yes, only in central part | 1 or 2 small spurs | present | not protruding | yes, but restricted to small area (e.g. between spurs) | on same level as remaining outer surface | directly adjacent to the distal edge of the LAP | arranged over entire distal LAP edge | ventralwards increasing | only 2 | by small ridge if at all | smaller than muscle opening | present | not applicable | not applicable | not applicable | not applicable | separated by one or several knobs or by denticulate stereom | equal-sized | yes (e.g. Amphiura) | yes | with perforations | nearly horizontal | absent | notch beyond the first segments under the disc | ventro-distalwards | no | no | more or less continuous ridge | with ventral tip of bentro-proximalwards pointing part of ridge separated from remaining ridge | same stereom as remaining inner surface of LAP | without major kink and with tongue-shaped dorsal tip | not applicable | not applicable | small or inconspicuous |
| 12. Histampica duplicata | many thin scales | variable | no | larger than disc scales | larger than scales | at a distance from CPP | thin skin, not obscuring plates | without granules/spines | not applicable | not applicable | present | naked | between one third and half of the disc radius | completely separated | half-circle | entire/continuous | distal-adradial portion of RS exposed | naked | longer than half interradius or divided into two openings | longer than half the adradial plate length | bar-like without ridge or groove | concave | fully covered | no | not applicable | not applicable | covering less than one third of interradius | as long as wide | larger than remaining oral shields | yes | acute to right angle with convex sides | evenly convex | entering mouth slit via shallow embayment or opening deep wihtin mouth slit | no | single row along jaw edge | (fragmented into) several papillae (Ophiura) | lateral | scale-like or like other papillae | rounded | single | none/only ASS | with round or slightly pointed tip (but never spine-like) | entire | equal width all over | single column throughout | surrounded by separate, weakly protruding knobs and/or ridges | depression or perforating DP without septum | longer than high | central depression | normal stereom | in middle position, vertical and lining less than two thirds of distal edge of adradial articulation area | naked | simple | potentially in contact | convex to straight | convex to straight | wider than proximal portion | yes | no | without conspicuous ornamentation | no | single | separated | fan-shaped | without conspicuous ornamentation | no | lateral | predominantly erect, standing perpendicular to arm | 1-2 segments | with lumen | smooth | round | pointed | absent | not applicable | dorsalmost spine(s) longest | decreasing distalwards | present | operculiform | accurately closing tentacle pore | without longitudinal striation | only at LAP | distalwards projecting far from distal edge of zygocondyles (e.g. Ophiacantha) | with single ridge | dorsalwards converging | present and fused with pair of zygocondyles | not projecting beyond ventral edge of zygoc. or projecting beyond ventral edge of zygoc. with projecting part shorter than zygoc. | no | arched | no | yes | no | protruding to form knobs larger than stereom pores on most of outer surface of LAP | absent | no | yes, only in central part | absent | absent | protruding | yes, but restricted to small area (e.g. between spurs) | on same level as remaining outer surface | directly adjacent to the distal edge of the LAP | arranged over entire distal LAP edge | middle spine art. larger | dorsalwards increasing | by small ridge if at all | approx. as large as muscle opening | present | not applicable | not applicable | not applicable | not applicable | simply separated | one lobe clearly larger than the other | yes (e.g. Amphiura) | no, at least one lobe bent | massive | nearly horizontal | absent | notch beyond the first segments under the disc | ventro-distalwards | no | no | two separate (rarely merged) central knobs | not applicable | not applicable | not applicable | with a knob | no | vertical row without furrow |
| 13. Inexpectacantha acrobatica | few thin scales | uniform | no | larger than disc scales | larger than scales | in contact with CPP | ? | with granules only | sparse all over with underlying plates/scales visible or restricted to margin | uniform | present | at least partly covered | less than one third of the disc radius | completely separated | isoscele triangular to pear-shaped | entire/continuous | distal portion of RS exposed | with granules | longer than half interradius or divided into two openings | longer than half the adradial plate length | bar-like without ridge or groove | straight or convex | fully covered | no | not applicable | not applicable | longer than one third of length of interradius | as long as wide | larger than remaining oral shields | yes | acute angle with straight to convex sides | with narrower distalward projection | entering mouth slit via shallow embayment or opening deep wihtin mouth slit | no | single row along jaw edge | (fragmented into) several papillae (Ophiura) | ? | scale-like or like other papillae | rounded | single | ? | with round or slightly pointed tip (but never spine-like) | entire | equal width all over | single column throughout | surrounded by separate, weakly protruding knobs and/or ridges | depression or perforating DP without septum | longer than high | central depression | normal stereom | ventral, lining ventral or ventro-distal edge of articulation area | naked | simple | separated by lateral arm plates | convex to straight | convex to straight | wider than proximal portion | yes | no | with tubercles or striation | no | single | separated | fan-shaped | tuberculous | no | lateral | predominantly erect, standing perpendicular to arm | longer than 2 segments | ? | with lateral thorns | round | pointed | only at distal segments | true, hyaline hook | dorsalmost spine(s) longest | decreasing distalwards | present | leaf-like | accurately closing tentacle pore | with longitudinal striation | only at LAP | distalwards projecting far from distal edge of zygocondyles (e.g. Ophiacantha) | with single ridge | nearly parallel | absent | not applicable | no | arched | yes | no | no | protruding to form knobs larger than stereom pores on most of outer surface of LAP | absent | no | yes, over most of the proximal edge | 1 or 2 small spurs | absent | not protruding | yes, along most of the edge | on elevation not bordered by ridge | separated from distal edge by the usual outer surface stereom | arranged over entire distal LAP edge | dorsalwards increasing | dorsalwards increasing | by small ridge if at all | smaller than muscle opening | present | not applicable | not applicable | not applicable | not applicable | merged at their proximal tips by smooth connection | one lobe clearly larger than the other | no, shifted (e.g. Ophiacantha) | no, at least one lobe bent | with perforations | tilted | fully developed | notch beyond the first segments under the disc | ventro-distalwards | no | no | more or less continuous ridge | with ventral tip of bentro-proximalwards pointing part of ridge separated from remaining ridge | more compact or more densely meshed stereom | with two kinks and dorsal tip with ventro-proximalwards pointing projection | not applicable | not applicable | vertical row with furrow |
| 14. Ophiacantha bidentata | many thin scales | uniform | no | same size/indistinguishable/absent | same size/indistinguishable/absent | not applicable | thin skin, not obscuring plates | with both granules and spines | forming dense cover completely hiding underlying plates/scales (possible exception radial shields) | uniform | present | at least partly covered | between one third and half of the disc radius | completely separated | isoscele triangular to pear-shaped | entire/continuous | distal portion of RS exposed | with granules | longer than half interradius or divided into two openings | shorter than half the adradial plate length | bar-like without ridge or groove | straight or convex | fully covered | no | not applicable | not applicable | covering less than one third of interradius | wider than long | similar to other oral shields | yes | obtuse angle with concave sides | evenly convex | entering mouth slit via shallow embayment or opening deep wihtin mouth slit | no | single row along jaw edge | (fragmented into) several papillae (Ophiura) | lateral | scale-like, much larger/wider than other papillae | spiniform | single | none/only ASS | with round or slightly pointed tip (but never spine-like) | entire | equal width all over | single column throughout | surrounded by separate, weakly protruding knobs and/or ridges | depression or perforating DP without septum | longer than high | central depression | normal stereom | ventral, lining ventral or ventro-distal edge of articulation area | naked | simple | separated by lateral arm plates | convex to straight | convex to straight | wider than proximal portion | yes | no | with tubercles or striation | no | single | separated | fan-shaped | without conspicuous ornamentation | no | lateral | predominantly erect, standing perpendicular to arm | longer than 2 segments | with lumen | with lateral thorns | round | pointed | absent | not applicable | dorsalmost spine(s) longest | decreasing distalwards | present | operculiform | accurately closing tentacle pore | with longitudinal striation | only at LAP | distalwards projecting far from distal edge of zygocondyles (e.g. Ophiacantha) | with single ridge | nearly parallel | present and fused with pair of zygocondyles | not projecting beyond ventral edge of zygoc. or projecting beyond ventral edge of zygoc. with projecting part shorter than zygoc. | no | arched | yes | no | no | protruding to form knobs larger than stereom pores on small part of outer surface of LAP | formed by regular ridges | no | yes, over most of the proximal edge | 1 or 2 small spurs | absent | not protruding | no | on elevated portion bordered prox. by ridge | separated from distal edge by the usual outer surface stereom | arranged over entire distal LAP edge | dorsalwards increasing | dorsalwards increasing | by small ridge if at all | smaller than muscle opening | present | not applicable | not applicable | not applicable | not applicable | merged at their proximal tips by smooth connection | one lobe clearly larger than the other | no, shifted (e.g. Ophiacantha) | no, at least one lobe bent | with perforations | tilted | fully developed | notch beyond the first segments under the disc | ventro-distalwards | no | no | more or less continuous ridge | with ventral tip of bentro-proximalwards pointing part of ridge separated from remaining ridge | more compact or more densely meshed stereom | with two kinks and dorsal tip with ventro-proximalwards pointing projection | not applicable | not applicable | vertical row with furrow |
| 15. Ophiactis savignyi | many thin scales | uniform | no | same size/indistinguishable/absent | same size/indistinguishable/absent | not applicable | thin skin, not obscuring plates | with spines only | sparse all over with underlying plates/scales visible or restricted to margin | uniform | present | naked | more than half of the disc radius | separated proximally | half-circle | entire/continuous | distal-adradial portion of RS exposed | naked | longer than half interradius or divided into two openings | as long as adradial plate | bar-like without ridge or groove | concave | fully covered | no | not applicable | not applicable | covering less than one third of interradius | as long as wide | larger than remaining oral shields | yes | obtuse angle with straight to convex sides | with narrower distalward projection | entering mouth slit via shallow embayment or opening deep wihtin mouth slit | no | single row along jaw edge | (fragmented into) several papillae (Ophiura) | absent | scale-like, much larger/wider than other papillae | paddle-shaped | single | none/only ASS | with square tip | entire | equal width all over | single column throughout | surrounded by separate, weakly protruding knobs and/or ridges | at least some perforating DP with septum | as high as long or higher | large, well defined flange | normal stereom | with large, dorsal, spoon-shaped depression | naked | simple | potentially in contact | convex to straight | convex to straight | as wide as proximal portion or narrower | yes | no | with tubercles or striation | no | single | separated | oval semi-circular | tuberculous | no | lateral | predominantly erect, standing perpendicular to arm | 1-2 segments | ? | with lateral thorns | round | blunt | only at distal segments | regular spines with bent tip and/or saw-toothed edge | median spine(s) longest | decreasing distalwards | present | operculiform | not accurately closing tentacle pore | with longitudinal striation | only at LAP | distalwards projecting far from distal edge of zygocondyles (e.g. Ophiacantha) | with single ridge | nearly parallel | present and fused with pair of zygocondyles | not projecting beyond ventral edge of zygoc. or projecting beyond ventral edge of zygoc. with projecting part shorter than zygoc. | no | arched | no | yes | no | protruding to form knobs larger than stereom pores on most of outer surface of LAP | absent | no | yes, only in central part | absent | absent | protruding | yes, but restricted to small area (e.g. between spurs) | on same level as remaining outer surface | directly adjacent to the distal edge of the LAP | arranged over entire distal LAP edge | middle spine art. larger | dorsalwards increasing | by small ridge if at all | approx. as large as muscle opening | present | not applicable | not applicable | not applicable | not applicable | simply separated | one lobe clearly larger than the other | yes (e.g. Amphiura) | no, at least one lobe bent | massive | nearly horizontal | absent | notch beyond the first segments under the disc | ventro-distalwards | no | no | two separate (rarely merged) central knobs | not applicable | not applicable | not applicable | with a knob | no | single large and conspicuous |
| 16. Ophiarachna incrassata | many thin scales | uniform | no | same size/indistinguishable/absent | same size/indistinguishable/absent | not applicable | thin skin, not obscuring plates | with granules only | forming dense cover completely hiding underlying plates/scales (possible exception radial shields) | uniform | present | at least partly covered | less than one third of the disc radius | completely separated | isoscele triangular to pear-shaped | incised/irregular | distal portion of RS exposed | with granules | longer than half interradius or divided into two openings | shorter than half the adradial plate length | bar-like with longitudinal groove ad large perforation | straight or convex | fully covered | no | not applicable | not applicable | covering less than one third of interradius | wider than long | similar to other oral shields | yes | evenly convex | with narrower distalward projection | entering mouth slit via shallow embayment or opening deep wihtin mouth slit | no | single row along jaw edge | (fragmented into) several papillae (Ophiura) | infradental | scale-like, much larger/wider than other papillae | rounded | single | also at 1st VAP | with round or slightly pointed tip (but never spine-like) | fragmented | equal width all over | multiple columns or cluster on max half of plate | surrounded by a more or less continuous proturding ring | depression or perforating DP without septum | longer than high | central depression | normal stereom | ventral, lining ventral or ventro-distal edge of articulation area | naked | simple | potentially in contact | convex to straight | convex to straight | wider than proximal portion | yes | no | with tubercles or striation | yes | single | in contact | trapezoid with smooth proximal edge | tuberculous | yes | lateral | predominantly erect, standing perpendicular to arm | longer than 2 segments | massive | with scale-like tubercles | round | pointed | absent | not applicable | ventralmost spine(s) longest | decreasing distalwards | present | leaf-like | accurately closing tentacle pore | ? | only at LAP | no | with single ridge | nearly parallel | present and fused with pair of zygocondyles | not projecting beyond ventral edge of zygoc. or projecting beyond ventral edge of zygoc. with projecting part shorter than zygoc. | no | arched | no | yes | yes | protruding to form knobs larger than stereom pores on most of outer surface of LAP | absent | no | yes, over most of the proximal edge | 1 or 2 large spurs | present | not protruding | no | in notches of distal LAP edge | directly adjacent to the distal edge of the LAP | arranged over entire distal LAP edge | ventralwards increasing | equidistant | by small ridge if at all | smaller than muscle opening | present | not applicable | not applicable | not applicable | not applicable | merged at their proximal tips by smooth connection | one lobe clearly larger than the other | no, shifted (e.g. Ophiacantha) | no, at least one lobe bent | with perforations | tilted | weakly developed | notch beyond the first segments under the disc | ventro-distalwards | no | no | more or less continuous ridge | with separate knob on ventral tip of LAP | more compact or more densely meshed stereom | without major kink and with tongue-shaped dorsal tip | not applicable | not applicable | vertical row without furrow |
| 17. Ophienigma spinilimbatum | many thin scales | variable | no | larger than disc scales | larger than scales | at a distance from CPP | thin skin, not obscuring plates | with spines only | forming dense cover completely hiding underlying plates/scales (possible exception radial shields) | uniform | present | naked | less than one third of the disc radius | completely separated | half-circle | entire/continuous | distal-adradial portion of RS exposed | naked | longer than half interradius or divided into two openings | longer than half the adradial plate length | bar-like without ridge or groove | concave | exposed | no | not applicable | not applicable | covering less than one third of interradius | wider than long | similar to other oral shields | no, separated | obtuse angle with concave sides | evenly convex | entering mouth slit via shallow embayment or opening deep wihtin mouth slit | no | single row along jaw edge | (fragmented into) several papillae (Ophiura) | infradental | scale-like or like other papillae | spiniform | single | none/only ASS | with square tip | entire | dorsal half widest | single column throughout | surrounded by separate, weakly protruding knobs and/or ridges | depression or perforating DP without septum | longer than high | central depression | normal stereom | in middle position, vertical and lining less than two thirds of distal edge of adradial articulation area | naked | simple | potentially in contact | concave or incised | convex to straight | wider than proximal portion | yes | no | with tubercles or striation | no | single | in contact | fan-shaped | tuberculous | no | lateral | predominantly erect, standing perpendicular to arm | 1-2 segments | massive | smooth | round | pointed | absent | not applicable | dorsalmost spine(s) longest | decreasing distalwards | present | leaf-like | accurately closing tentacle pore | with longitudinal striation | only at LAP | distalwards projecting far from distal edge of zygocondyles (e.g. Ophiacantha) | with single ridge | nearly parallel | present and fused with pair of zygocondyles | not projecting beyond ventral edge of zygoc. or projecting beyond ventral edge of zygoc. with projecting part shorter than zygoc. | no | arched | no | yes | no | protruding to form knobs larger than stereom pores on most of outer surface of LAP | absent | no | yes, only in central part | absent | absent | protruding | no | on same level as remaining outer surface | directly adjacent to the distal edge of the LAP | arranged over entire distal LAP edge | middle spine art. larger | dorsalwards increasing | by small ridge if at all | smaller than muscle opening | present | not applicable | not applicable | not applicable | not applicable | simply separated | one lobe clearly larger than the other | yes (e.g. Amphiura) | no, at least one lobe bent | massive | nearly horizontal | absent | notch beyond the first segments under the disc | ventro-distalwards | no | yes | two separate (rarely merged) central knobs | not applicable | not applicable | not applicable | with a knob | no | vertical row without furrow |
| 18. Ophiochiton fastigatus | many thin scales | variable | no | same size/indistinguishable/absent | larger than scales | at a distance from CPP | thin skin, not obscuring plates | without granules/spines | not applicable | not applicable | present | naked | between one third and half of the disc radius | completely separated | scalene triangular | entire/continuous | distal-adradial portion of RS exposed | naked | longer than half interradius or divided into two openings | as long as adradial plate | bar-like without ridge or groove | concave | fully covered | no | not applicable | not applicable | covering less than one third of interradius | wider than long | larger than remaining oral shields | yes | acute to right angle with convex sides | with narrower distalward projection | entering mouth slit via shallow embayment or opening deep wihtin mouth slit | no | single row along jaw edge | (fragmented into) several papillae (Ophiura) | lateral | scale-like, much larger/wider than other papillae | spiniform | single | also at 1st VAP | with square tip | entire | equal width all over | single column throughout | surrounded by separate, weakly protruding knobs and/or ridges | depression or perforating DP without septum | longer than high | central depression | normal stereom | in middle position, vertical and lining less than two thirds of distal edge of adradial articulation area | naked | simple | potentially in contact | convex to straight | convex to straight | wider than proximal portion | yes | no | without conspicuous ornamentation | no | single | in contact | oval semi-circular | without conspicuous ornamentation | no | lateral | predominantly erect, standing perpendicular to arm | 1-2 segments | ? | smooth | round | pointed | absent | not applicable | dorsalmost spine(s) longest | decreasing distalwards | present | operculiform | accurately closing tentacle pore | without longitudinal striation | at both LAP and VAP | distalwards projecting almost beyond zygocondyles (e.g.. Ophiodoris) | with single ridge | dorsalwards converging | present and fused with pair of zygocondyles | projecting beyond ventral edge of zygoc. with projecting part as long as zygoc. | no | arched | no | yes | yes | protruding to form only knobs approx. the same size as stereom pores | absent | no | yes, only in central part | absent | present | not protruding | yes, along most of the edge | on same level as remaining outer surface | directly adjacent to the distal edge of the LAP | arranged over entire distal LAP edge | middle spine art. larger | dorsalwards increasing | by small ridge if at all | smaller than muscle opening | present | not applicable | not applicable | not applicable | not applicable | separated by one or several knobs or by denticulate stereom | equal-sized | yes (e.g. Amphiura) | yes | with perforations | nearly horizontal | absent | notch beyond the first segments under the disc | ventro-distalwards | no | no | more or less continuous ridge | with ventral tip of bentro-proximalwards pointing part of ridge separated from remaining ridge | more compact or more densely meshed stereom | with two kinks and dorsal kink with ventro-proximalwards pointing projection | not applicable | not applicable | single large and conspicuous |
| 19. Ophiochondrus stelliger | many thin scales | uniform | no | same size/indistinguishable/absent | same size/indistinguishable/absent | not applicable | thin skin, not obscuring plates | with granules only | forming dense cover completely hiding underlying plates/scales (possible exception radial shields) | uniform | present | at least partly covered | more than half of the disc radius | completely separated | isoscele triangular to pear-shaped | entire/continuous | distal portion of RS exposed | with granules | longer than half interradius or divided into two openings | as long as adradial plate | bar-like without ridge or groove | straight or convex | fully covered | no | not applicable | not applicable | longer than one third of length of interradius | wider than long | larger than remaining oral shields | yes | obtuse angle with concave sides | evenly convex | entering mouth slit via shallow embayment or opening deep wihtin mouth slit | no | single row along jaw edge | (fragmented into) several papillae (Ophiura) | absent | scale-like or like other papillae | block-shaped | single | none/only ASS | with round or slightly pointed tip (but never spine-like) | entire | equal width all over | single column throughout | surrounded by separate, weakly protruding knobs and/or ridges | depression or perforating DP without septum | longer than high | central depression | normal stereom | ventral, lining ventral or ventro-distal edge of articulation area | naked | simple | separated by lateral arm plates | concave or incised | convex to straight | wider than proximal portion | yes | no | with tubercles or striation | no | single | separated | fan-shaped | without conspicuous ornamentation | no | lateral | predominantly erect, standing perpendicular to arm | between half a segment and one segment | ? | with lateral thorns | round | pointed | only at distal segments | regular spines with bent tip and/or saw-toothed edge | median spine(s) longest | decreasing distalwards | present | spine-like | accurately closing tentacle pore | with longitudinal striation | only at LAP | distalwards projecting far from distal edge of zygocondyles (e.g. Ophiacantha) | with single ridge | nearly parallel | present and fused with pair of zygocondyles | not projecting beyond ventral edge of zygoc. or projecting beyond ventral edge of zygoc. with projecting part shorter than zygoc. | no | arched | yes | no | no | protruding to form knobs larger than stereom pores on small part of outer surface of LAP | formed by merged knobs | no | yes, over most of the proximal edge | 1 or 2 small spurs | absent | not protruding | yes, but restricted to small area (e.g. between spurs) | on elevated portion bordered prox. by ridge | separated from distal edge by the usual outer surface stereom | arranged over entire distal LAP edge | dorsalwards increasing | dorsalwards increasing | by small ridge if at all | smaller than muscle opening | present | not applicable | not applicable | not applicable | not applicable | merged at their proximal tips by smooth connection | one lobe clearly larger than the other | yes (e.g. Amphiura) | no, at least one lobe bent | with perforations | tilted | fully developed | notch beyond the first segments under the disc | ventro-distalwards | no | no | more or less continuous ridge | with ventral tip of bentro-proximalwards pointing part of ridge separated from remaining ridge | same stereom as remaining inner surface of LAP | with two kinks and dorsal tip with ventro-proximalwards pointing projection | not applicable | not applicable | vertical row without furrow |
| 20. Ophiocopa spatula | many thin scales | uniform | no | larger than disc scales | same size/indistinguishable/absent | not applicable | thin skin, not obscuring plates | with granules only | sparse all over with underlying plates/scales visible or restricted to margin | uniform | present | naked | less than one third of the disc radius | in contact over entire length | isoscele triangular to pear-shaped | entire/continuous | distal portion of RS exposed | naked | longer than half interradius or divided into two openings | longer than half the adradial plate length | bar-like without ridge or groove | straight or convex | fully covered | no | not applicable | not applicable | longer than one third of length of interradius | as long as wide | similar to other oral shields | yes | acute angle with straight to convex sides | with narrower distalward projection | entering mouth slit via shallow embayment or opening deep wihtin mouth slit | no | single row along jaw edge | (fragmented into) several papillae (Ophiura) | absent | scale-like, much larger/wider than other papillae | rounded | single | none/only ASS | with round or slightly pointed tip (but never spine-like) | entire | equal width all over | single column throughout | surrounded by separate, weakly protruding knobs and/or ridges | depression or perforating DP without septum | longer than high | central depression | normal stereom | ventral, lining ventral or ventro-distal edge of articulation area | naked | simple | separated by lateral arm plates | convex to straight | convex to straight | wider than proximal portion | yes | no | with tubercles or striation | no | single | separated | fan-shaped | tuberculous | no | lateral | predominantly erect, standing perpendicular to arm | longer than 2 segments | with lumen | with lateral thorns | laterally flattened | blunt | absent | not applicable | dorsalmost spine(s) longest | decreasing distalwards | present | operculiform | accurately closing tentacle pore | with longitudinal striation | only at LAP | distalwards projecting far from distal edge of zygocondyles (e.g. Ophiacantha) | with single ridge | nearly parallel | present and fused with pair of zygocondyles | not projecting beyond ventral edge of zygoc. or projecting beyond ventral edge of zygoc. with projecting part shorter than zygoc. | no | arched | yes | yes | no | protruding to form knobs larger than stereom pores on small part of outer surface of LAP | formed by regular ridges | no | yes, over most of the proximal edge | 1 or 2 small spurs | absent | not protruding | no | on elevated portion bordered prox. by ridge | separated from distal edge by the usual outer surface stereom | arranged over entire distal LAP edge | dorsalwards increasing | dorsalwards increasing | by small ridge if at all | smaller than muscle opening | present | not applicable | not applicable | not applicable | not applicable | merged at their proximal tips by smooth connection | one lobe clearly larger than the other | no, shifted (e.g. Ophiacantha) | no, at least one lobe bent | with perforations | tilted | fully developed | notch beyond the first segments under the disc | ventro-distalwards | no | yes | more or less continuous ridge | with ventral tip of bentro-proximalwards pointing part of ridge separated from remaining ridge | more compact or more densely meshed stereom | with two kinks and dorsal tip with ventro-proximalwards pointing projection | not applicable | not applicable | vertical row with furrow |
| 21. Ophiocoma echinata | many thin scales | uniform | no | same size/indistinguishable/absent | same size/indistinguishable/absent | not applicable | thin skin, not obscuring plates | with granules only | forming dense cover completely hiding underlying plates/scales (possible exception radial shields) | uniform | present | at least partly covered | less than one third of the disc radius | completely separated | isoscele triangular to pear-shaped | incised/irregular | distal portion of RS exposed | with granules | longer than half interradius or divided into two openings | longer than half the adradial plate length | bar-like with longitudinal groove ad large perforation | straight or convex | fully covered | no | not applicable | not applicable | covering less than one third of interradius | as long as wide | similar to other oral shields | yes | evenly convex | evenly convex | entering mouth slit via shallow embayment or opening deep wihtin mouth slit | no | single row along jaw edge | (fragmented into) several papillae (Ophiura) | absent | scale-like, much larger/wider than other papillae | rounded | tooth papillae | none/only ASS | with square tip | entire | equal width all over | multiple columns or cluster on max half of plate | surrounded by strongly protruding knobs and/or ridges | at least some perforating DP with septum | as high as long or higher | large, well defined flange | normal stereom | with large, dorsal, spoon-shaped depression | naked | simple | potentially in contact | convex to straight | convex to straight | wider than proximal portion | yes | no | with tubercles or striation | no | single | in contact | trapezoid with smooth proximal edge | tuberculous | yes | lateral | predominantly erect, standing perpendicular to arm | longer than 2 segments | massive | with scale-like tubercles | round | pointed | absent | not applicable | dorsalmost spine(s) longest | decreasing distalwards | present | operculiform | accurately closing tentacle pore | without longitudinal striation | only at LAP | no | with single ridge | nearly parallel | present and fused with pair of zygocondyles | not projecting beyond ventral edge of zygoc. or projecting beyond ventral edge of zygoc. with projecting part shorter than zygoc. | no | arched | no | yes | no | protruding to form knobs larger than stereom pores on most of outer surface of LAP | absent | no | yes, over most of the proximal edge | absent | present | not protruding | no | on elevation not bordered by ridge | directly adjacent to the distal edge of the LAP | arranged over entire distal LAP edge | dorsalwards increasing | dorsalwards increasing | by small ridge if at all | smaller than muscle opening | present | not applicable | not applicable | not applicable | not applicable | merged at their proximal tips by smooth connection | one lobe clearly larger than the other | no, shifted (e.g. Ophiacantha) | no, at least one lobe bent | with perforations | tilted | fully developed | notch beyond the first segments under the disc | ventro-distalwards | no | yes | more or less continuous ridge | with separate knob on ventral tip of LAP | more compact or more densely meshed stereom | with two kinks and dorsal kink with ventro-proximalwards pointing projection | not applicable | not applicable | single large and conspicuous |
| 22. Ophiocomina nigra | many thin scales | uniform | no | same size/indistinguishable/absent | same size/indistinguishable/absent | not applicable | thin skin, not obscuring plates | with granules only | forming dense cover completely hiding underlying plates/scales (possible exception radial shields) | uniform | present | at least partly covered | less than one third of the disc radius | completely separated | isoscele triangular to pear-shaped | entire/continuous | distal portion of RS exposed | naked | longer than half interradius or divided into two openings | longer than half the adradial plate length | bar-like without ridge or groove | concave | fully covered | no | not applicable | not applicable | covering less than one third of interradius | wider than long | larger than remaining oral shields | yes | obtuse angle with concave sides | evenly convex | entering mouth slit via shallow embayment or opening deep wihtin mouth slit | no | single row along jaw edge | (fragmented into) several papillae (Ophiura) | absent | scale-like, much larger/wider than other papillae | spiniform | tooth papillae | only at AS | with square tip | entire | ventral half widest | multiple columns or cluster on max half of plate | surrounded by a more or less continuous proturding ring | depression or perforating DP without septum | longer than high | central depression | normal stereom | ventral, lining ventral or ventro-distal edge of articulation area | naked | simple | potentially in contact | concave or incised | convex to straight | wider than proximal portion | yes | no | without conspicuous ornamentation | no | single | in contact | fan-shaped | without conspicuous ornamentation | no | lateral | predominantly erect, standing perpendicular to arm | longer than 2 segments | massive | with lateral thorns | round | pointed | absent | not applicable | dorsalmost spine(s) longest | decreasing distalwards | present | leaf-like | accurately closing tentacle pore | with longitudinal striation | only at LAP | distalwards projecting far from distal edge of zygocondyles (e.g. Ophiacantha) | with single ridge | nearly parallel | present and fused with pair of zygocondyles | not projecting beyond ventral edge of zygoc. or projecting beyond ventral edge of zygoc. with projecting part shorter than zygoc. | no | arched | yes | yes | no | protruding to form only knobs approx. the same size as stereom pores | formed by regular ridges | no | yes, over most of the proximal edge | absent | absent | not protruding | no | on elevated portion bordered prox. by ridge | separated from distal edge by the usual outer surface stereom | arranged over entire distal LAP edge | dorsalwards increasing | dorsalwards increasing | by small ridge if at all | smaller than muscle opening | present | not applicable | not applicable | not applicable | not applicable | merged at their proximal tips by smooth connection | one lobe clearly larger than the other | no, shifted (e.g. Ophiacantha) | no, at least one lobe bent | with perforations | tilted | fully developed | notch beyond the first segments under the disc | ventro-distalwards | no | yes | more or less continuous ridge | with ventral tip of bentro-proximalwards pointing part of ridge separated from remaining ridge | more compact or more densely meshed stereom | with two kinks and dorsal kink with ventro-proximalwards pointing projection | not applicable | not applicable | vertical row with furrow |
| 23. Ophiocten sericeum | many thin scales | variable | no | larger than disc scales | larger than scales | at a distance from CPP | thin skin, not obscuring plates | without granules/spines | not applicable | not applicable | present | naked | less than one third of the disc radius | completely separated | isoscele triangular to pear-shaped | entire/continuous | central part of RS to almost entire RS exposed | naked | longer than half interradius or divided into two openings | longer than half the adradial plate length | bar-like with longitudinal ridge | straight or convex | exposed | papillae | yes | spine-like | longer than one third of length of interradius | longer than wide | similar to other oral shields | yes | acute to right angle with convex sides | evenly convex | opening completely outside mouth slit | yes | single row along jaw edge | (fragmented into) several papillae (Ophiura) | lateral | scale-like or like other papillae | block-shaped | single | none/only ASS | with round or slightly pointed tip (but never spine-like) | entire | equal width all over | multiple columns throughout | surrounded by a more or less continuous proturding ring | at least some perforating DP with septum | longer than high | central depression | normal stereom | ventral, lining ventral or ventro-distal edge of articulation area | naked | simple | separated by lateral arm plates | convex to straight | convex to straight | wider than proximal portion | no | not applicable | without conspicuous ornamentation | no | single | in contact | trapezoid with smooth proximal edge | without conspicuous ornamentation | no | lateral | predominantly erect, standing perpendicular to arm | 1-2 segments | massive | smooth | round | pointed | absent | not applicable | dorsalmost spine(s) longest | constant | present | leaf-like | not accurately closing tentacle pore | without longitudinal striation | only at LAP | distalwards projecting far from distal edge of zygocondyles (e.g. Ophiacantha) | with single ridge | nearly parallel | present and fused with pair of zygocondyles | projecting beyond ventral edge of zygoc. with projecting part longer than zygoc. | no | arched | no | no | no | protruding to form only knobs approx. the same size as stereom pores | formed by regular ridges | no | yes, over most of the proximal edge | absent | present | not protruding | no | in notches of distal LAP edge | separated from distal edge by a thin projection of the distal LAP portion (e.g. Ophiomyces) | restricted to ventral or central portion of distal LAP edge | all similar | ventralwards increasing | by large, prominent ridge or regular stereom | smaller than muscle opening | absent (e.g. Ophiura) | no | vertical mouth-shaped, sharply defined elevation | vertical | slender | not applicable | not applicable | not applicable | not applicable | not applicable | not applicable | not applicable | notch beyond the first segments under the disc | distalwards, positioned close to the horizontal midline of the LAP | no | no | more or less continuous ridge | ridge separated into two halves | more compact or more densely meshed stereom | without major kink and with tongue-shaped dorsal tip | not applicable | not applicable | vertical row without furrow |
| 24. Ophioderma longicauda | many thin scales | uniform | no | same size/indistinguishable/absent | same size/indistinguishable/absent | not applicable | thin skin, not obscuring plates | with granules only | forming dense cover completely hiding underlying plates/scales (possible exception radial shields) | uniform | present | at least partly covered | less than one third of the disc radius | completely separated | isoscele triangular to pear-shaped | incised/irregular | distal portion of RS exposed | with granules | longer than half interradius or divided into two openings | as long as adradial plate | bar-like with longitudinal groove ad large perforation | straight or convex | fully covered | no | not applicable | not applicable | covering less than one third of interradius | wider than long | similar to other oral shields | yes | evenly convex | with narrower distalward projection | entering mouth slit via shallow embayment or opening deep wihtin mouth slit | no | single row along jaw edge | (fragmented into) several papillae (Ophiura) | infradental | scale-like or like other papillae | spiniform | single | none/only ASS | with square tip | fragmented | equal width all over | multiple columns or cluster on max half of plate | surrounded by a more or less continuous proturding ring | depression or perforating DP without septum | longer than high | central depression | normal stereom | ventral, lining ventral or ventro-distal edge of articulation area | naked | simple | potentially in contact | convex to straight | convex to straight | wider than proximal portion | yes | no | with tubercles or striation | yes | single | in contact | trapezoid with smooth proximal edge | tuberculous | yes | lateral | predominantly parallel to arm axis (adpressed) | shorter than half a segment | massive | with scale-like tubercles | laterally flattened | blunt | absent | not applicable | ventralmost spine(s) longest | decreasing distalwards | present | operculiform | accurately closing tentacle pore | without longitudinal striation | only at LAP | no | with single ridge | dorsalwards converging | present and fused with pair of zygocondyles | not projecting beyond ventral edge of zygoc. or projecting beyond ventral edge of zygoc. with projecting part shorter than zygoc. | no | arched | no | yes | yes | protruding to form knobs larger than stereom pores on most of outer surface of LAP | absent | no | yes, over most of the proximal edge | 1 or 2 large spurs | present | not protruding | yes, but restricted to small area (e.g. between spurs) | in notches of distal LAP edge | directly adjacent to the distal edge of the LAP | arranged over entire distal LAP edge | ventralwards increasing | equidistant | by small ridge if at all | smaller than muscle opening | present | not applicable | not applicable | not applicable | not applicable | merged at their proximal tips by smooth connection | one lobe clearly larger than the other | no, shifted (e.g. Ophiacantha) | no, at least one lobe bent | with perforations | tilted | weakly developed | notch beyond the first segments under the disc | ventro-distalwards | no | no | more or less continuous ridge | with separate knob on ventral tip of LAP | more compact or more densely meshed stereom | without major kink and with tongue-shaped dorsal tip | not applicable | not applicable | vertical row without furrow |
| 25. Ophiodoris malignus | many thin scales | uniform | no | larger than disc scales | same size/indistinguishable/absent | not applicable | thin skin, not obscuring plates | without granules/spines | not applicable | not applicable | present | naked | less than one third of the disc radius | completely separated | scalene triangular | entire/continuous | distal-adradial portion of RS exposed | naked | longer than half interradius or divided into two openings | longer than half the adradial plate length | bar-like without ridge or groove | concave | fully covered | disc granules | no | granule-like | covering less than one third of interradius | as long as wide | similar to other oral shields | no, separated | evenly convex | with narrower distalward projection | entering mouth slit via shallow embayment or opening deep wihtin mouth slit | no | single row along jaw edge | (fragmented into) several papillae (Ophiura) | infradental | scale-like, much larger/wider than other papillae | rounded | single | only at AS | with square tip | entire | equal width all over | single column throughout | surrounded by separate, weakly protruding knobs and/or ridges | depression or perforating DP without septum | longer than high | central depression | normal stereom | in middle position, vertical and lining less than two thirds of distal edge of adradial articulation area | naked | simple | potentially in contact | convex to straight | convex to straight | wider than proximal portion | yes | no | without conspicuous ornamentation | no | single | in contact | oval semi-circular | without conspicuous ornamentation | no | lateral | predominantly erect, standing perpendicular to arm | 1-2 segments | massive | smooth | round | pointed | absent | not applicable | median spine(s) longest | decreasing distalwards | present | operculiform | accurately closing tentacle pore | with longitudinal striation | at both LAP and VAP | distalwards projecting almost beyond zygocondyles (e.g.. Ophiodoris) | with single ridge | dorsalwards converging | present and fused with pair of zygocondyles | projecting beyond ventral edge of zygoc. with projecting part as long as zygoc. | no | arched | no | yes | no | protruding to form knobs larger than stereom pores on most of outer surface of LAP | absent | no | yes, only in central part | absent | present | not protruding | yes, but restricted to small area (e.g. between spurs) | on same level as remaining outer surface | directly adjacent to the distal edge of the LAP | arranged over entire distal LAP edge | middle spine art. larger | dorsalwards increasing | by small ridge if at all | smaller than muscle opening | present | not applicable | not applicable | not applicable | not applicable | separated by one or several knobs or by denticulate stereom | one lobe clearly larger than the other | yes (e.g. Amphiura) | yes | with perforations | nearly horizontal | absent | notch beyond the first segments under the disc | ventro-distalwards | no | yes | more or less continuous ridge | with ventral tip of bentro-proximalwards pointing part of ridge separated from remaining ridge | more compact or more densely meshed stereom | with two kinks and dorsal kink with ventro-proximalwards pointing projection | not applicable | not applicable | single large and conspicuous |
| 26. Ophiolepis superba | thick scales | variable | no | larger than disc scales | same size/indistinguishable/absent | not applicable | thin skin, not obscuring plates | without granules/spines | not applicable | not applicable | present | naked | between one third and half of the disc radius | completely separated | isoscele triangular to pear-shaped | entire/continuous | central part of RS to almost entire RS exposed | naked | longer than half interradius or divided into two openings | as long as adradial plate | bar-like without ridge or groove | concave | exposed | no | not applicable | not applicable | covering less than one third of interradius | longer than wide | similar to other oral shields | yes | acute angle with straight to convex sides | evenly convex | entering mouth slit via shallow embayment or opening deep wihtin mouth slit | no | single row along jaw edge | (fragmented into) several papillae (Ophiura) | infradental | scale-like, much larger/wider than other papillae | rounded | single | only at AS | with square tip | entire | dorsal half widest | single column throughout | surrounded by separate, weakly protruding knobs and/or ridges | depression or perforating DP without septum | longer than high | central depression | normal stereom | in middle position, vertical and lining less than two thirds of distal edge of adradial articulation area | naked | simple | potentially in contact | convex to straight | convex to straight | wider than proximal portion | yes | no | without conspicuous ornamentation | no | multiple | in contact | trapezoid with smooth proximal edge | without conspicuous ornamentation | no | lateral | predominantly parallel to arm axis (adpressed) | shorter than half a segment | massive | ? | round | pointed | absent | not applicable | ventralmost spine(s) longest | decreasing distalwards | present | operculiform | accurately closing tentacle pore | without longitudinal striation | only at LAP | no | with single ridge | dorsalwards converging | present and fused with pair of zygocondyles | not projecting beyond ventral edge of zygoc. or projecting beyond ventral edge of zygoc. with projecting part shorter than zygoc. | no | arched | no | yes | no | protruding to form only knobs approx. the same size as stereom pores | absent | no | yes, over most of the proximal edge | 1 or 2 large spurs | present | not protruding | yes, but restricted to small area (e.g. between spurs) | in notches of distal LAP edge | directly adjacent to the distal edge of the LAP | arranged over entire distal LAP edge | middle spine art. larger | dorsalwards increasing | by small ridge if at all | approx. as large as muscle opening | present | not applicable | not applicable | not applicable | not applicable | separated by one or several knobs or by denticulate stereom | equal-sized | yes (e.g. Amphiura) | yes | with perforations | nearly horizontal | absent | notch beyond the first segments under the disc | ventro-distalwards | no | no | more or less continuous ridge | with separate knob on ventral tip of LAP | same stereom as remaining inner surface of LAP | without major kink and with tongue-shaped dorsal tip | not applicable | not applicable | small or inconspicuous |
| 27. Ophioleuce seminudum | many thin scales | variable | no | same size/indistinguishable/absent | larger than scales | at a distance from CPP | thin skin, not obscuring plates | with granules only | forming dense cover completely hiding underlying plates/scales (possible exception radial shields) | uniform | present | at least partly covered | less than one third of the disc radius | completely separated | scalene triangular | entire/continuous | distal-adradial portion of RS exposed | with granules | longer than half interradius or divided into two openings | longer than half the adradial plate length | bar-like without ridge or groove | concave | fully covered | disc granules | no | granule-like | covering less than one third of interradius | longer than wide | similar to other oral shields | yes | acute angle with straight to convex sides | with narrower distalward projection | entering mouth slit via shallow embayment or opening deep wihtin mouth slit | no | single row along jaw edge | (fragmented into) several papillae (Ophiura) | infradental | scale-like or like other papillae | block-shaped | single | none/only ASS | spine-shaped | entire | ventral half widest | single column throughout | simple opening | depression or perforating DP without septum | longer than high | central depression | normal stereom | ventral, lining ventral or ventro-distal edge of articulation area | naked | simple | separated by lateral arm plates | convex to straight | convex to straight | wider than proximal portion | yes | no | with tubercles or striation | no | single | in contact | trapezoid with smooth proximal edge | with striation | ? | lateral | predominantly parallel to arm axis (adpressed) | between half a segment and one segment | massive | with lateral thorns | round | pointed | absent | not applicable | all equal | decreasing distalwards | present | operculiform | accurately closing tentacle pore | with longitudinal striation | only at LAP | distalwards projecting far from distal edge of zygocondyles (e.g. Ophiacantha) | with single ridge | dorsalwards converging | present and fused with pair of zygocondyles | not projecting beyond ventral edge of zygoc. or projecting beyond ventral edge of zygoc. with projecting part shorter than zygoc. | no | arched | no | yes | yes | protruding to form knobs larger than stereom pores on most of outer surface of LAP | formed by merged knobs | yes | yes, over most of the proximal edge | 1 or 2 small spurs | absent | not protruding | yes, along most of the edge | on same level as remaining outer surface | separated from distal edge by a thin projection of the distal LAP portion (e.g. Ophiomyces) | arranged over entire distal LAP edge | ventralwards increasing | only 2 | by small ridge if at all | smaller than muscle opening | present | not applicable | not applicable | not applicable | not applicable | merged at their proximal tips by smooth connection | one lobe clearly larger than the other | no, shifted (e.g. Ophiacantha) | no, at least one lobe bent | massive | nearly vertical | absent | notch beyond the first segments under the disc | ventro-distalwards | no | yes | more or less continuous ridge | with ventral tip of bentro-proximalwards pointing part of ridge separated from remaining ridge | more compact or more densely meshed stereom | with kink between dorso-proximalwards pointing dorsal portion and ventro-proximalwards pointing ventral portion | not applicable | not applicable | small or inconspicuous |
| 28. Ophiolimna bairdi | many thin scales | uniform | no | same size/indistinguishable/absent | same size/indistinguishable/absent | not applicable | thin skin, not obscuring plates | with both granules and spines | forming dense cover completely hiding underlying plates/scales (possible exception radial shields) | uniform | present | at least partly covered | less than one third of the disc radius | completely separated | isoscele triangular to pear-shaped | entire/continuous | distal portion of RS exposed | with granules | longer than half interradius or divided into two openings | longer than half the adradial plate length | bar-like without ridge or groove | straight or convex | fully covered | no | not applicable | not applicable | longer than one third of length of interradius | as long as wide | similar to other oral shields | yes | obtuse angle with concave sides | evenly convex | entering mouth slit via shallow embayment or opening deep wihtin mouth slit | no | single row along jaw edge | (fragmented into) several papillae (Ophiura) | lateral | scale-like, much larger/wider than other papillae | spiniform | single | none/only ASS | with round or slightly pointed tip (but never spine-like) | entire | ventral half widest | single column throughout | surrounded by separate, weakly protruding knobs and/or ridges | depression or perforating DP without septum | longer than high | central depression | normal stereom | ventral, lining ventral or ventro-distal edge of articulation area | naked | simple | separated by lateral arm plates | convex to straight | convex to straight | wider than proximal portion | yes | no | with tubercles or striation | no | single | separated | fan-shaped | with striation | no | lateral | predominantly erect, standing perpendicular to arm | 1-2 segments | with lumen | with lateral thorns | round | pointed | absent | not applicable | dorsalmost spine(s) longest | decreasing distalwards | present | leaf-like | accurately closing tentacle pore | with longitudinal striation | only at LAP | distalwards projecting far from distal edge of zygocondyles (e.g. Ophiacantha) | with single ridge | nearly parallel | present and fused with pair of zygocondyles | not projecting beyond ventral edge of zygoc. or projecting beyond ventral edge of zygoc. with projecting part shorter than zygoc. | no | arched | yes | yes | no | protruding to form only knobs approx. the same size as stereom pores | formed by regular ridges | no | yes, over most of the proximal edge | absent | absent | not protruding | yes, but restricted to small area (e.g. between spurs) | on elevated portion bordered prox. by ridge | separated from distal edge by the usual outer surface stereom | arranged over entire distal LAP edge | dorsalwards increasing | dorsalwards increasing | by small ridge if at all | smaller than muscle opening | present | not applicable | not applicable | not applicable | not applicable | merged at their proximal tips by smooth connection | one lobe clearly larger than the other | no, shifted (e.g. Ophiacantha) | no, at least one lobe bent | massive | tilted | fully developed | notch beyond the first segments under the disc | ventro-distalwards | no | no | more or less continuous ridge | with ventral tip of bentro-proximalwards pointing part of ridge separated from remaining ridge | more compact or more densely meshed stereom | with two kinks and dorsal tip with ventro-proximalwards pointing projection | not applicable | not applicable | vertical row with furrow |
| 29. Ophiolycus purpureus | many thin scales | uniform | no | same size/indistinguishable/absent | same size/indistinguishable/absent | not applicable | thick skin, obscuring scales/plates | with granules only | sparse all over with underlying plates/scales visible or restricted to margin | uniform | present | at least partly covered | less than one third of the disc radius | completely separated | isoscele triangular to pear-shaped | entire/continuous | distal portion of RS exposed | with granules | longer than half interradius or divided into two openings | as long as adradial plate | bar-like without ridge or groove | straight or convex | fully covered | no | not applicable | not applicable | covering less than one third of interradius | wider than long | larger than remaining oral shields | yes | obtuse angle with concave sides | with narrower distalward projection | entering mouth slit via shallow embayment or opening deep wihtin mouth slit | no | single row along jaw edge | (fragmented into) several papillae (Ophiura) | lateral | spiniform | spiniform | several | none/only ASS | with round or slightly pointed tip (but never spine-like) | entire | ventral half widest | multiple columns or cluster on max half of plate | surrounded by a more or less continuous proturding ring | depression or perforating DP without septum | longer than high | central depression | normal stereom | ventral, lining ventral or ventro-distal edge of articulation area | naked | simple | potentially in contact | concave or incised | convex to straight | wider than proximal portion | yes | no | without conspicuous ornamentation | ? | multiple | in contact | trapezoid with smooth proximal edge | without conspicuous ornamentation | ? | lateral | predominantly erect, standing perpendicular to arm | 1-2 segments | massive | with lateral thorns | round | blunt | only at distal segments | true, hyaline hook | median spine(s) longest | decreasing distalwards | present | spine-like | not accurately closing tentacle pore | with longitudinal striation | only at LAP | distalwards projecting far from distal edge of zygocondyles (e.g. Ophiacantha) | with single ridge | dorsalwards converging | present and fused with pair of zygocondyles | not projecting beyond ventral edge of zygoc. or projecting beyond ventral edge of zygoc. with projecting part shorter than zygoc. | no | arched | yes | yes | yes | not protruding | absent | no | no | absent | present | not protruding | yes, but restricted to small area (e.g. between spurs) | on same level as remaining outer surface | separated from distal edge by the usual outer surface stereom | arranged over entire distal LAP edge | dorsalwards increasing | dorsalwards increasing | by small ridge if at all | smaller than muscle opening | present | not applicable | not applicable | not applicable | not applicable | merged at their proximal tips by smooth connection | one lobe clearly larger than the other | no, shifted (e.g. Ophiacantha) | no, at least one lobe bent | with perforations | tilted | weakly developed | notch beyond the first segments under the disc | ventro-distalwards | no | yes | more or less continuous ridge | with ventral tip of bentro-proximalwards pointing part of ridge separated from remaining ridge | more compact or more densely meshed stereom | with kink between dorso-proximalwards pointing dorsal portion and ventro-proximalwards pointing ventral portion | not applicable | not applicable | vertical row without furrow |
| 30. Ophiomusium Fossil | few thin scales | variable | no | larger than disc scales | same size/indistinguishable/absent | not applicable | ? | without granules/spines | not applicable | not applicable | present | naked | between one third and half of the disc radius | completely separated | isoscele triangular to pear-shaped | entire/continuous | central part of RS to almost entire RS exposed | naked | longer than half interradius or divided into two openings | longer than half the adradial plate length | bar-like with longitudinal ridge | straight or convex | exposed | papillae | yes | block-like | longer than one third of length of interradius | longer than wide | similar to other oral shields | yes | acute angle with straight to convex sides | with narrower distalward projection | entering mouth slit via shallow embayment or opening deep wihtin mouth slit | no | single row along jaw edge | (fragmented into) several papillae (Ophiura) | lateral | scale-like, much larger/wider than other papillae | block-shaped | single | none/only ASS | ? | entire | ? | single column throughout | surrounded by a more or less continuous proturding ring | depression or perforating DP without septum | longer than high | central depression | normal stereom | ventral, lining ventral or ventro-distal edge of articulation area | naked | simple | separated by lateral arm plates | convex to straight | convex to straight | wider than proximal portion | yes | no | without conspicuous ornamentation | no | single | in contact | fan-shaped | without conspicuous ornamentation | no | lateral | predominantly parallel to arm axis (adpressed) | shorter than half a segment | massive | smooth | round | blunt | absent | not applicable | ventralmost spine(s) longest | decreasing distalwards | present | operculiform | accurately closing tentacle pore | without longitudinal striation | only at LAP | distalwards projecting far from distal edge of zygocondyles (e.g. Ophiacantha) | with single ridge | dorsalwards converging | present and fused with pair of zygocondyles | projecting beyond ventral edge of zygoc. with projecting part as long as zygoc. | no | arched | no | no | no | protruding to form only knobs approx. the same size as stereom pores | absent | no | yes, over most of the proximal edge | more than two small spurs | present | not protruding | yes, but restricted to small area (e.g. between spurs) | on same level as remaining outer surface | separated from distal edge by the usual outer surface stereom | restricted to ventral or central portion of distal LAP edge | all similar | equidistant | by large, prominent ridge or regular stereom | approx. as large as muscle opening | absent (e.g. Ophiura) | yes | vertical mouth-shaped, sharply defined elevation | vertical | thick, lip-shaped and strongly protruding | not applicable | not applicable | not applicable | not applicable | not applicable | not applicable | not applicable | within-pore perforation beyond first segments under the disc | ventralwards | yes | no | more or less continuous ridge | entire | more compact or more densely meshed stereom | without major kink and with tongue-shaped dorsal tip | not applicable | not applicable | small or inconspicuous |
| 31. Ophiomusium lymani | thick scales | variable | no | larger than disc scales | larger than scales | at a distance from CPP | thin skin, not obscuring plates | without granules/spines | not applicable | not applicable | present | naked | more than half of the disc radius | completely separated | isoscele triangular to pear-shaped | entire/continuous | central part of RS to almost entire RS exposed | naked | shorter than half interradius | as long as adradial plate | bar-like with longitudinal ridge | straight or convex | exposed | papillae | no | block-like | longer than one third of length of interradius | longer than wide | similar to other oral shields | yes | acute angle with straight to convex sides | square | entering mouth slit via shallow embayment or opening deep wihtin mouth slit | no | single row along jaw edge | (fragmented into) several papillae (Ophiura) | lateral | scale-like, much larger/wider than other papillae | block-shaped | single | none/only ASS | with round or slightly pointed tip (but never spine-like) | entire | dorsal half widest | single column throughout | surrounded by a more or less continuous proturding ring | depression or perforating DP without septum | longer than high | central depression | normal stereom | ventral, lining ventral or ventro-distal edge of articulation area | naked | simple | separated by lateral arm plates | convex to straight | convex to straight | wider than proximal portion | yes | no | without conspicuous ornamentation | no | single | separated | fan-shaped | without conspicuous ornamentation | no | lateral | predominantly parallel to arm axis (adpressed) | shorter than half a segment | massive | smooth | round | blunt | absent | not applicable | ventralmost spine(s) longest | decreasing distalwards | present | operculiform | accurately closing tentacle pore | without longitudinal striation | only at LAP | no | with single ridge | dorsalwards converging | present and fused with pair of zygocondyles | projecting beyond ventral edge of zygoc. with projecting part as long as zygoc. | no | arched | no | no | no | protruding to form only knobs approx. the same size as stereom pores | absent | no | yes, over most of the proximal edge | more than two small spurs | present | not protruding | no | on same level as remaining outer surface | separated from distal edge by the usual outer surface stereom | arranged over entire distal LAP edge | all similar | equidistant | by large, prominent ridge or regular stereom | approx. as large as muscle opening | absent (e.g. Ophiura) | yes | vertical mouth-shaped, sharply defined elevation | oblique | thick, lip-shaped and strongly protruding | not applicable | not applicable | not applicable | not applicable | not applicable | not applicable | not applicable | within-pore perforation beyond first segments under the disc | ventralwards | yes | no | more or less continuous ridge | entire | more compact or more densely meshed stereom | without major kink and with tongue-shaped dorsal tip | not applicable | not applicable | small or inconspicuous |
| 32. Ophiomyces delata | few thin scales | uniform | no | same size/indistinguishable/absent | same size/indistinguishable/absent | not applicable | thin skin, not obscuring plates | with spines only | sparse all over with underlying plates/scales visible or restricted to margin | uniform | absent | not applicable | not applicable | not applicable | not applicable | not applicable | not applicable | with spines | longer than half interradius or divided into two openings | as long as adradial plate | paddle-shaped | straight or convex | fully covered | no | not applicable | not applicable | covering less than one third of interradius | longer than wide | similar to other oral shields | no, separated | acute angle with straight to convex sides | with narrower distalward projection | entering mouth slit via shallow embayment or opening deep wihtin mouth slit | no | multiple rows covering jaws | (fragmented into) several papillae (Ophiura) | infradental | scale-like or like other papillae | paddle-shaped | single | also at 1st VAP | with round or slightly pointed tip (but never spine-like) | entire | ventral half widest | single column throughout | simple opening | depression or perforating DP without septum | longer than high | central depression | normal stereom | in middle position, vertical and lining less than two thirds of distal edge of adradial articulation area | naked | simple | separated by lateral arm plates | concave or incised | convex to straight | wider than proximal portion | yes | yes | with tubercles or striation | no | single | separated | fan-shaped | without conspicuous ornamentation | no | lateral | predominantly erect, standing perpendicular to arm | 1-2 segments | massive | with lateral thorns | laterally flattened | pointed | absent | not applicable | median spine(s) longest | decreasing distalwards | present | operculiform | not accurately closing tentacle pore | with longitudinal striation | at both LAP and VAP | distalwards projecting far from distal edge of zygocondyles (e.g. Ophiacantha) | with single ridge | nearly parallel | present and fused with pair of zygocondyles | not projecting beyond ventral edge of zygoc. or projecting beyond ventral edge of zygoc. with projecting part shorter than zygoc. | no | arched | no | yes | yes | protruding to form only knobs approx. the same size as stereom pores | formed by regular ridges | no | yes, over most of the proximal edge | absent | present | not protruding | no | on same level as remaining outer surface | separated from distal edge by a thin projection of the distal LAP portion (e.g. Ophiomyces) | arranged over entire distal LAP edge | ventralwards increasing | equidistant | by small ridge if at all | smaller than muscle opening | present | not applicable | not applicable | not applicable | not applicable | merged at their proximal tips by smooth connection | one lobe clearly larger than the other | no, shifted (e.g. Ophiacantha) | no, at least one lobe bent | massive | nearly vertical | absent | notch beyond the first segments under the disc | ventro-distalwards | no | no | more or less continuous ridge | with ventral tip of bentro-proximalwards pointing part of ridge separated from remaining ridge | more compact or more densely meshed stereom | with kink between dorso-proximalwards pointing dorsal portion and ventro-proximalwards pointing ventral portion | not applicable | not applicable | vertical row with furrow |
| 33. Ophiomyxa pentagona | very few thin small scales or none | not applicable | not applicable | same size/indistinguishable/absent | same size/indistinguishable/absent | not applicable | thick skin with few or no scales | without granules/spines | not applicable | not applicable | present | naked | less than one third of the disc radius | completely separated | isoscele triangular to pear-shaped | incised/irregular | distal portion of RS exposed | naked | longer than half interradius or divided into two openings | longer than half the adradial plate length | bar-like with longitudinal groove ad large perforation | straight or convex | fully covered | no | not applicable | not applicable | covering less than one third of interradius | as long as wide | similar to other oral shields | yes | evenly convex | evenly convex | entering mouth slit via shallow embayment or opening deep wihtin mouth slit | no | single row along jaw edge | (fragmented into) several papillae (Ophiura) | lateral | scale-like or like other papillae | block-shaped | single | none/only ASS | with square tip | fragmented | equal width all over | single column throughout | surrounded by a more or less continuous proturding ring | depression or perforating DP without septum | longer than high | central depression | normal stereom | ventral, lining ventral or ventro-distal edge of articulation area | naked | simple | potentially in contact | concave or incised | convex to straight | wider than proximal portion | yes | no | without conspicuous ornamentation | no | single | in contact | trapezoid with smooth proximal edge | without conspicuous ornamentation | no | lateral | predominantly parallel to arm axis (adpressed) | shorter than half a segment | massive | with lateral thorns | round | blunt | only at distal segments | regular spines with bent tip and/or saw-toothed edge | median spine(s) longest | decreasing distalwards | absent | not applicable | not applicable | not applicable | not applicable | no | with single ridge | nearly parallel | present and fused with pair of zygocondyles | not projecting beyond ventral edge of zygoc. or projecting beyond ventral edge of zygoc. with projecting part shorter than zygoc. | no | arched | no | yes | yes | not protruding | absent | no | no | 1 or 2 small spurs | present | not protruding | no | on same level as remaining outer surface | separated from distal edge by the usual outer surface stereom | arranged over entire distal LAP edge | ventralwards increasing | equidistant | by small ridge if at all | approx. as large as muscle opening | present | not applicable | not applicable | not applicable | not applicable | merged at their proximal tips by smooth connection | one lobe clearly larger than the other | no, shifted (e.g. Ophiacantha) | no, at least one lobe bent | with perforations | tilted | weakly developed | notch beyond the first segments under the disc | ventro-distalwards | no | no | more or less continuous ridge | with separate knob on ventral tip of LAP | more compact or more densely meshed stereom | without major kink and with tongue-shaped dorsal tip | not applicable | not applicable | vertical row without furrow |
| 34. Ophionereis porrecta | many thin scales | variable | no | same size/indistinguishable/absent | same size/indistinguishable/absent | not applicable | thin skin, not obscuring plates | without granules/spines | not applicable | not applicable | present | naked | less than one third of the disc radius | completely separated | scalene triangular | entire/continuous | distal-adradial portion of RS exposed | naked | longer than half interradius or divided into two openings | as long as adradial plate | bar-like without ridge or groove | concave | fully covered | disc granules | no | granule-like | covering less than one third of interradius | as long as wide | similar to other oral shields | no, separated | evenly convex | with narrower distalward projection | entering mouth slit via shallow embayment or opening deep wihtin mouth slit | no | single row along jaw edge | (fragmented into) several papillae (Ophiura) | infradental | scale-like, much larger/wider than other papillae | rounded | single | only at AS | with square tip | entire | equal width all over | single column throughout | surrounded by strongly protruding knobs and/or ridges | at least some perforating DP with septum | longer than high | large, well defined flange | normal stereom | with large, dorsal, spoon-shaped depression | naked | simple | potentially in contact | convex to straight | convex to straight | wider than proximal portion | yes | no | with tubercles or striation | no | multiple | in contact | oval semi-circular | tuberculous | no | lateral | predominantly erect, standing perpendicular to arm | 1-2 segments | massive | smooth | round | pointed | absent | not applicable | median spine(s) longest | decreasing distalwards | present | operculiform | accurately closing tentacle pore | without longitudinal striation | at both LAP and VAP | distalwards projecting beyond zygocondyles (e.g. Ophiothrix) | with single ridge | nearly parallel | present and fused with pair of zygocondyles | projecting beyond ventral edge of zygoc. with projecting part as long as zygoc. | yes (e.g. Ophiothrix) | arched | no | yes | no | protruding to form knobs larger than stereom pores on most of outer surface of LAP | absent | no | yes, only in central part | absent | present | not protruding | no | on same level as remaining outer surface | directly adjacent to the distal edge of the LAP | arranged over entire distal LAP edge | middle spine art. larger | dorsalwards increasing | by small ridge if at all | smaller than muscle opening | present | not applicable | not applicable | not applicable | not applicable | separated by one or several knobs or by denticulate stereom | one lobe clearly larger than the other | yes (e.g. Amphiura) | yes | with perforations | nearly horizontal | absent | notch beyond the first segments under the disc | ventro-distalwards | no | yes | more or less continuous ridge | with ventral tip of bentro-proximalwards pointing part of ridge separated from remaining ridge | more compact or more densely meshed stereom | with two kinks and dorsal kink with ventro-proximalwards pointing projection | not applicable | not applicable | single large and conspicuous |
| 35. Ophiopallas paradoxa | many thin scales | variable | no | same size/indistinguishable/absent | same size/indistinguishable/absent | not applicable | thin skin, not obscuring plates | with granules only | forming dense cover completely hiding underlying plates/scales (possible exception radial shields) | uniform | present | at least partly covered | less than one third of the disc radius | completely separated | isoscele triangular to pear-shaped | entire/continuous | distal-adradial portion of RS exposed | with granules | longer than half interradius or divided into two openings | as long as adradial plate | bar-like without ridge or groove | concave | fully covered | disc granules | no | spine-like | longer than one third of length of interradius | longer than wide | similar to other oral shields | no, separated | acute angle with straight to convex sides | with narrower distalward projection | entering mouth slit via shallow embayment or opening deep wihtin mouth slit | no | single row along jaw edge | (fragmented into) several papillae (Ophiura) | lateral | scale-like, much larger/wider than other papillae | rounded | single | none/only ASS | spine-shaped | entire | dorsal half widest | single column throughout | surrounded by a more or less continuous proturding ring | depression or perforating DP without septum | longer than high | central depression | normal stereom | ventral, lining ventral or ventro-distal edge of articulation area | naked | simple | potentially in contact | convex to straight | convex to straight | wider than proximal portion | yes | no | with tubercles or striation | no | single | in contact | trapezoid with smooth proximal edge | with striation | yes | lateral | predominantly parallel to arm axis (adpressed) | between half a segment and one segment | massive | with lateral thorns | round | pointed | only at distal segments | true, hyaline hook | median spine(s) longest | decreasing distalwards | present | operculiform | accurately closing tentacle pore | ? | only at LAP | distalwards projecting far from distal edge of zygocondyles (e.g. Ophiacantha) | with single ridge | dorsalwards converging | present and fused with pair of zygocondyles | not projecting beyond ventral edge of zygoc. or projecting beyond ventral edge of zygoc. with projecting part shorter than zygoc. | no | arched | no | yes | yes | protruding to form knobs larger than stereom pores on most of outer surface of LAP | formed by merged knobs | yes | yes, over most of the proximal edge | more than two small spurs | absent | not protruding | yes, along most of the edge | on same level as remaining outer surface | separated from distal edge by a thin projection of the distal LAP portion (e.g. Ophiomyces) | arranged over entire distal LAP edge | middle spine art. larger | dorsalwards increasing | by small ridge if at all | smaller than muscle opening | present | not applicable | not applicable | not applicable | not applicable | merged at their proximal tips by smooth connection | one lobe clearly larger than the other | no, shifted (e.g. Ophiacantha) | no, at least one lobe bent | massive | nearly vertical | absent | notch beyond the first segments under the disc | ventro-distalwards | no | yes | more or less continuous ridge | with ventral tip of bentro-proximalwards pointing part of ridge separated from remaining ridge | more compact or more densely meshed stereom | with kink between dorso-proximalwards pointing dorsal portion and ventro-proximalwards pointing ventral portion | not applicable | not applicable | small or inconspicuous |
| 36. Ophiopholis aculeata | many thin scales | variable | yes | larger than disc scales | same size/indistinguishable/absent | not applicable | thin skin, not obscuring plates | with both granules and spines | forming dense cover completely hiding underlying plates/scales (possible exception radial shields) | modified (e.g. enlarged) at disc edge | present | at least partly covered | between one third and half of the disc radius | completely separated | half-circle | entire/continuous | distal-adradial portion of RS exposed | with spines | longer than half interradius or divided into two openings | shorter than half the adradial plate length | half-ring-shaped | concave | fully covered | no | not applicable | not applicable | covering less than one third of interradius | wider than long | larger than remaining oral shields | yes | obtuse angle with straight to convex sides | evenly convex | entering mouth slit via shallow embayment or opening deep wihtin mouth slit | no | single row along jaw edge | (fragmented into) several papillae (Ophiura) | absent | scale-like, much larger/wider than other papillae | paddle-shaped | several | none/only ASS | with square tip | entire | ventral half widest | multiple columns or cluster on max half of plate | surrounded by strongly protruding knobs and/or ridges | at least some perforating DP with septum | as high as long or higher | large, well defined flange | rib-like branching structures | with large, dorsal, spoon-shaped depression | naked | simple | potentially in contact | concave or incised | concave or incised | as wide as proximal portion or narrower | yes | no | with tubercles or striation | no | multiple | in contact | oval semi-circular | tuberculous | no | lateral | predominantly erect, standing perpendicular to arm | 1-2 segments | massive | with lateral thorns | round | pointed | only at distal segments | true, hyaline hook | median spine(s) longest | decreasing distalwards | present | operculiform | not accurately closing tentacle pore | with longitudinal striation | only at LAP | distalwards projecting beyond zygocondyles (e.g. Ophiothrix) | with single ridge | dorsalwards converging | present and fused with pair of zygocondyles | not projecting beyond ventral edge of zygoc. or projecting beyond ventral edge of zygoc. with projecting part shorter than zygoc. | yes (e.g. Ophiothrix) | arched | no | yes | no | protruding to form knobs larger than stereom pores on most of outer surface of LAP | absent | no | yes, only in central part | absent | absent | protruding | no | on elevation not bordered by ridge | directly adjacent to the distal edge of the LAP | arranged over entire distal LAP edge | middle spine art. larger | dorsalwards increasing | by small ridge if at all | approx. as large as muscle opening | present | not applicable | not applicable | not applicable | not applicable | merged at their proximal tips by smooth connection | one lobe clearly larger than the other | yes (e.g. Amphiura) | no, at least one lobe bent | with perforations | tilted | absent | notch beyond the first segments under the disc | ventro-distalwards | no | no | two separate (rarely merged) central knobs | not applicable | not applicable | not applicable | with a ridge | yes | single large and conspicuous |
| 37. Ophioplax lamellosa | many thin scales | variable | no | larger than disc scales | same size/indistinguishable/absent | not applicable | thin skin, not obscuring plates | without granules/spines | not applicable | not applicable | present | naked | less than one third of the disc radius | completely separated | half-circle | entire/continuous | distal-adradial portion of RS exposed | with granules | longer than half interradius or divided into two openings | as long as adradial plate | bar-like without ridge or groove | concave | fully covered | disc granules | no | granule-like | longer than one third of length of interradius | as long as wide | similar to other oral shields | yes | acute angle with straight to convex sides | with narrower distalward projection | entering mouth slit via shallow embayment or opening deep wihtin mouth slit | no | single row along jaw edge | (fragmented into) several papillae (Ophiura) | infradental | scale-like, much larger/wider than other papillae | rounded | single | none/only ASS | with round or slightly pointed tip (but never spine-like) | entire | equal width all over | single column throughout | surrounded by separate, weakly protruding knobs and/or ridges | depression or perforating DP without septum | longer than high | central depression | normal stereom | in middle position, vertical and lining less than two thirds of distal edge of adradial articulation area | naked | simple | potentially in contact | convex to straight | convex to straight | wider than proximal portion | yes | no | without conspicuous ornamentation | no | single | in contact | oval semi-circular | without conspicuous ornamentation | no | lateral | predominantly erect, standing perpendicular to arm | 1-2 segments | massive | smooth | round | blunt | absent | not applicable | median spine(s) longest | decreasing distalwards | present | operculiform | accurately closing tentacle pore | without longitudinal striation | at both LAP and VAP | distalwards projecting almost beyond zygocondyles (e.g.. Ophiodoris) | with single ridge | dorsalwards converging | present and fused with pair of zygocondyles | projecting beyond ventral edge of zygoc. with projecting part longer than zygoc. | no | arched | no | yes | no | protruding to form only knobs approx. the same size as stereom pores | absent | no | yes, only in central part | absent | present | not protruding | yes, but restricted to small area (e.g. between spurs) | on same level as remaining outer surface | directly adjacent to the distal edge of the LAP | arranged over entire distal LAP edge | middle spine art. larger | dorsalwards increasing | by small ridge if at all | smaller than muscle opening | present | not applicable | not applicable | not applicable | not applicable | separated by one or several knobs or by denticulate stereom | one lobe clearly larger than the other | yes (e.g. Amphiura) | yes | with perforations | nearly horizontal | absent | notch beyond the first segments under the disc | ventro-distalwards | no | yes | more or less continuous ridge | with ventral tip of bentro-proximalwards pointing part of ridge separated from remaining ridge | more compact or more densely meshed stereom | with two kinks and dorsal kink with ventro-proximalwards pointing projection | not applicable | not applicable | single large and conspicuous |
| 38. Ophiopleura borealis | many thin scales | variable | no | larger than disc scales | same size/indistinguishable/absent | not applicable | thick skin, obscuring scales/plates | without granules/spines | not applicable | not applicable | present | naked | less than one third of the disc radius | completely separated | isoscele triangular to pear-shaped | entire/continuous | central part of RS to almost entire RS exposed | naked | shorter than half interradius | as long as adradial plate | bar-like with longitudinal ridge | straight or convex | fully covered | papillae | no | ? | covering less than one third of interradius | longer than wide | similar to other oral shields | yes | acute angle with straight to convex sides | evenly convex | entering mouth slit via shallow embayment or opening deep wihtin mouth slit | yes | single row along jaw edge | (fragmented into) several papillae (Ophiura) | lateral | scale-like or like other papillae | block-shaped | single | also at 1st VAP | spine-shaped | entire | equal width all over | single column throughout | surrounded by a more or less continuous proturding ring | depression or perforating DP without septum | longer than high | central depression | normal stereom | ventral, lining ventral or ventro-distal edge of articulation area | naked | simple | separated by lateral arm plates | convex to straight | convex to straight | wider than proximal portion | no | not applicable | without conspicuous ornamentation | no | single | in contact | trapezoid with smooth proximal edge | without conspicuous ornamentation | no | lateral | predominantly parallel to arm axis (adpressed) | shorter than half a segment | massive | smooth | round | pointed | absent | not applicable | median spine(s) longest | decreasing distalwards | present | operculiform | not accurately closing tentacle pore | without longitudinal striation | only at LAP | distalwards projecting far from distal edge of zygocondyles (e.g. Ophiacantha) | with single ridge | dorsalwards converging | present and fused with pair of zygocondyles | projecting beyond ventral edge of zygoc. with projecting part longer than zygoc. | no | arched | no | no | no | protruding to form only knobs approx. the same size as stereom pores | absent | no | yes, over most of the proximal edge | absent | present | not protruding | no | in notches of distal LAP edge | directly adjacent to the distal edge of the LAP | restricted to ventral or central portion of distal LAP edge | ventralwards increasing | dorsalwards increasing | by large, prominent ridge or regular stereom | approx. as large as muscle opening | absent (e.g. Ophiura) | no | simple stereom (e.g. Euryale), poorly defined circular elevation (e.g. Asteronyx) and/or vertical ridge distally and wavy ridge prox. (e.g. Gorgonocephalus) | oblique | slender | not applicable | not applicable | not applicable | not applicable | not applicable | not applicable | not applicable | notch beyond the first segments under the disc | distalwards, positioned close to the horizontal midline of the LAP | no | no | more or less continuous ridge | entire | same stereom as remaining inner surface of LAP | without major kink and with tongue-shaped dorsal tip | not applicable | not applicable | single large and conspicuous |
| 39. Ophioplinthus tessellata | thick scales | variable | no | larger than disc scales | larger than scales | at a distance from CPP | thin skin, not obscuring plates | without granules/spines | not applicable | not applicable | present | naked | between one third and half of the disc radius | completely separated | isoscele triangular to pear-shaped | entire/continuous | central part of RS to almost entire RS exposed | naked | longer than half interradius or divided into two openings | as long as adradial plate | bar-like with longitudinal ridge | straight or convex | exposed | papillae | no | block-like | longer than one third of length of interradius | as long as wide | similar to other oral shields | yes | acute angle with straight to convex sides | square | opening completely outside mouth slit | yes | single row along jaw edge | (fragmented into) several papillae (Ophiura) | lateral | scale-like or like other papillae | block-shaped | single | also at 1st VAP | with round or slightly pointed tip (but never spine-like) | entire | dorsal half widest | multiple columns or cluster on max half of plate | surrounded by a more or less continuous proturding ring | depression or perforating DP without septum | longer than high | central depression | normal stereom | ventral, lining ventral or ventro-distal edge of articulation area | naked | simple | separated by lateral arm plates | convex to straight | convex to straight | wider than proximal portion | yes | no | without conspicuous ornamentation | no | single | in contact | fan-shaped | without conspicuous ornamentation | no | lateral | predominantly parallel to arm axis (adpressed) | shorter than half a segment | massive | smooth | round | pointed | absent | not applicable | ventralmost spine(s) longest | decreasing distalwards | present | operculiform | not accurately closing tentacle pore | without longitudinal striation | only at LAP | no | with single ridge | dorsalwards converging | present and fused with pair of zygocondyles | projecting beyond ventral edge of zygoc. with projecting part as long as zygoc. | no | arched | no | no | no | protruding to form only knobs approx. the same size as stereom pores | absent | no | yes, over most of the proximal edge | more than two small spurs | present | not protruding | no | in notches of distal LAP edge | separated from distal edge by the usual outer surface stereom | arranged over entire distal LAP edge | all similar | dorsalwards increasing | by large, prominent ridge or regular stereom | approx. as large as muscle opening | absent (e.g. Ophiura) | yes | vertical mouth-shaped, sharply defined elevation | vertical | slender | not applicable | not applicable | not applicable | not applicable | not applicable | not applicable | not applicable | within-pore perforation beyond first segments under the disc | ventro-distalwards | no | ? | more or less continuous ridge | entire | more compact or more densely meshed stereom | without major kink and with tongue-shaped dorsal tip | not applicable | not applicable | small or inconspicuous |
| 40. Ophiopsila guineensis | many thin scales | uniform | no | same size/indistinguishable/absent | same size/indistinguishable/absent | not applicable | thin skin, not obscuring plates | without granules/spines | not applicable | not applicable | present | naked | between one third and half of the disc radius | completely separated | isoscele triangular to pear-shaped | entire/continuous | distal-adradial portion of RS exposed | naked | longer than half interradius or divided into two openings | longer than half the adradial plate length | bar-like without ridge or groove | concave | fully covered | no | not applicable | not applicable | covering less than one third of interradius | as long as wide | larger than remaining oral shields | yes | evenly convex | with narrower distalward projection | entering mouth slit via shallow embayment or opening deep wihtin mouth slit | no | single row along jaw edge | (fragmented into) several papillae (Ophiura) | infradental | scale-like or like other papillae | paddle-shaped | several | only at AS | with square tip | entire | dorsal half widest | single column throughout | surrounded by strongly protruding knobs and/or ridges | at least some perforating DP with septum | as high as long or higher | large, well defined flange | normal stereom | with large, dorsal, spoon-shaped depression | naked | simple | potentially in contact | concave or incised | convex to straight | wider than proximal portion | yes | no | without conspicuous ornamentation | no | single | in contact | oval semi-circular | without conspicuous ornamentation | no | lateral | predominantly erect, standing perpendicular to arm | 1-2 segments | massive | smooth | laterally flattened | blunt | absent | not applicable | ventralmost spine(s) longest | decreasing distalwards | present | spine-like | not accurately closing tentacle pore | with longitudinal striation | only at LAP | distalwards projecting almost beyond zygocondyles (e.g.. Ophiodoris) | with single ridge | dorsalwards converging | present and fused with pair of zygocondyles | projecting beyond ventral edge of zygoc. with projecting part as long as zygoc. | no | arched | no | yes | yes | not protruding | absent | no | yes, only in central part | absent | absent | not protruding | no | on same level as remaining outer surface | directly adjacent to the distal edge of the LAP | arranged over entire distal LAP edge | ventralwards increasing | dorsalwards increasing | by small ridge if at all | approx. as large as muscle opening | present | not applicable | not applicable | not applicable | not applicable | separated by one or several knobs or by denticulate stereom | equal-sized | yes (e.g. Amphiura) | yes | massive | nearly horizontal | absent | notch beyond the first segments under the disc | ventralwards | no | yes | two separate (rarely merged) central knobs | not applicable | not applicable | not applicable | simple | not applicable | vertical row without furrow |
| 41. Ophioscolex glacialis | very few thin small scales or none | not applicable | not applicable | same size/indistinguishable/absent | same size/indistinguishable/absent | not applicable | thick skin with few or no scales | without granules/spines | not applicable | not applicable | absent | not applicable | not applicable | not applicable | not applicable | not applicable | not applicable | naked | longer than half interradius or divided into two openings | shorter than half the adradial plate length | bar-like without ridge or groove | straight or convex | fully covered | no | not applicable | not applicable | covering less than one third of interradius | wider than long | similar to other oral shields | yes | obtuse angle with concave sides | evenly convex | opening completely outside mouth slit | no | single row along jaw edge | (fragmented into) several papillae (Ophiura) | absent | spiniform | spiniform | several | none/only ASS | spine-shaped | entire | ventral half widest | multiple columns or cluster on max half of plate | simple opening | depression or perforating DP without septum | longer than high | central depression | normal stereom | ventral, lining ventral or ventro-distal edge of articulation area | naked | simple | potentially in contact | convex to straight | convex to straight | wider than proximal portion | yes | no | without conspicuous ornamentation | no | none | not applicable | not applicable | not applicable | not applicable | lateral | predominantly erect, standing perpendicular to arm | 1-2 segments | massive | with lateral thorns | round | pointed | absent | not applicable | dorsalmost spine(s) longest | decreasing distalwards | absent | not applicable | not applicable | not applicable | not applicable | no | with single ridge | dorsalwards converging | present and fused with pair of zygocondyles | not projecting beyond ventral edge of zygoc. or projecting beyond ventral edge of zygoc. with projecting part shorter than zygoc. | no | arched | yes | yes | yes | not protruding | absent | no | yes, over most of the proximal edge | absent | present | not protruding | yes, along most of the edge | on same level as remaining outer surface | directly adjacent to the distal edge of the LAP | arranged over entire distal LAP edge | dorsalwards increasing | dorsalwards increasing | by small ridge if at all | smaller than muscle opening | present | not applicable | not applicable | not applicable | not applicable | merged at their proximal tips by smooth connection | one lobe clearly larger than the other | yes (e.g. Amphiura) | no, at least one lobe bent | with perforations | nearly horizontal | absent | notch beyond the first segments under the disc | ventralwards | no | yes | more or less continuous ridge | with ventral tip of bentro-proximalwards pointing part of ridge separated from remaining ridge | more compact or more densely meshed stereom | with kink between dorso-proximalwards pointing dorsal portion and ventro-proximalwards pointing ventral portion | not applicable | not applicable | small or inconspicuous |
| 42. Ophiosparte gigas | many thin scales | variable | no | same size/indistinguishable/absent | same size/indistinguishable/absent | not applicable | thick skin, obscuring scales/plates | without granules/spines | not applicable | not applicable | present | naked | less than one third of the disc radius | completely separated | isoscele triangular to pear-shaped | entire/continuous | central part of RS to almost entire RS exposed | naked | longer than half interradius or divided into two openings | longer than half the adradial plate length | bar-like with longitudinal ridge | straight or convex | exposed | papillae | yes | spine-like | covering less than one third of interradius | wider than long | similar to other oral shields | yes | obtuse angle with straight to convex sides | with narrower distalward projection | opening completely outside mouth slit | yes | single row along jaw edge | (fragmented into) several papillae (Ophiura) | ? | spiniform | spiniform | several | also at 1st VAP | spine-shaped | entire | equal width all over | multiple columns throughout | surrounded by a more or less continuous proturding ring | depression or perforating DP without septum | longer than high | central depression | normal stereom | ventral, lining ventral or ventro-distal edge of articulation area | naked | simple | potentially in contact | convex to straight | convex to straight | wider than proximal portion | no | not applicable | without conspicuous ornamentation | no | single | in contact | trapezoid with smooth proximal edge | without conspicuous ornamentation | no | lateral | predominantly erect, standing perpendicular to arm | 1-2 segments | massive | smooth | laterally flattened | blunt | absent | not applicable | dorsalmost spine(s) longest | constant | present | spine-like | not accurately closing tentacle pore | with longitudinal striation | only at LAP | distalwards projecting far from distal edge of zygocondyles (e.g. Ophiacantha) | with single ridge | nearly parallel | present and fused with pair of zygocondyles | projecting beyond ventral edge of zygoc. with projecting part longer than zygoc. | no | arched | no | no | no | not protruding | absent | no | yes, over most of the proximal edge | absent | absent | not protruding | no | on same level as remaining outer surface | directly adjacent to the distal edge of the LAP | restricted to ventral or central portion of distal LAP edge | all similar | dorsalwards increasing | by large, prominent ridge or regular stereom | approx. as large as muscle opening | absent (e.g. Ophiura) | no | vertical mouth-shaped, sharply defined elevation | vertical | slender | not applicable | not applicable | not applicable | not applicable | not applicable | not applicable | not applicable | notch beyond the first segments under the disc | distalwards, positioned close to the horizontal midline of the LAP | no | no | more or less continuous ridge | ridge separated into two halves | same stereom as remaining inner surface of LAP | without major kink and with tongue-shaped dorsal tip | not applicable | not applicable | vertical row without furrow |
| 43. Ophiosphalma fimbriatum | many thin scales | variable | no | larger than disc scales | larger than scales | at a distance from CPP | thin skin, not obscuring plates | without granules/spines | not applicable | not applicable | present | naked | more than half of the disc radius | completely separated | scalene triangular | entire/continuous | central part of RS to almost entire RS exposed | naked | longer than half interradius or divided into two openings | longer than half the adradial plate length | bar-like with longitudinal ridge | straight or convex | exposed | papillae | no | block-like | covering less than one third of interradius | longer than wide | similar to other oral shields | yes | acute angle with straight to convex sides | evenly convex | entering mouth slit via shallow embayment or opening deep wihtin mouth slit | no | single row along jaw edge | (fragmented into) several papillae (Ophiura) | lateral | scale-like or like other papillae | block-shaped | single | none/only ASS | with round or slightly pointed tip (but never spine-like) | entire | equal width all over | single column throughout | surrounded by a more or less continuous proturding ring | depression or perforating DP without septum | longer than high | central depression | normal stereom | ventral, lining ventral or ventro-distal edge of articulation area | naked | simple | separated by lateral arm plates | convex to straight | convex to straight | wider than proximal portion | yes | no | without conspicuous ornamentation | no | single | separated | fan-shaped | tuberculous | no | lateral | predominantly parallel to arm axis (adpressed) | shorter than half a segment | massive | smooth | round | blunt | absent | not applicable | ventralmost spine(s) longest | decreasing distalwards | present | operculiform | not accurately closing tentacle pore | ? | at both LAP and VAP | no | with single ridge | dorsalwards converging | present and fused with pair of zygocondyles | projecting beyond ventral edge of zygoc. with projecting part as long as zygoc. | no | arched | no | no | no | protruding to form knobs larger than stereom pores on most of outer surface of LAP | absent | no | yes, over most of the proximal edge | absent | present | not protruding | no | on same level as remaining outer surface | separated from distal edge by the usual outer surface stereom | arranged over entire distal LAP edge | ventralwards increasing | dorsalwards increasing | by large, prominent ridge or regular stereom | approx. as large as muscle opening | absent (e.g. Ophiura) | no | simple stereom (e.g. Euryale), poorly defined circular elevation (e.g. Asteronyx) and/or vertical ridge distally and wavy ridge prox. (e.g. Gorgonocephalus) | oblique | thick, lip-shaped and strongly protruding | not applicable | not applicable | not applicable | not applicable | not applicable | not applicable | not applicable | within-pore perforation beyond first segments under the disc | ventralwards | yes | ? | more or less continuous ridge | entire | more compact or more densely meshed stereom | without major kink and with tongue-shaped dorsal tip | not applicable | not applicable | small or inconspicuous |
| 44. Ophiotholia spatifer | many thin scales | uniform | no | ? | ? | ? | thin skin, not obscuring plates | without granules/spines | not applicable | not applicable | absent | not applicable | not applicable | not applicable | not applicable | not applicable | not applicable | naked | longer than half interradius or divided into two openings | as long as adradial plate | paddle-shaped | straight or convex | fully covered | no | not applicable | not applicable | covering less than one third of interradius | longer than wide | similar to other oral shields | no, separated | acute angle with straight to convex sides | with narrower distalward projection | entering mouth slit via shallow embayment or opening deep wihtin mouth slit | no | multiple rows covering jaws | (fragmented into) several papillae (Ophiura) | infradental | scale-like or like other papillae | paddle-shaped | single | also at 1st VAP | with round or slightly pointed tip (but never spine-like) | entire | ventral half widest | single column throughout | simple opening | depression or perforating DP without septum | longer than high | central depression | normal stereom | in middle position, vertical and lining less than two thirds of distal edge of adradial articulation area | naked | simple | separated by lateral arm plates | convex to straight | convex to straight | wider than proximal portion | yes | yes | without conspicuous ornamentation | no | single | separated | fan-shaped | without conspicuous ornamentation | no | lateral | predominantly erect, standing perpendicular to arm | 1-2 segments | massive | with lateral thorns | round | pointed | absent | not applicable | median spine(s) longest | decreasing distalwards | present | operculiform | not accurately closing tentacle pore | with longitudinal striation | at both LAP and VAP | distalwards projecting far from distal edge of zygocondyles (e.g. Ophiacantha) | with single ridge | nearly parallel | present and fused with pair of zygocondyles | not projecting beyond ventral edge of zygoc. or projecting beyond ventral edge of zygoc. with projecting part shorter than zygoc. | no | arched | yes | yes | yes | protruding to form only knobs approx. the same size as stereom pores | formed by regular ridges | no | yes, over most of the proximal edge | absent | present | not protruding | yes, but restricted to small area (e.g. between spurs) | on same level as remaining outer surface | separated from distal edge by a thin projection of the distal LAP portion (e.g. Ophiomyces) | arranged over entire distal LAP edge | ventralwards increasing | equidistant | by small ridge if at all | smaller than muscle opening | present | not applicable | not applicable | not applicable | not applicable | merged at their proximal tips by smooth connection | one lobe clearly larger than the other | yes (e.g. Amphiura) | no, at least one lobe bent | massive | nearly horizontal | absent | notch beyond the first segments under the disc | ventralwards | no | no | more or less continuous ridge | with ventral tip of bentro-proximalwards pointing part of ridge separated from remaining ridge | more compact or more densely meshed stereom | with kink between dorso-proximalwards pointing dorsal portion and ventro-proximalwards pointing ventral portion | not applicable | not applicable | vertical row without furrow |
| 45. Ophiothrix fragilis | many thin scales | uniform | yes | larger than disc scales | larger than scales | in contact with CPP | thin skin, not obscuring plates | with spines only | sparse all over with underlying plates/scales visible or restricted to margin | modified (e.g. enlarged) at disc edge | present | naked | more than half of the disc radius | completely separated | scalene triangular | entire/continuous | distal-adradial portion of RS exposed | with spines | longer than half interradius or divided into two openings | shorter than half the adradial plate length | half-ring-shaped | concave | fully covered | no | not applicable | not applicable | covering less than one third of interradius | wider than long | similar to other oral shields | no, separated | obtuse angle with straight to convex sides | evenly convex | entering mouth slit via shallow embayment or opening deep wihtin mouth slit | no | none | not applicable | not applicable | not applicable | not applicable | tooth papillae | none/only ASS | with round or slightly pointed tip (but never spine-like) | entire | ventral half widest | multiple columns or cluster on max half of plate | surrounded by strongly protruding knobs and/or ridges | at least some perforating DP with septum | as high as long or higher | large, well defined flange | rib-like branching structures | with large, dorsal, spoon-shaped depression | naked | simple | potentially in contact | concave or incised | concave or incised | wider than proximal portion | yes | no | with tubercles or striation | no | single | in contact | trapezoid with smooth proximal edge | tuberculous | no | lateral | predominantly erect, standing perpendicular to arm | longer than 2 segments | massive | with lateral thorns | round | pointed | at proximal and distal segments | true, hyaline hook | median spine(s) longest | decreasing distalwards | present | spine-like | not accurately closing tentacle pore | without longitudinal striation | only at LAP | distalwards projecting beyond zygocondyles (e.g. Ophiothrix) | with single ridge | dorsalwards converging | present and fused with pair of zygocondyles | not projecting beyond ventral edge of zygoc. or projecting beyond ventral edge of zygoc. with projecting part shorter than zygoc. | yes (e.g. Ophiothrix) | arched | no | yes | no | protruding to form knobs larger than stereom pores on most of outer surface of LAP | absent | no | yes, only in central part | absent | absent | protruding | no | on elevation not bordered by ridge | directly adjacent to the distal edge of the LAP | arranged over entire distal LAP edge | middle spine art. larger | dorsalwards increasing | by small ridge if at all | approx. as large as muscle opening | present | not applicable | not applicable | not applicable | not applicable | merged at their proximal tips by smooth connection | one lobe clearly larger than the other | yes (e.g. Amphiura) | no, at least one lobe bent | with perforations | tilted | absent | notch beyond the first segments under the disc | ventro-distalwards | no | no | two separate (rarely merged) central knobs | not applicable | not applicable | not applicable | with a ridge | no | single large and conspicuous |
| 46. Ophiotreta valenciennesi | many thin scales | uniform | no | same size/indistinguishable/absent | same size/indistinguishable/absent | not applicable | thin skin, not obscuring plates | with granules only | forming dense cover completely hiding underlying plates/scales (possible exception radial shields) | uniform | present | at least partly covered | between one third and half of the disc radius | completely separated | isoscele triangular to pear-shaped | entire/continuous | distal portion of RS exposed | with granules | longer than half interradius or divided into two openings | shorter than half the adradial plate length | bar-like without ridge or groove | concave | fully covered | no | not applicable | not applicable | covering less than one third of interradius | as long as wide | similar to other oral shields | yes | acute angle with straight to convex sides | with narrower distalward projection | entering mouth slit via shallow embayment or opening deep wihtin mouth slit | no | single row along jaw edge | (fragmented into) several papillae (Ophiura) | infradental | scale-like or like other papillae | rounded | several | only at AS | with round or slightly pointed tip (but never spine-like) | entire | ventral half widest | multiple columns or cluster on max half of plate | surrounded by a more or less continuous proturding ring | depression or perforating DP without septum | longer than high | central depression | normal stereom | ventral, lining ventral or ventro-distal edge of articulation area | naked | simple | potentially in contact | convex to straight | convex to straight | wider than proximal portion | yes | no | without conspicuous ornamentation | no | single | in contact | fan-shaped | without conspicuous ornamentation | no | lateral | predominantly erect, standing perpendicular to arm | longer than 2 segments | ? | with lateral thorns | laterally flattened | pointed | absent | not applicable | dorsalmost spine(s) longest | decreasing distalwards | present | operculiform | accurately closing tentacle pore | with longitudinal striation | only at LAP | distalwards projecting far from distal edge of zygocondyles (e.g. Ophiacantha) | with single ridge | dorsalwards converging | present and fused with pair of zygocondyles | not projecting beyond ventral edge of zygoc. or projecting beyond ventral edge of zygoc. with projecting part shorter than zygoc. | no | arched | yes | yes | no | protruding to form only knobs approx. the same size as stereom pores | formed by regular ridges | no | yes, over most of the proximal edge | absent | absent | not protruding | no | on elevated portion bordered prox. by ridge | separated from distal edge by the usual outer surface stereom | arranged over entire distal LAP edge | dorsalwards increasing | dorsalwards increasing | by small ridge if at all | smaller than muscle opening | present | not applicable | not applicable | not applicable | not applicable | merged at their proximal tips by smooth connection | one lobe clearly larger than the other | no, shifted (e.g. Ophiacantha) | no, at least one lobe bent | with perforations | tilted | fully developed | notch beyond the first segments under the disc | ventro-distalwards | no | no | more or less continuous ridge | with ventral tip of bentro-proximalwards pointing part of ridge separated from remaining ridge | more compact or more densely meshed stereom | with two kinks and dorsal kink with ventro-proximalwards pointing projection | not applicable | not applicable | vertical row with furrow |
| 47. Ophiozonella longispina | many thin scales | variable | no | larger than disc scales | larger than scales | at a distance from CPP | thin skin, not obscuring plates | without granules/spines | not applicable | not applicable | present | naked | between one third and half of the disc radius | completely separated | isoscele triangular to pear-shaped | entire/continuous | central part of RS to almost entire RS exposed | naked | longer than half interradius or divided into two openings | as long as adradial plate | bar-like without ridge or groove | concave | exposed | no | not applicable | not applicable | covering less than one third of interradius | as long as wide | similar to other oral shields | yes | acute angle with straight to convex sides | evenly convex | entering mouth slit via shallow embayment or opening deep wihtin mouth slit | no | single row along jaw edge | (fragmented into) several papillae (Ophiura) | infradental | scale-like, much larger/wider than other papillae | block-shaped | single | none/only ASS | with square tip | entire | equal width all over | single column throughout | surrounded by separate, weakly protruding knobs and/or ridges | at least some perforating DP with septum | longer than high | central depression | normal stereom | in middle position, vertical and lining more than two thirds of adradial articulation area | naked | simple | potentially in contact | convex to straight | convex to straight | wider than proximal portion | yes | no | with tubercles or striation | no | single | in contact | trapezoid with smooth proximal edge | tuberculous | yes | lateral | predominantly erect, standing perpendicular to arm | 1-2 segments | massive | smooth | round | pointed | absent | not applicable | dorsalmost spine(s) longest | decreasing distalwards | present | operculiform | accurately closing tentacle pore | without longitudinal striation | only at LAP | no | with single ridge | dorsalwards converging | present and fused with pair of zygocondyles | not projecting beyond ventral edge of zygoc. or projecting beyond ventral edge of zygoc. with projecting part shorter than zygoc. | no | arched | no | yes | yes | protruding to form knobs larger than stereom pores on most of outer surface of LAP | absent | no | yes, over most of the proximal edge | 1 or 2 small spurs | present | not protruding | yes, but restricted to small area (e.g. between spurs) | in notches of distal LAP edge | directly adjacent to the distal edge of the LAP | arranged over entire distal LAP edge | all similar | only 2 | by small ridge if at all | approx. as large as muscle opening | present | not applicable | not applicable | not applicable | not applicable | separated by one or several knobs or by denticulate stereom | equal-sized | yes (e.g. Amphiura) | yes | with perforations | nearly horizontal | absent | notch beyond the first segments under the disc | ventro-distalwards | no | no | more or less continuous ridge | with separate knob on ventral tip of LAP | same stereom as remaining inner surface of LAP | without major kink and with tongue-shaped dorsal tip | not applicable | not applicable | small or inconspicuous |
| 48. Ophiura ophiura | many thin scales | variable | no | larger than disc scales | larger than scales | at a distance from CPP | thin skin, not obscuring plates | without granules/spines | not applicable | not applicable | present | naked | between one third and half of the disc radius | completely separated | isoscele triangular to pear-shaped | entire/continuous | central part of RS to almost entire RS exposed | naked | longer than half interradius or divided into two openings | shorter than half the adradial plate length | bar-like with longitudinal ridge | straight or convex | exposed | papillae | yes | block-like | longer than one third of length of interradius | longer than wide | similar to other oral shields | no, separated | acute to right angle with convex sides | evenly convex | opening completely outside mouth slit | yes | single row along jaw edge | (fragmented into) several papillae (Ophiura) | infradental | scale-like or like other papillae | spiniform | several | also at 1st VAP | with round or slightly pointed tip (but never spine-like) | entire | equal width all over | multiple columns throughout | surrounded by a more or less continuous proturding ring | depression or perforating DP without septum | longer than high | central depression | normal stereom | ventral, lining ventral or ventro-distal edge of articulation area | naked | simple | separated by lateral arm plates | convex to straight | convex to straight | wider than proximal portion | no | not applicable | without conspicuous ornamentation | no | single | in contact | trapezoid with smooth proximal edge | without conspicuous ornamentation | no | lateral | predominantly parallel to arm axis (adpressed) | between half a segment and one segment | massive | smooth | laterally flattened | blunt | absent | not applicable | dorsalmost spine(s) longest | constant | present | operculiform | not accurately closing tentacle pore | without longitudinal striation | at both LAP and VAP | distalwards projecting far from distal edge of zygocondyles (e.g. Ophiacantha) | with single ridge | dorsalwards converging | present and fused with pair of zygocondyles | projecting beyond ventral edge of zygoc. with projecting part longer than zygoc. | no | arched | no | no | no | protruding to form only knobs approx. the same size as stereom pores | formed by merged knobs | no | yes, over most of the proximal edge | absent | present | not protruding | no | in notches of distal LAP edge | directly adjacent to the distal edge of the LAP | restricted to ventral or central portion of distal LAP edge | all similar | ventralwards increasing | by large, prominent ridge or regular stereom | approx. as large as muscle opening | absent (e.g. Ophiura) | yes | vertical mouth-shaped, sharply defined elevation | vertical | slender | not applicable | not applicable | not applicable | not applicable | not applicable | not applicable | not applicable | notch beyond the first segments under the disc | distalwards, positioned close to the horizontal midline of the LAP | no | no | more or less continuous ridge | ridge separated into two halves | same stereom as remaining inner surface of LAP | without major kink and with tongue-shaped dorsal tip | not applicable | not applicable | vertical row without furrow |
| 49. Palaeocoma milleri | many thin scales | variable | no | ? | ? | ? | ? | with granules only | forming dense cover completely hiding underlying plates/scales (possible exception radial shields) | uniform | present | naked | more than half of the disc radius | completely separated | isoscele triangular to pear-shaped | entire/continuous | central part of RS to almost entire RS exposed | with granules | longer than half interradius or divided into two openings | shorter than half the adradial plate length | bar-like with longitudinal ridge | straight or convex | exposed | papillae | yes | block-like | longer than one third of length of interradius | longer than wide | similar to other oral shields | yes | acute angle with straight to convex sides | square | entering mouth slit via shallow embayment or opening deep wihtin mouth slit | no | single row along jaw edge | (fragmented into) several papillae (Ophiura) | infradental | scale-like or like other papillae | block-shaped | single | only at AS | with round or slightly pointed tip (but never spine-like) | entire | equal width all over | multiple columns or cluster on max half of plate | ? | ? | longer than high | central depression | normal stereom | ventral, lining ventral or ventro-distal edge of articulation area | naked | simple | potentially in contact | concave or incised | convex to straight | wider than proximal portion | yes | no | without conspicuous ornamentation | no | single | in contact | trapezoid with smooth proximal edge | without conspicuous ornamentation | no | lateral | predominantly parallel to arm axis (adpressed) | shorter than half a segment | massive | smooth | laterally flattened | blunt | absent | not applicable | dorsalmost spine(s) longest | decreasing distalwards | present | operculiform | not accurately closing tentacle pore | without longitudinal striation | at both LAP and VAP | no | with single ridge | nearly parallel | present and fused with pair of zygocondyles | not projecting beyond ventral edge of zygoc. or projecting beyond ventral edge of zygoc. with projecting part shorter than zygoc. | no | arched | no | yes | no | protruding to form only knobs approx. the same size as stereom pores | absent | no | yes, over most of the proximal edge | more than two small spurs | present | not protruding | yes, but restricted to small area (e.g. between spurs) | in notches of distal LAP edge | directly adjacent to the distal edge of the LAP | arranged over entire distal LAP edge | all similar | ventralwards increasing | by large, prominent ridge or regular stereom | approx. as large as muscle opening | absent (e.g. Ophiura) | yes | vertical mouth-shaped, sharply defined elevation | vertical | slender | not applicable | not applicable | not applicable | not applicable | not applicable | not applicable | not applicable | notch beyond the first segments under the disc | ventro-distalwards | yes | no | more or less continuous ridge | entire | more compact or more densely meshed stereom | without major kink and with tongue-shaped dorsal tip | not applicable | not applicable | vertical row without furrow |
| 50. Sigsbeia murrhina | thick scales | variable | yes | larger than disc scales | larger than scales | at a distance from CPP | thin skin, not obscuring plates | without granules/spines | not applicable | not applicable | present | naked | more than half of the disc radius | completely separated | isoscele triangular to pear-shaped | ? | central part of RS to almost entire RS exposed | naked | shorter than half interradius | ? | ? | ? | ? | no | not applicable | not applicable | covering less than one third of interradius | wider than long | similar to other oral shields | yes | obtuse angle with straight to convex sides | evenly convex | entering mouth slit via shallow embayment or opening deep wihtin mouth slit | no | single row along jaw edge | single, wide papilla (Amphilepis) | lateral | scale-like or like other papillae | block-shaped | single | none/only ASS | with round or slightly pointed tip (but never spine-like) | ? | ? | ? | ? | ? | ? | ? | ? | ? | naked | simple | potentially in contact | convex to straight | concave or incised | wider than proximal portion | yes | no | with tubercles or striation | no | multiple | in contact | fan-shaped | tuberculous | no | at prox. segments only on ventral side of arm | predominantly parallel to arm axis (adpressed) | shorter than half a segment | massive | smooth | laterally flattened | blunt | absent | not applicable | all equal | constant | present | operculiform | accurately closing tentacle pore | ? | only at LAP | no | with single ridge | dorsalwards converging | absent | not applicable | no | arched | no | yes | no | protruding to form knobs larger than stereom pores on most of outer surface of LAP | absent | no | yes, only in central part | 1 or 2 large spurs | present | not protruding | yes, but restricted to small area (e.g. between spurs) | on same level as remaining outer surface | directly adjacent to the distal edge of the LAP | restricted to ventral or central portion of distal LAP edge | all similar | only 2 | by small ridge if at all | smaller than muscle opening | present | not applicable | not applicable | not applicable | not applicable | separated by one or several knobs or by denticulate stereom | equal-sized | yes (e.g. Amphiura) | yes | with perforations | nearly horizontal | absent | notch beyond the first segments under the disc | ventro-distalwards | no | no | more or less continuous ridge | with ventral tip of bentro-proximalwards pointing part of ridge separated from remaining ridge | same stereom as remaining inner surface of LAP | without major kink and with tongue-shaped dorsal tip | not applicable | not applicable | small or inconspicuous |
