# Supplementary material for: A New Morphological Phylogeny of the Ophiuroidea (Echinodermata) Accords with Molecular Evidence and Renders Microfossils Accessible for Cladistics
Source: PLoS One. 2016 May 26;11(5):e0156140. doi: 10.1371/journal.pone.0156140 (PMC4882042; doi:10.1371/journal.pone.0156140)
Supplement: S1 Table — Museum catalog numbers of all used specimens. (DOCX) [file pone.0156140.s011.docx]

| Species | Voucher catalog no. |
| --- | --- |
| Aganaster gregarius | MnhnL OPH028 |
| Amphilepis norvegia | MnhnL OPH027 |
| Amphilimna olivacea | SMNH-108487, 108493, 108766, 133293 |
| Amphioplus congensis | SMNH-108477, 108479, 108481 |
| Amphiura chiajei | SMNH-52135, 52139, 67815, 90612, 105100 |
| Aplocoma agissizi | NCB-RGM 344 003 to 344 012 |
| Asteronyx loveni | SMNH-67680, 67752, 87701, 87702, 90605; MnhnL OPH013 |
| Eirenura paillata | MnhnL PL220, PL228, PL239, PL240, PL242; MNHN A30316, A30317. |
| Euryale aspera | SMNH-109400, 131553-61 |
| Histampica duplicata | SMNH-131573, 131574 |
| Inexpectancantha acrobatica | MnhnL PL221, PL224, PL222, PL227, PL229, PL239-16, , PL241, PL242; MNHN A30315, |
| Ophiacantha bidentata | SMNH-21409, 45856, 46106, 90464, 111042-44 |
| Ophiactis savignyi | SMNH-41241, 41248, 41249; MnhnL OPH011, OPH023 |
| Ophiarachna incrassata | SMNH-121142; MnhnL OPH001, OPH008, OPH016, OPH021, OPH024 |
| Ophiengima spinilimbatum | SMNH-127068 |
| Ophiochiton fastigatus | MnhnL OPH025 |
| Ophiocoma echinata | MnhnL OPH002 |
| Ophiocomina nigra | SMNH-20988, 42837, 42905, 42906, 90651 |
| Ophiocopa spatula | SMNH-122912, 123454, 131566 |
| Ophiocten sericeum | MnhnL OPH009, OPH022 |
| Ophioderma longicauda | SMNH-42841, 90215, 133258, |
| Ophiodoris malignus | MNHN IE.2009.623, EcOs 23113; MnhnL OPH004, OPH010, OPH012, OPH017, OPH018 |
| Ophioleuce seminudum | SMNH-118750, 121772; MNHN Ech Os 22613 |
| Ophiolimna bairdi | SMNH-42828, 42829, 45708, 90625 |
| Ophiolycus purpureus | SMNH-45695, 45849, 90262, 111007, 111023 |
| Ophiomusium lymani | SMNH-130490, 130493, 130498-500; MnhnL OPH020 |
| Ophiomyces delata | SMNH-131571, 133295; MnhnL OPH015 |
| Ophiomyxa pentagona | SMNH-90260, 111004-06, 111024 |
| Ophionereis porrecta | SMNH-111008, 111009, 111047, 121358 |
| Ophiopallas paradoxa | SMNH-90640, 109483 |
| Ophiopholis aculeata | SMNH-52111, 90615, 111040, 111041, 133291; MnhnL OPH014 |
| Ophioplax lamellosa | SMNH-130491, 130497, 131575, 131576 |
| Ophiura ophiura | MnhnL OPH003, OPH007, OPH019 |
| Ophiothrix fragilis | SMNH-111046 |
| Ophiotholia spathifer | MnhnL OPH005 |
| Ophiotreta valenciennesi | MnhnL OPH026 |
| Palaeocoma milleri | MnhnL OPH006 |

Collection acronyms:

MnhnL: Natural History Museum Luxembourg

MNHN: National Museum of Natural History, Paris

NCB-RGM: Naturalis Biodiversity Center, Leiden

SMNH: Swedish Museum of Natural History
